# Accounting for centre-effects in multicentre trials with a binary outcome – when, why, and how?

**DOI:** 10.1186/1471-2288-14-20

**Published:** 2014-02-10

**Authors:** Brennan C Kahan

**Affiliations:** 1 Pragmatic Clinical Trials Unit, Queen Mary University of London, 58 Turner Street, London E1 2AB, UK; 2 MRC Clinical Trials Unit at UCL, 125 Kingsway, London WC2B 6NH, UK

**Keywords:** Binary outcomes, Randomised controlled trial, Multicentre trials, Fixed-effects, Random effects, Generalised estimating equations, Mantel-Haenszel

## Abstract

**Background:**

It is often desirable to account for centre-effects in the analysis of multicentre randomised trials, however it is unclear which analysis methods are best in trials with a binary outcome.

**Methods:**

We compared the performance of four methods of analysis (fixed-effects models, random-effects models, generalised estimating equations (GEE), and Mantel-Haenszel) using a re-analysis of a previously reported randomised trial (MIST2) and a large simulation study.

**Results:**

The re-analysis of MIST2 found that fixed-effects and Mantel-Haenszel led to many patients being dropped from the analysis due to over-stratification (up to 69% dropped for Mantel-Haenszel, and up to 33% dropped for fixed-effects). Conversely, random-effects and GEE included all patients in the analysis, however GEE did not reach convergence. Estimated treatment effects and p-values were highly variable across different analysis methods.

The simulation study found that most methods of analysis performed well with a small number of centres. With a large number of centres, fixed-effects led to biased estimates and inflated type I error rates in many situations, and Mantel-Haenszel lost power compared to other analysis methods in some situations. Conversely, both random-effects and GEE gave nominal type I error rates and good power across all scenarios, and were usually as good as or better than either fixed-effects or Mantel-Haenszel. However, this was only true for GEEs with non-robust standard errors (SEs); using a robust ‘sandwich’ estimator led to inflated type I error rates across most scenarios.

**Conclusions:**

With a small number of centres, we recommend the use of fixed-effects, random-effects, or GEE with non-robust SEs. Random-effects and GEE with non-robust SEs should be used with a moderate or large number of centres.

## Background

In randomised controlled trials (RCTs) with multiple centres, we sometimes expect patient outcomes to differ according to centre. This could be due to differences between patients who present to different centres, or because of differences between the centres themselves. Because of this, many RCTs attempt to minimise the impact of any between-centre differences on the trial results, either during the design stage (by stratifying on centre in the randomisation process, ensuring an equal number of patients assigned to both treatment groups within each centre), or during the analysis stage (by accounting for centre-effects in the analysis model).

Recent research has shown that when randomisation is stratified by centre, it is necessary to account for the centre-effects in the analysis as well. This is because stratified randomisation leads to correlation between treatment arms, making it necessary to adjust for the stratification factors in the analysis to obtain correct confidence intervals and p-values, maintain the type I error rate at its nominal level (usually set at 5%), and avoid a reduction in power [1-5]. Therefore, any attempt to minimise between-centre differences will necessarily lead to an analysis that accounts for centre-effects.

However, adjustment for centre-effects in the analysis can often be problematic, particularly when there are a large number of centres compared to the overall sample size. This can cause problems with binary or time-to-event outcomes, where too few patients or events per centre can lead to biased estimates [6] or inflated type I error rates [1] with some methods of analysis.

The goals of this paper are to clarify (a) under what circumstances it is beneficial to account for centre-effects in the analysis, and (b) the best method of adjustment for centre in RCTs with a binary outcome. The case of multicentre trials with continuous [3,7-10] or time-to-event [11] outcomes has been discussed previously. We restrict our attention to analysis methods which calculate an odds ratio, and do not consider the issue of treatment-by-centre interaction.

## Methods

### When should we adjust for centre-effects?

Whether it will be beneficial to account for centre-effects in the analysis depends on (a) the size of the intraclass correlation coefficient (ICC), and (b) the extra complexity added to the analysis through adjustment. We discuss these issues in the two sections below.

### ICC considerations

The ICC measures to what extent the proportion of the total variability is explained by the variability between the different centres. For a binary outcome analysed using an odds ratio (OR), the ICC can be defined as σ^2^/(σ^2^ + π^2^/3), where σ^2^ is the between-centre variance [12].

When centre is not accounted for in the analysis, the standard error for treatment effect is increased by a factor of (1-ICC)^-1/2^, leading to a reduction in power [4]. For example, in a trial designed to give 80% power, ICCs of 0.01, 0.10, and 0.25 would lead to reductions in power of 0.4, 4.3, and 12.1% respectively if centre was ignored in the analysis.

ICC estimates based on previous data may be helpful in determining whether adjustment will be beneficial. For example, Adams *et al.*[13] reviewed ICCs from 1039 variables across 31 primary care studies and found the median ICC was 0.01 (IQR 0 to 0.03), indicating that adjustment for centre-effects in these scenarios may not materially increase power. Conversely, Cook *et al.*[14] reviewed ICCs from 198 outcomes across 10 surgical trials, and found 44% of ICCs were greater than 0.05, indicating that adjustment may be more beneficial in these trials.

However, previous data may not be available in many cases. It may then be useful to consider two issues. The first issue is whether centres are likely to influence patient outcomes. For example, in a surgical intervention trial, surgeons may be more skilled in some centres than in others, leading to improved patient outcomes in those centres. Alternatively, some outcomes may be influenced by centre policy; for example, an outcome such as length of hospital stay may have a large ICC because some hospitals have a policy of early discharge whereas others do not. Conversely, in a drug intervention trial where the primary function of centre is to dispense the treatment to patients, it is unlikely that centre would have any material influence on patient outcomes.

The second issue to consider is whether baseline risk is likely to vary substantially across centres. For example, in an international trial assessing an intervention to reduce neonatal mortality, patient risk levels are likely to differ between centres in different countries, leading to a high ICC. However, if the trial was taking place in a single country the ICC would likely be much lower.

### Added complexity through adjustment for centre

In some situations adjustment for centre can lead to an extremely complex analysis model which may not work well in practice. Consider a multicentre trial where surgeons treat patients in one treatment group only, and patients are followed up at several time points. It is necessary in this scenario to account for patients being clustered within surgeon, and for the multiple follow-up measurements, as these are both forms of *non-ignorable* clustering, and could lead to inflated type I error rates if ignored [5]. Accounting for centre in the analysis would lead to a complicated four-level model (observations nested within patients nested within surgeons nested within centres) which may not converge, or may give unstable estimates. Therefore, unless the ICC is expected to be very large, it may be counterproductive to adjust for centre-effects in this scenario.

### Implications of stratified vs unstratified randomisation

The implications of adjusting (or not adjusting) for centre-effects depend on whether centre has been used as a stratification factor in the randomisation process.

If centre has been used as a stratification factor, we recommend that centre-effects be accounted for as a default position (regardless of the expected ICC) to ensure that p-values and confidence intervals are unbiased [1-4]. The exception to this is when it is expected that adjusting for centre-effects could lead to convergence issues, or unstable estimates; in this case, we recommend centre be ignored in the analysis, as non-convergence or unstable estimates are a larger danger than a type I error rate that is too low.

When centre has not been used as a stratification factor, adjusted and unadjusted analyses will both give unbiased p-values and confidence intervals; however, an adjusted analysis will lead to increased power when the ICC is large. Consequently, it is somewhat less important to adjust for centre-effects than after stratified randomisation, as results will be valid regardless. Therefore, we recommend that centre be accounted for in the analysis if (a) the ICC is expected to be large enough to materially increase power; *and* (b) it is not anticipated that adjustment for centre-effects will impact convergence rates or stability of treatment effect estimates.

### Marginal vs conditional models

Centre-effects can be accounted for in the analysis using either a marginal (or population averaged) approach, or a conditional (or centre specific) approach. For binary outcomes, these two approaches lead to different odds ratio and different interpretations.

A conditional approach compares the change in the odds for a treated patient vs. a control patient from the same centre. In contrast, the marginal approach compares the change in odds for a treated patient vs. a control patient who has been randomly selected from *any* centre in the trial. Because these two approaches are comparing different things, the actual treatment effect estimates will differ (provided there is a treatment effect; when the treatment is not effective, both methods will give similar estimates) [15,16]. In general, odds ratios from a marginal model tend to be smaller (i.e. closer to the null) than estimates from a conditional model. The size of the discrepancy between the two approaches is influenced by the ICC; the larger the ICC, the larger the difference of the two estimates. For an ICC of 0, the size of the treatment effect is the same for both approaches (as in this case, there is no difference between patients in different centres, and so both approaches are answering the same question).

### Conditional models

#### Mantel-Haenszel

Mantel-Haenszel (MH) is a within-centre comparison, where the treatment effect is calculated within each centre and then combined for an overall effect [6]. MH can be calculated as:

$$OR_{\mathrm{MH}}=\;\frac{\sum_{j}\frac{a_{j}d_{j}}{n_{j}}}{\sum_{j}\frac{b_{j}c_{j}}{n_{j}}}$$

where *j* denotes the centre, a_j_ and b_j_ indicate the number of patients with and without an event in the intervention group respectively, c_j_, and d_j_ indicate the number of patients with and without an event in the control group respectively, and n_j_ is the total number of patients in that centre.

One of the drawbacks of MH is that centres for which a treatment effect cannot be calculated are excluded from the analysis. This occurs when all patients in a centre experience the same outcome (e.g. all successes or all failures), or when all patients in a centre are randomised to the same treatment group. These scenarios are most likely to occur with a small number of patients in a centre. Therefore, in trials where many centres have relatively few patients, MH may exclude a large proportion of patients from the analysis, leading to a reduction in both power and precision.

MH can account for other covariates in the analysis; this is done by forming strata from all combinations of covariates (including centre), then estimating the treatment effect within each stratum. This implies that covariates must be categorical in order to be included in a MH analysis, meaning that continuous covariates must be categorised. However, categorisation of continuous covariates may reduce power. Furthermore, this can easily lead to a large number of strata (e.g. 10 centres, and three binary covariates would lead to 10×2×2×2 = 80 strata), which increases the chances of some strata being dropped from the analysis.

MH assumes a large sample size, however it does not assume that the sample size is large compared to the number of centres; therefore, when there is a small number of patients in each centre, MH should still give unbiased estimates of treatment effect, and correct type I error rates.

#### Fixed-effects

A fixed-effects analysis fits a logistic regression model which includes an indicator variable for all centres but one. The model can be written as:

$$\log\;\mathrm{it}\left(\pi_{\mathrm{ij}}\right)=\alpha +\beta_{\mathrm{treat}}X_{\mathrm{ij}}+\beta_{C_{1}}+\beta_{C_{2}}+\ldots +\beta_{C_{j-1}}$$

where *π*_*ij*_ is the probability of an event for the *i*th patient in the *j*th centre, *β*_*treat*_ indicates the log odds ratio for treatment, X_ij_ indicates whether the patient received the treatment or control, and the β_C_’s indicate the effect of the j^th^ centre.

A fixed-effects analysis has similar drawbacks to Mantel-Haenszel in that it excludes centres where all patients experience the same outcome, or where all patients are randomised to the same treatment group. However, unlike MH, fixed-effects can include continuous covariates in the analysis without the need for categorisation, and so may lead to increased power compared to MH when adjusting for other covariates besides centre.

A fixed-effects analysis assumes the overall sample size is large; however, unlike Mantel-Haenszel, fixed-effects assumes that the sample size compared to the number of centres is also large [6]. When this assumption is not met (i.e. when there is a small number of patients per centre) fixed-effects can give biased estimates of treatment effect [6] or inflated type I error rates [1].

#### Random effects

A random-effects analysis involves fitting a mixed-effects logistic regression model:

$$\log\;\mathrm{it}\left(\pi_{\mathrm{ij}}\right)=\alpha +\beta_{\mathrm{treat}}X_{\mathrm{ij}}+u_{j}$$

where u_j_ is the effect of the j^th^ centre, and is generally assumed to follow a normal distribution with mean 0 and variance σ^2^.

A random-effects analysis is able to include all centres in the analysis, even when all patients in that centre are randomised to the same treatment group, or experience the same outcome. Random-effects can also account for continuous covariates in the analysis, without the need for categorisation. Random-effects assumes a large sample size, but like Mantel-Haenszel, does not assume a large sample size compared to the number of centres, and should therefore give valid results even when there is a small number of patients in each centre.

Random-effects models generally make the assumption that the centre-effects follow a normal distribution (although other distributions could be used). This may not be known in advance, and may be an unrealistic assumption; however, research has shown that inference for the fixed effects (i.e. the treatment effect) are quite robust to the assumption that the centre-effects follow a normal distribution, and so a mixed-effects logistic regression model is likely to give valid inference for the treatment effect even when the centre-effects are not normally distributed [3,17,18].

### Marginal models

#### Generalised estimating equations

Generalised estimating equations (GEEs) [19] are the most popular method of analysing correlated binary data using a marginal model. A GEE analysis fits the model:

$$\log\;\mathrm{it}\left(\pi_{i}\right)=\alpha +\beta_{\mathrm{treat}}X_{i}$$

where *π*_*i*_ is the probability of an event for the i^th^ patient, *β*_*treat*_ indicates the log odds ratio for treatment, and X_i_ indicates whether the patient received the treatment or control.

GEEs allow the user to specify the correlation structure between observations. The most intuitive structure for a multicentre trial is an exchangeable correlation structure, which assumes outcomes are correlated for patients in the same centre, but are independent for patients in different centres. There are two primary methods of calculating standard errors (SEs) for GEEs. The first method relies on the specified correlation structure being correct (non-robust SEs). The second method uses a robust sandwich estimator to estimate the standard error (robust SEs). This method is robust to misspecification of the correlation structure, and will give valid type I error rates even if the chosen correlation structure is incorrect [19]. However, it may lose efficiency compared to non-robust SEs.

Robust SEs can be extremely useful when analysing longitudinal data (where patients are followed-up at multiple time points), as this type of data leads to many possible correlation structures, and it may be difficult or impossible to know which is correct. However, in multicentre trials, correlation structures other than exchangeable are unlikely; therefore, we focus mainly on non-robust SEs in this article.

Similarly to random-effects, GEEs are able to include all centres in the analysis, even centres where all patients experience the same outcome, or are randomised to the same treatment arm.

#### Application to the MIST2 trial

We now compare different methods of analysis using data from a previously published RCT. The MIST2 trial was a four arm trial which compared tPA, DNase, and tPA plus DNase to placebo in patients with a pleural infection [20]. We consider the number of patients undergoing surgical intervention up to 90 days. Of 190 patients included in the analysis, 23 (12%) experienced an event.

Patients were recruited from 11 centres, with a median of 12 patients per centre (range 1 to 87). Two centres recruited patients to only one arm, and one centre recruited patients to only two arms (all other centres recruited patients to each of the four arms). In four centres all recruited patients experienced the same outcome (no surgery), whereas in all other centres patients experienced both outcomes.

Centre was not used as a stratification factor in the randomisation process in this trial, and so it is not strictly necessary to account for centre in the analysis to obtain valid results (p-values and confidence intervals will be correct regardless). Therefore, we compared five different analysis methods; a logistic regression model that was unadjusted for centre-effects, fixed-effects, random-effects, GEE, and Mantel-Haenszel. We also accounted for the minimisation variables in each analysis [1-3,5], which were the amount of pleural fluid in the hemithorax at baseline as a continuous variable, whether the infection was hospital acquired or not, and whether there was evidence of locuation. For Mantel-Haenszel we dichotomised the amount of pleural fluid at less or greater than 30%. This led to 42 strata (as some combinations of covariates had no patients).

Results are shown in Table 1. Using random-effects, GEEs, or a logistic regression model that ignored centre-effects allowed all patients to be included in the analysis. In comparison, fixed-effects dropped between 19-33% of patients from the analysis (depending on the treatment comparison), and Mantel-Haenszel dropped between 52-69% of patients. Convergence was not achieved for GEE, although Stata still provided results. All other analysis methods achieved convergence.

**Table 1:** Analysis results from the MIST2 dataset

	**tPA vs placebo (n = 98)**	**DNase vs placebo (n = 94)**	**tPA+DNase vs placebo (n = 98)**
Unadjusted for centre-effects			
Patients included – no. (%)	98 (100)	94 (100)	98 (100)
Odds ratio (95% CI)	0.42 (0.09 to 1.87)	2.72 (0.88 to 8.42)	0.23 (0.04 to 1.29)
P-value	0.25	0.08	0.10
Fixed-effects			
Patients included – no. (%)	66 (67)	80 (85)	81 (83)
Odds ratio (95% CI)	0.54 (0.12 to 2.56)	3.34 (1.01 to 11.00)	0.24 (0.04 to 1.36)
P-value	0.44	0.048	0.11
Random-effects			
Patients included – no. (%)	98 (100)	94 (100)	98 (100)
Odds ratio (95% CI)	0.42 (0.09 to 1.87)	2.72 (0.88 to 8.42)	0.23 (0.04 to 1.29)
P-value	0.25	0.08	0.10
GEE*			
Patients included – no. (%)	98 (100)	94 (100)	98 (100)
Odds ratio (95% CI)	0.33 (0.06 to 1.75)	2.57 (0.76 to 8.66)	0.18 (0.03 to 1.19)
P-value	0.19	0.13	0.08
Mantel-Haenszel			
Patients included – no. (%)	30 (31)	45 (48)	35 (36)
Odds ratio (95% CI)	0.34 (0.05 to 2.15)	3.59 (0.99 to 13.03)	0.21 (0.02 to 1.98)
P-value	0.27	0.04	0.13

*Convergence was not achieved.

The different analysis methods led to different treatment effect estimates. Estimated odds ratios varied between 0.33-0.54 for the tPa vs placebo comparison, and between 2.57-3.59 for the DNase vs placebo comparison. Different analysis methods also led to different p-values in some instances. P-values ranged from 0.04 to 0.13 for the DNase vs placebo comparison, demonstrating that the choice of analysis method can substantially affect trial results and interpretations. Results from random-effects and a logistic regression model that ignored centre gave nearly identical results; this was because the estimated ICC was nearly 0.

It should be noted that the p-value and confidence interval from Mantel-Haenszel was inconsistent for the DNase vs placebo comparison. The p-value indicated a statistically significant result (p = 0.04), whereas the confidence interval did not (95% CI 0.99 to 13.03). This is likely a result of the Mantel-Haenszel procedure in Stata using different information to calculate the p-value and confidence interval.

### Simulation study

We performed a simulation study to compare fixed-effects, random-effects, GEEs with an exchangeable correlation structure and non-robust SEs, and Mantel-Haenszel in terms of the estimated treatment effect, type I error rate, power, and convergence rates. The first set of simulations was based on the MIST2 trial. In the second set of simulations we varied a number of different parameters (e.g. number of centres, number of patients per centre, ICC, etc.) to compare the methods of analysis across a wide range of plausible scenarios.

For each analysis method we calculated the mean treatment effect estimate, the type I error rate (proportion of false-positives), power (proportion of true-positives), and the convergence rate. The mean treatment effect was calculated as the exponential of the mean of the log odds ratios for treatment. The type I error rate was calculated as the proportion of replications which gave a statistically significant result (P < 0.05), when the true odds ratio for treatment was 1. Power was calculated as the proportion of replications which gave a statistically significant result, when the true odds ratio for treatment was not 1. We set convergence as having failed when either (i) STATA gave an error message indicating the analysis did not converge; (ii) the absolute value of either the log odds ratio for treatment or its standard error was greater than 1000; or (iii) the estimates of the log odds ratio for treatment or its standard error SE was set to exactly 0 (as this indicates STATA dropped treatment from the model). We only assessed the mean treatment effect, type I error rate, and power when convergence was achieved.

We used 5000 replications for each scenario to give a standard error of about 0.3% when estimating the type I error rate, assuming a true type I error rate of 5%. We performed all simulations using STATA 12.1 (StataCorp, College Station, TX, USA).

### Simulations based on MIST2

For simplicity, we considered only two treatment groups, rather than four. Data were generated from the following model:

$$\begin{array}{l} Y_{\mathrm{ij}}^{*}=\alpha +\beta_{\mathrm{treat}}X_{\mathrm{ij}}+\beta_{\mathrm{pleural}\_\mathrm{fluid}}Z1_{\mathrm{ij}}+\beta_{\mathrm{purulence}}Z2_{\mathrm{ij}}\\ \;+\beta_{\mathrm{hospital}\_\inf\mathrm{ection}}Z3_{\mathrm{ij}}+u_{j}+\epsilon_{\mathrm{ij}} \end{array}$$

where Y_ij_^*^ is a latent outcome for i^th^ patient in the j^th^ centre, *β*_*treat*_ is the log odds ratio for treatment effect, X_ij_ indicates whether the patient received the treatment or control, *β*_*pleural_fluid*_, *β*_*purulence*_, and *β*_*hospital_infection*_ are covariate effects, and Z1_ij_, Z2_ij_, and Z3_ij_ are the covariate values. u_j_ is the centre effect for j^th^ centre and follows a normal distribution with mean 0 and standard deviation σ, and ϵ_ij_ is a random error term that follows a logistic distribution with mean 0 and variance π^2^/3. Binary responses were generated as Y_ij_ = 1 if Y_ij_^*^ > 0, and 0 otherwise. We generated μ_j_ and ϵ_ij_ independently.

All parameters were based on estimates from the MIST2 dataset. We set the odds ratio for treatment (exp(*β*_*treat*_)) to 1 (indicating no effect) to evaluate the type I error rate, and to 0.23 to evaluate power (this was similar to the OR for the tPA+DNase comparison). The ICC was set to 0 (equivalent to setting σ = 0). The log odds ratios for *β*_*pleural_fluid*_, *β*_*purulence*_, and *β*_*hospital_infection*_ were 0.03, -0.2, and 0.4 respectively, and the log odds for α was −3.3.

We generated the covariates (Z1_ij_, Z2_ij_, and Z3_ij_), and centre by sampling with replacement from the MIST2 dataset, to ensure the correlation structure between covariates was preserved. Patients were assigned to one of the two treatment groups using simple randomisation. Because centre was not used as a stratification factor, we also analysed data using a logistic regression model which did not account for centre-effects (although did adjust for the other covariates).

### Simulations based on varied parameters

Data were generated from the following model:

$$Y_{\mathrm{ij}}^{*}=\alpha +\beta_{\mathrm{treat}}X_{\mathrm{ij}}+u_{j}+\epsilon_{\mathrm{ij}}$$

where Y_ij_^*^, *β*_*treat*_, X_ij_, u_j_ and ϵ_ij_ are the same as the previous section.

We varied the following parameters:

• *Number of centres*: we used 5, 50, and 100 centres

• *Number of patients*: we used overall sample sizes of 200, 500, 1000, and 2000 patients

• *ICC*: we used ICC values of 0.025 and 0.075 (we set σ_j_ to give the desired ICC)

• *Patient distribution across centres*: we used two patient distributions across centres. In the first, each centre had an equal number of patients (even patient distribution). In the second, most patients were concentrated in a small number of centres, and the remaining centres had relatively few patients (skewed patient distribution). The exact number of patients in each centre in the skewed patient distribution can be found in Additional file 1.

• *Event rate*: we used event rates in the control group of 20% and 50%

• *Randomisation method*: we used permuted blocks, stratified by centre. We used block sizes of 4 and 20.

In total, we evaluated 192 different scenarios. The parameter values above were selected to give a wide range of plausible trial scenarios.

We set the log odds ratio for treatment to 0 to evaluate the type I error rate. In order to evaluate power, we set the log odds ratio for treatment to give 80% power based on the sample size and the event rate. Power was calculated based on reducing (rather than increasing) the number of events.

### Sensitivity analysis – large ICC

We performed a sensitivity analysis using an ICC of 0.25. We set the event rate to 50%, and used an even patient distribution. We varied the number of centres (5, 50, and 100), the number of patients per centre (200, 500, 1000, and 2000), and the block size (4 or 20). This led to 24 scenarios in total. We used the same methods of analysis as above (fixed-effects, random-effects, GEE, and Mantel-Haenszel).

### Sensitivity analysis – GEE with a robust variance estimator

We performed another sensitivity analysis assessing the use of GEE with a robust variance estimator (robust SEs). We used an ICC of 0.025, set the event rate to 50%, used an even patient distribution, and used stratified permuted blocks with a block size of four. We varied the number of centres (5, 50, and 100), the number of patients per centre (200, 500, 1000, and 2000). This led to 12 scenarios in total.

## Results

### Simulations based on MIST2

Results are shown in Tables 2 and 3. When the OR for treatment effect was set to 1, all analysis methods had convergence rates near 100%. Estimated treatment effects were unbiased for each analysis method, and all methods gave close to nominal type I error rates. The lone exception was Mantel-Haenszel, which gave a type I error rate of 4.0%.

**Table 2:** Results from simulations based on MIST2 dataset (OR = 1)

	**Mean treatment effect**	**Type I error rate (%)**	**Convergence (%)**
Unadjusted for centre-effects	1.00	4.7	100
Fixed-effects	1.01	5.1	99.8
Random-effects	1.00	4.8	99.4
GEE	1.00	5.1	100
Mantel-Haenszel	1.01	4.0	100

**Table 3:** Results from simulations based on MIST2 dataset (OR = 0.23)

	**Mean treatment effect**	**Power (%)**	**Convergence (%)**
Unadjusted for centre-effects	0.20	66.5	97.1
Fixed-effects	0.19	65.0	96.3
Random-effects	0.19	66.0	96.1
GEE	0.20	66.5	98.0
Mantel-Haenszel	0.22	50.3	95.9

When the OR for treatment was set to 0.23, all methods had very small amounts of bias in the estimated treatment effect. Convergence rates were similar for all methods. All methods had similar power, apart from Mantel-Haenszel which led to a reduction in power of approximately 16% compared to other techniques.

### Simulations based on varied parameters

#### 5 centres

Results can be seen in Additional file 1. All methods of analysis gave unbiased estimates of treatment, except when the OR was extremely low (OR = 0.25); in this case, all techniques were slightly biased. Type I error rates were close to the nominal value of 5% for each analysis method, except for Mantel-Haenszel which gave error rates which were too low in some scenarios with a sample size of 200 (range across 16 scenarios 3.8 to 5.2%). Power was comparable between different analysis methods across all scenarios. Convergence rates were 99.6% or greater across all analysis methods and scenarios.

#### 50 centres

**Figure 1:**
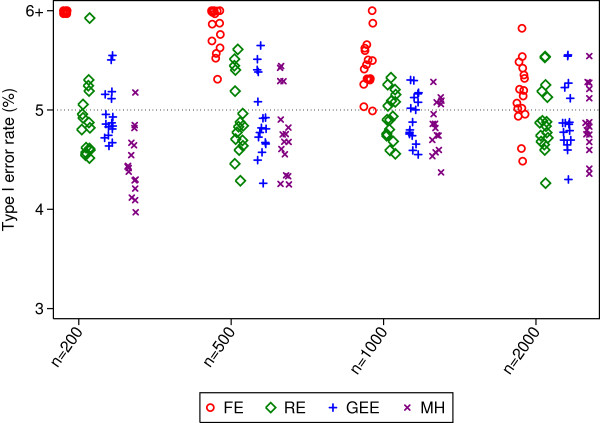
**Type I error rates for 50 centres.** This figure gives type I error rates from 16 different simulation scenarios for each sample size. Simulated scenarios involve different ICCs, event rates, randomisation methods, and distribution of patients across centres.

**Figure 2:**
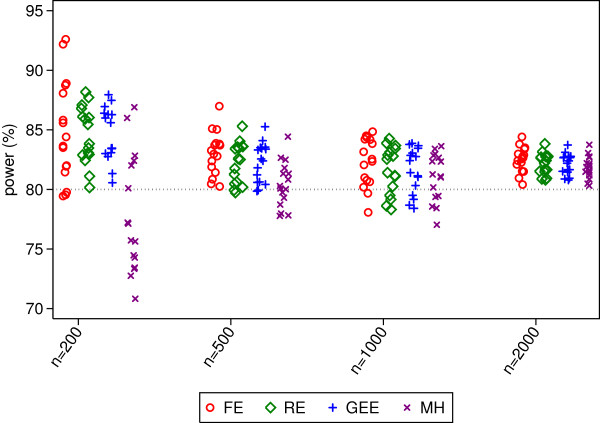
**Power rates for 50 centres.** This figure gives power from 16 different simulation scenarios for each sample size. Simulated scenarios involve different ICCs, event rates, randomisation methods, and distribution of patients across centres.

Results can be seen in Figures 1 and 2 and in Additional file 1. Fixed-effects gave severely biased estimates with only 200 patients, and gave slightly biased estimates with 500 patients. Other methods of analysis were all unbiased, except when the OR was extreme (OR = 0.25), when they all gave slightly biased estimates.

Type I error rates were inflated for fixed-effects in many (though not all) scenarios. This was most prominent with smaller sample sizes (range 6.8 to 9.6% with 200 patients). Mantel-Haenszel gave error rates which were too low in some scenarios with only 200 patients. Random-effects and GEE had close to nominal type I error rates in all scenarios.

Random-effects and GEE had comparable power across all scenarios, and had either similar or higher power than Mantel-Haenszel. The difference between random-effects and GEE vs. Mantel-Haenszel was most pronounced with a small number of patients, where MH lost a median of 8% and 2% power compared with the other methods for sample sizes of 200 and 500 respectively. Fixed-effects had the highest power of any analysis method in some scenarios, although this was likely a direct result of the inflated type I error rate. Conversely, it also lost power compared to other methods in some scenarios.

Convergence rates for random-effects, GEE, and Mantel-Haenszel were high across all scenarios (99.4% or higher for all methods). Fixed-effects had convergence issues in some scenarios, although convergence rates for all scenarios were above 94.5%.

#### 100 centres

**Figure 3:**
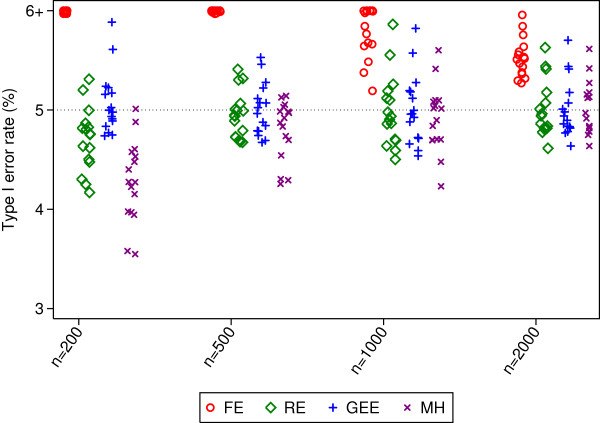
**Type I error rates for 100 centres.** This figure gives type I error rates from 16 different simulation scenarios for each sample size. Simulated scenarios involve different ICCs, event rates, randomisation methods, and distribution of patients across centres.

**Figure 4:**
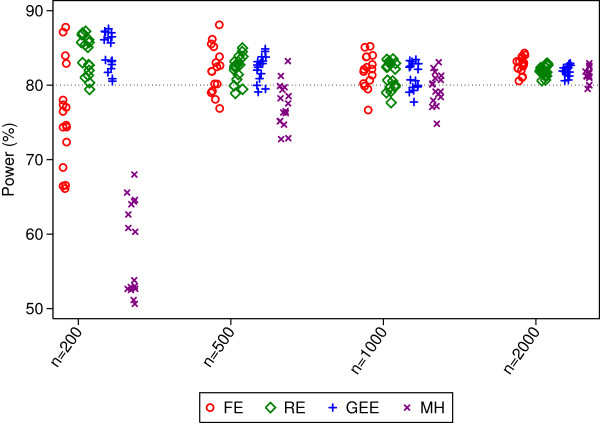
**Power rates for 100 centres.** This figure gives power from 16 different simulation scenarios for each sample size. Simulated scenarios involve different ICCs, event rates, randomisation methods, and distribution of patients across centres.

Results can be seen in Figures 3 and 4 and in Additional file 1. Fixed-effects led to substantially biased estimates with a small number of patients. As above, all other methods of analysis gave unbiased results apart from when the odds ratio was 0.25.

Type I error rates were inflated for fixed-effects in most scenarios (median 12.5, 7.0, 5.9, and 5.5% for sample sizes of 200, 500, 1000, and 2000 patients respectively). Mantel-Haenszel had type I error rates that were too low with a sample size of 200 patients (range 3.6 to 5.0). Random-effects and GEE had close to nominal type I error rates.

Random-effects and GEE had comparable power across all scenarios, and generally had higher power than Mantel-Haenszel (the median difference in power between MH and random-effects or GEE was 26, 6, and 2% for sample sizes of 200, 500, and 1000 respectively). Fixed-effects had either similar or lower power than random-effects or GEE.

Convergence rates for random-effects, GEE, and Mantel-Haenszel were high across all scenarios (99.4% or higher for all methods). Convergence rates for fixed-effects were above 91.4% for all scenarios.

### Sensitivity analysis – large ICC

Results for simulations using an ICC of 0.25 were similar to results from simulations using smaller ICCs. Random-effects and GEE gave close to nominal type I error rates in all scenarios, and had high power. The type I error rate for Mantel-Haenszel was too low when there were a small number of patients compared to the number of centres, and had lower power than other methods in these scenarios. Fixed-effects led to inflated type I error rates in a number of scenarios.

### Sensitivity analysis – GEE with a robust variance estimator

GEEs with robust SEs gave 100% convergence and was unbiased in all scenarios. With only 5 centres, the type I error rates were too large (range across four scenarios 11.9 to 12.6%). The type I error rates for 50 and 100 centres were slightly larger than nominal (range across four scenarios 5.0 to 5.8, and 4.9 to 5.9 for 50 and 100 centres respectively). Power was nominal across all scenarios (range across 12 scenarios 80.0 to 84.1%).

## Discussion

When patient outcomes vary substantially across centres, it can be useful to account for centre-effects in the analysis to increase power. We performed a large scale simulation study to investigate which methods of analysis performed well across a wide range of scenarios.

### Summary of results – MIST2

Based on re-analysis of the MIST2 trial, we found that both fixed-effects and Mantel-Haenszel dropped a substantial number of patients from the analysis, whereas random-effects and GEE allowed all patients to be included in the analysis. A simulation study based on this dataset showed that Mantel-Haenszel led to a large reduction in power; all other methods of analysis performed well. Despite the fact that GEE did not converge in the MIST2 re-analysis, convergence rates were high in the simulation study.

### Summary of results – simulation study

We found that fixed-effects lead to inflated type I error rates in most scenarios with either 50 or 100 centres. However, it gave nominal error rates and good power when there were only 5 centres. We therefore recommend that fixed-effects only be used with a small number of centres, and a large number of patients in each centre.

Mantel-Haenszel gave type I error rates that were too low in some scenarios with only 200 patients. It also led to a reduction in power compared to random-effects or GEE in many scenarios. In many cases the loss in power was partially caused by some centres being dropped from the MH analysis, either because all the patients in the centre were assigned to one treatment arm, or because all patients in a centre had the same outcome (either all successes or all failures). However, there was still a reduction in power even in scenarios where no centres would be dropped from the analysis.

Random-effects and GEE with non-robust SEs gave close to nominal type I error rates across all scenarios. They both gave similar power, and had either the same or higher power compared to Mantel-Haenszel in all scenarios. They also generally had the same or higher power than fixed-effects, apart from the scenarios where fixed-effects gained power due to the large inflation in the type I error rates.

Our sensitivity analysis found that GEE with robust SEs lead to severely inflated type I error rates with a small number of centres, and slightly inflated error rates even with 50 and 100 centres. Therefore, we do not recommend its use in the analysis of multicentre RCTs.

### Recommendations

#### When and why to account for centre-effects

When to account for centre-effects in the analysis depends on the trial design, specifically whether centre has been used as a stratification factor in the randomisation process. If centre has been stratified on, then we recommend it be included in the analysis to ensure correct p-values and confidence intervals, and to avoid a loss in power. If centre has not been stratified on, we recommend it be included in the analysis if the ICC is expected to be large, as this will increase power.

However, in both of the above scenarios, centre should only be included in the analysis if adjustment is unlikely to lead to convergence problems or unstable estimates.

#### How to account for centre-effects

We do not recommend the use of either Mantel-Haenszel or GEE with robust SEs, as both have been shown to perform poorly in many scenarios.

Fixed-effects can be used with a small number of centres, though should be avoided with a moderate or large number of centres. Random-effects and GEE with non-robust SEs should be used with a moderate or large number of centres. They can also be used with a small number of centres; however, their use has not been assessed with fewer than 5 centres, so fixed-effects may be preferable with only 2–3 centres.

### A warning against data-driven model selection

In some scenarios, it may be tempting to assess the model assumptions, or to test model fit before deciding on a final analysis model. For example, we could test whether the ICC > 0 and remove the centre-effects from the analysis if the result is non-significant. Likewise, when using a mixed-effects logistic regression model we could assess the normality of the random-effects, and use a different analysis method if the normality assumption is in doubt. Although both of these procedures may seem sensible, a large amount of research has showing that using trial data to choose the method of analysis can lead to biased results and incorrect type I error rates [21-24]. Furthermore, there is usually little to be lost by accounting for centre-effects when the ICC truly is 0, and most research has found that estimation and inference regarding the treatment effect in mixed-effects logistic regression models is robust to the misspecification of the distribution of the centre-effects [18,25]. Therefore, the method of analysis should be pre-specified in the protocol or statistical analysis plan, and should not be modified based assessment of the data.

### Limitations

Our study had several limitations. There are some methods of analysis that could potentially be used for multicentre trials with a binary endpoint that we did not consider, such as conditional logistic regression, propensity scores [26], or a permutation test approach [27,28]. Secondly, we generated data for the simulation study based on a random-effects model, which may have given an unfair advantage to random-effects models in the analysis. However, previous research has shown that, with a continuous outcome, random-effects outperformed fixed-effects even when the data were generated under a fixed-effects model [3]; therefore, it is unlikely that this is the sole reason random-effects performed so well. Finally, we have not considered the issue of treatment-by-centre interaction. We agree with the ICH E9 guidelines which state that treatment-by-centre interactions should not be addressed as part of the primary analysis [29], and have therefore focused on methods which reflect the primary analysis.

## Conclusion

Fixed-effects, random-effects, or GEE with non-robust SEs should be used with a small number of centres. With a moderate or large number of centres, we recommend the use of either random-effects or GEE with a non-robust SE.

## Abbreviations

GEE: Generalised estimating equation; ICC: Intraclass correlation coefficient; ICH: International conference on harmonisation; MH: Mantel-Haenszel; MIST2: The second multicentre intrapleural sepsis trial; OR: Odds ratio; RCT: Randomised controlled trial; SE: Standard error.

## Competing interests

The author declared that he has no competing interests.

## Pre-publication history

The pre-publication history for this paper can be accessed here:

http://www.biomedcentral.com/1471-2288/14/20/prepub

## Supplementary Material

Additional file 1 Additional methods and results. Click here for file
