# Supplementary material for: Accounting for centre-effects in multicentre trials with a binary outcome – when, why, and how?
Source: BMC Med Res Methodol. 2014 Feb 10;14:20. doi: 10.1186/1471-2288-14-20 (PMC3923100; doi:10.1186/1471-2288-14-20)
Supplement: Additional file 1 — Additional methods and results. [file 1471-2288-14-20-S1.docx]

**Accounting for centre-effects in multicentre trials with a binary outcome – when, why, and how?**

Brennan C Kahan

**1 - Centre sizes used for skewed patient distribution**

Each of the following tables gives the patients-per-centre *n* and the number of centres recruiting *n* patients for skewed patient distributions (each centre has the same number of patients in an even patient distribution).

For example, table 1 shows that with 5 centres and 200 patients, one centre recruits 10 patients, one recruits 25 patients, one recruits 40 patients, one recruits 55 patients, and one recruits 70 patients. Table 5 shows that with 50 centres and 200 patients, 13 centres recruit 1 patient, 12 centres recruit 2 patients, 10 centres recruit 3 patients, 7 centres recruit 4 patients, 5 centres recruit 10 patients, 2 centres recruit 15 patients, and 1 centre recruits 25 patients.

The numbers in these represent the centre-patient setup for simulations.

- 1. **- 5 centres**

**Table 1 - 5 centres, 200 patients**

| *n* | Centres |
| --- | --- |
| 10 | 1 |
| 25 | 1 |
| 40 | 1 |
| 55 | 1 |
| 70 | 1 |

**Table 2 - 5 centres, 500 patients**

| *n* | Centres |
| --- | --- |
| 50 | 1 |
| 75 | 1 |
| 100 | 1 |
| 125 | 1 |
| 150 | 1 |

**Table 3 - 5 centres, 1000 patients**

| *n* | Centres |
| --- | --- |
| 50 | 1 |
| 125 | 1 |
| 200 | 1 |
| 275 | 1 |
| 350 | 1 |

**Table 4 - 5 centres, 2000 patients**

| *n* | Centres |
| --- | --- |
| 100 | 1 |
| 300 | 1 |
| 400 | 1 |
| 500 | 1 |
| 700 | 1 |

- 1. **– 50 centres**

**Table 5 - 50 centres, 200 patients**

| *n* | Centres |
| --- | --- |
| 1 | 13 |
| 2 | 12 |
| 3 | 10 |
| 4 | 7 |
| 10 | 5 |
| 15 | 2 |
| 25 | 1 |

**Table 6 - 50 centres, 500 patients**

| *n* | Centres |
| --- | --- |
| 1 | 10 |
| 2 | 10 |
| 5 | 12 |
| 10 | 11 |
| 25 | 4 |
| 50 | 2 |
| 100 | 1 |

**Table 7 - 50 centres, 1000 patients**

| *n* | Centres |
| --- | --- |
| 1 | 10 |
| 2 | 10 |
| 5 | 10 |
| 10 | 7 |
| 25 | 4 |
| 50 | 4 |
| 75 | 2 |
| 100 | 2 |
| 200 | 1 |

**Table 8 - 50 centres, 2000 patients**

| *n* | Centres |
| --- | --- |
| 1 | 10 |
| 2 | 10 |
| 5 | 10 |
| 10 | 2 |
| 25 | 4 |
| 50 | 2 |
| 75 | 4 |
| 100 | 5 |
| 200 | 2 |
| 500 | 1 |

**1.3 – 100 centres**

**Table 9 - 100 centres, 200 patients**

| *n* | Centres |
| --- | --- |
| 1 | 54 |
| 2 | 28 |
| 3 | 10 |
| 4 | 5 |
| 10 | 2 |
| 20 | 1 |

**Table 10 - 100 centres, 500 patients**

| *n* | Centres |
| --- | --- |
| 1 | 33 |
| 2 | 26 |
| 3 | 15 |
| 4 | 10 |
| 10 | 8 |
| 20 | 5 |
| 50 | 3 |

**Table 11 - 100 centres, 1000 patients**

| *n* | Centres |
| --- | --- |
| 1 | 24 |
| 2 | 18 |
| 3 | 20 |
| 4 | 15 |
| 10 | 10 |
| 20 | 6 |
| 50 | 4 |
| 100 | 2 |
| 200 | 1 |

**Table 12 - 100 centres, 2000 patients**

| *n* | Centres |
| --- | --- |
| 1 | 20 |
| 2 | 15 |
| 3 | 12 |
| 4 | 16 |
| 10 | 15 |
| 20 | 10 |
| 50 | 6 |
| 100 | 3 |
| 200 | 2 |
| 500 | 1 |

**2 - Results from simulation study based on varied parameters**

***Summaries***

**Table 13 - Median (min/max) of estimated ORs from 16 simulated scenarios (true OR =1)***

| Centres | Sample size | Fixed-effects | Random-effects | GEE | Mantel-Haenszel |
| --- | --- | --- | --- | --- | --- |
| 5 | 200 | 1.00 (0.99 to 1.01) | 1.00 (0.99 to 1.01) | 1.00 (0.99 to 1.01) | 1.00 (0.99 to 1.01) |
|  | 500 | 1.00 (1.00 to 1.00) | 1.00 (1.00 to 1.00) | 1.00 (1.00 to 1.00) | 1.00 (1.00 to 1.00) |
|  | 1000 | 1.00 (1.00 to 1.01) | 1.00 (1.00 to 1.01) | 1.00 (1.00 to 1.01) | 1.00 (1.00 to 1.01) |
|  | 2000 | 1.00 (1.00 to 1.00) | 1.00 (1.00 to 1.00) | 1.00 (1.00 to 1.00) | 1.00 (1.00 to 1.00) |
| 50 | 200 | 1.00 (0.99 to 1.01) | 1.00 (0.99 to 1.01) | 1.00 (0.99 to 1.01) | 1.00 (0.99 to 1.01) |
|  | 500 | 1.00 (1.00 to 1.01) | 1.00 (1.00 to 1.01) | 1.00 (1.00 to 1.01) | 1.00 (1.00 to 1.01) |
|  | 1000 | 1.00 (0.99 to 1.00) | 1.00 (0.99 to 1.00) | 1.00 (0.99 to 1.00) | 1.00 (0.99 to 1.00) |
|  | 2000 | 1.00 (1.00 to 1.00) | 1.00 (1.00 to 1.00) | 1.00 (0.99 to 1.03) | 1.00 (1.00 to 1.00) |
| 100 | 200 | 1.00 (0.98 to 1.02) | 1.00 (0.99 to 1.01) | 1.00 (0.99 to 1.01) | 1.00 (0.99 to 1.01) |
|  | 500 | 1.00 (0.99 to 1.00) | 1.00 (0.99 to 1.00) | 1.00 (0.99 to 1.00) | 1.00 (0.99 to 1.00) |
|  | 1000 | 1.00 (1.00 to 1.00) | 1.00 (1.00 to 1.00) | 1.00 (1.00 to 1.00) | 1.00 (1.00 to 1.00) |
|  | 2000 | 1.00 (1.00 to 1.00) | 1.00 (1.00 to 1.00) | 1.00 (1.00 to 1.13) | 1.00 (1.0 to 1.00) |

* This figure gives the median (min/max) result from 16 different simulation scenarios for each centre/sample size combination. Simulated scenarios involve different ICCs, event rates, randomisation methods, and distribution of patients across centres.

**Table 14 - Median (min/max) of type I error rates from 16 simulated scenarios (true OR =1)***

| Centres | Sample size | Fixed-effects | Random-effects | GEE | Mantel-Haenszel |
| --- | --- | --- | --- | --- | --- |
| 5 | 200 | 5.1 (4.3 to 5.7) | 4.9 (4.1 to 5.7) | 4.8 (4.0 to 5.6) | 4.5 (3.8 to 5.2) |
|  | 500 | 5.0 (4.5 to 5.8) | 5.0 (4.4 to 5.6) | 4.9 (4.3 to 5.7) | 4.9 (4.3 to 5.7) |
|  | 1000 | 5.1 (4.3 to 6.0) | 5.0 (4.3 to 6.0) | 5.0 (4.2 to 6.0) | 5.0 (4.1 to 5.9) |
|  | 2000 | 4.8 (4.5 to 5.6) | 4.8 (4.5 to 5.6) | 4.7 (4.5 to 5.6) | 4.8 (4.5 to 5.6) |
| 50 | 200 | 7.6 (6.8 to 9.6) | 4.9 (4.5 to 6.0) | 4.9 (4.6 to 5.6) | 4.4 (4.0 to 5.2) |
|  | 500 | 5.9 (5.3 to 7.0) | 4.8 (4.3 to 5.6) | 4.8 (4.3 to 5.7) | 4.7 (4.2 to 5.5) |
|  | 1000 | 5.4 (5.0 to 6.0) | 4.9 (4.5 to 5.3) | 4.9 (4.5 to 5.3) | 4.8 (4.4 to 5.3) |
|  | 2000 | 5.1 (4.7 to 5.8) | 4.8 (4.2 to 5.5) | 4.8 (4.3 to 5.5) | 4.8 (4.3 to 5.5) |
| 100 | 200 | 12.5 (8.8 to 16.6) | 4.8 (4.2 to 5.3) | 5.0 (4.7 to 5.9) | 4.3 (3.6 to 5.0) |
|  | 500 | 7.0 (6.0 to 7.9) | 4.9 (4.7 to 5.4) | 5.0 (4.7 to 5.6) | 4.9 (4.2 to 5.1) |
|  | 1000 | 5.9 (5.2 to 6.8) | 4.9 (4.5 to 5.8) | 5.0 (4.5 to 5.8) | 4.9 (4.2 to 5.6) |
|  | 2000 | 5.5 (5.3 to 5.9) | 4.9 (4.6 to 5.6) | 4.9 (4.6 to 5.7) | 4.9 (4.6 to 5.6) |

* This figure gives the median (min/max) result from 16 different simulation scenarios for each centre/sample size combination. Simulated scenarios involve different ICCs, event rates, randomisation methods, and distribution of patients across centres.

**Table 15 - Median (min/max) of convergence rates from 16 simulated scenarios (true OR =1)***

| Centres | Sample size | Fixed-effects | Random-effects | GEE | Mantel-Haenszel |
| --- | --- | --- | --- | --- | --- |
| 5 | 200 | 100 (99.9 to 100) | 99.9 (99.8 to 100) | 100 (100 to 100) | 100 (99.8 to 100) |
|  | 500 | 100 (100 to 100) | 99.9 (99.7 to 100) | 100 (100 to 100) | 100 (99.9 to 100) |
|  | 1000 | 100 (100 to 100) | 99.9 (99.6 to 100) | 100 (100 to 100) | 100 (100 to 100) |
|  | 2000 | 100 (100 to 100) | 99.9 (99.7 to 100) | 100 (99.8 to 100) | 100 (100 to 100) |
| 50 | 200 | 100 (100 to 100) | 99.9 (99.7 to 100) | 100 (100 to 100) | 100 (99.8 to 100) |
|  | 500 | 100 (99.3 to 100) | 100 (99.8 to 100) | 100 (100 to 100) | 100 (100 to 100) |
|  | 1000 | 100 (98.4 to 100) | 99.9 (99.4 to 100) | 100 (100 to 100) | 100 (99.9 to 100) |
|  | 2000 | 100 (94.8 to 100) | 99.9 (99.8 to 100) | 100 (99.9 to 100) | 100 (100 to 100) |
| 100 | 200 | 99.9 (99.1 to 100) | 99.9 (99.8 to 100) | 100 (100 to 100) | 100 (99.9 to 100) |
|  | 500 | 100 (99.8 to 100) | 100 (99.8 to 100) | 100 (100 to 100) | 100 (100 to 100) |
|  | 1000 | 100 (98.9 to 100) | 100 (99.8 to 100) | 100 (100 to 100) | 100 (100 to 100) |
|  | 2000 | 100 (97.7 to 100) | 100 (99.4 to 100) | 100 (99.9 to 100) | 100 (100 to 100) |

* This figure gives the median (min/max) result from 16 different simulation scenarios for each centre/sample size combination. Simulated scenarios involve different ICCs, event rates, randomisation methods, and distribution of patients across centres.

**Table 16 - Median (min/max) of estimated ORs from 8 simulated scenarios (true OR <1)***

| Centres | Sample size | Event rate in control arm | True OR | Fixed-effects | Random-effects | GEE | Mantel-Haenszel |
| --- | --- | --- | --- | --- | --- | --- | --- |
| 5 | 200 | 0.2 | 0.25 | 0.22 (0.22 to 0.23) | 0.22 (0.21 to 0.22) | 0.22 (0.20 to 0.23) | 0.23 (0.23 to 0.23) |
|  | 200 | 0.5 | 0.42 | 0.41 (0.40 to 0.41) | 0.42 (0.41 to 0.42) | 0.43 (0.42 to 0.43) | 0.41 (0.41 to 0.42) |
|  | 500 | 0.2 | 0.47 | 0.47 (0.46 to 0.47) | 0.47 (0.47 to 0.47) | 0.47 (0.47 to 0.48) | 0.47 (0.47 to 0.47) |
|  | 500 | 0.5 | 0.59 | 0.58 (0.58 to 0.59) | 0.59 (0.59 to 0.59) | 0.60 (0.59 to 0.60) | 0.59 (0.59 to 0.59) |
|  | 1000 | 0.2 | 0.61 | 0.61 (0.60 to 0.61) | 0.61 (0.61 to 0.61) | 0.61 (0.61 to 0.62) | 0.61 (0.61 to 0.61) |
|  | 1000 | 0.5 | 0.70 | 0.69 (0.69 to 0.70) | 0.69 (0.69 to 0.70) | 0.70 (0.70 to 0.71) | 0.69 (0.69 to 0.70) |
|  | 2000 | 0.2 | 0.71 | 0.71 (0.71 to 0.71) | 0.71 (0.71 to 0.71) | 0.71 (0.71 to 0.72) | 0.71 (0.71 to 0.71) |
|  | 2000 | 0.5 | 0.77 | 0.77 (0.77 to 0.77) | 0.77 (0.77 to 0.77) | 0.77 (0.77 to 0.78) | 0.77 (0.77 to 0.77) |
| 50 | 200 | 0.2 | 0.25 | 0.15 (0.13 to 0.17) | 0.22 (0.22 to 0.23) | 0.23 (0.22 to 0.24) | 0.23 (0.22 to 0.23) |
|  | 200 | 0.5 | 0.42 | 0.32 (0.30 to 0.35) | 0.41 (0.41 to 0.42) | 0.43 (0.42 to 0.44) | 0.41 (0.41 to 0.41) |
|  | 500 | 0.2 | 0.47 | 0.44 (0.43 to 0.45) | 0.47 (0.47 to 0.47) | 0.48 (0.47 to 0.48) | 0.47 (0.47 to 0.47) |
|  | 500 | 0.5 | 0.59 | 0.56 (0.56 to 0.57) | 0.59 (0.59 to 0.59) | 0.60 (0.59 to 0.61) | 0.59 (0.59 to 0.59) |
|  | 1000 | 0.2 | 0.61 | 0.59 (0.59 to 0.60) | 0.61 (0.60 to 0.61) | 0.61 (0.61 to 0.62) | 0.61 (0.60 to 0.61) |
|  | 1000 | 0.5 | 0.70 | 0.68 (0.68 to 0.69) | 0.69 (0.69 to 0.70) | 0.70 (0.70 to 0.71) | 0.69 (0.69 to 0.70) |
|  | 2000 | 0.2 | 0.71 | 0.70 (0.70 to 0.71) | 0.71 (0.71 to 0.71) | 0.72 (0.71 to 0.75) | 0.71 (0.71 to 0.71) |
|  | 2000 | 0.5 | 0.77 | 0.77 (0.76 to 0.77) | 0.77 (0.77 to 0.77) | 0.77 (0.77 to 0.78) | 0.77 (0.77 to 0.77) |
| 1000 | 200 | 0.2 | 0.25 | 0.09 (0.05 to 0.13) | 0.22 (0.21 to 0.22) | 0.24 (0.23 to 0.24) | 0.23 (0.22 to 0.25) |
|  | 200 | 0.5 | 0.42 | 0.23 (0.16 to 0.29) | 0.41 (0.40 to 0.41) | 0.43 (0.42 to 0.44) | 0.41 (0.40 to 0.41) |
|  | 500 | 0.2 | 0.47 | 0.40 (0.39 to 0.43) | 0.47 (0.47 to 0.47) | 0.48 (0.47 to 0.49) | 0.47 (0.47 to 0.47) |
|  | 500 | 0.5 | 0.59 | 0.53 (0.51 to 0.55) | 0.59 (0.59 to 0.59) | 0.60 (0.59 to 0.61) | 0.59 (0.59 to 0.59) |
|  | 1000 | 0.2 | 0.61 | 0.58 (0.57 to 0.59) | 0.61 (0.60 to 0.61) | 0.61 (0.61 to 0.62) | 0.61 (0.60 to 0.61) |
|  | 1000 | 0.5 | 0.70 | 0.67 (0.66 to 0.68) | 0.69 (0.69 to 0.70) | 0.70 (0.70 to 0.71) | 0.69 (0.69 to 0.70) |
|  | 2000 | 0.2 | 0.71 | 0.70 (0.70 to 0.70) | 0.71 (0.71 to 0.71) | 0.72 (0.71 to 0.84) | 0.71 (0.71 to 0.71) |
|  | 2000 | 0.5 | 0.77 | 0.76 (0.76 to 0.77) | 0.77 (0.77 to 0.77) | 0.77 (0.77 to 0.78) | 0.77 (0.77 to 0.77) |

* This figure gives the median (min/max) result from 8 different simulation scenarios for each centre/sample size combination. Simulated scenarios involve different ICCs, randomisation methods, and distribution of patients across centres.

**Table 17 - Median (min/max) of power from 16 simulated scenarios (true OR <1)***

| Centres | Sample size | Fixed-effects | Random-effects | GEE | Mantel-Haenszel |
| --- | --- | --- | --- | --- | --- |
| 5 | 200 | 85.5 (82.0 to 87.4) | 85.1 (81.5 to 86.8) | 84.8 (81.4 to 86.6) | 84.7 (81.0 to 86.9) |
|  | 500 | 83.4 (80.2 to 84.2) | 83.2 (80.0 to 84.1) | 82.9 (80.0 to 84.0) | 83.0 (79.9 to 83.9) |
|  | 1000 | 82.0 (78.3 to 83.4) | 81.8 (78.2 to 83.2) | 81.8 (78.2 to 83.4) | 81.8 (78.2 to 83.1) |
|  | 2000 | 82.3 (80.6 to 83.1) | 82.2 (80.6 to 83.1) | 82.1 (80.6 to 83.1) | 82.1 (80.4 to 83.1) |
| 50 | 200 | 84.1 (79.1 to 92.6) | 84.8 (80.1 to 87.9) | 84.7 (80.5 to 87.6) | 76.6 (70.7 to 86.9) |
|  | 500 | 83.1 (80.3 to 86.9) | 82.5 (79.7 to 85.2) | 82.5 (79.8 to 85.2) | 80.2 (77.7 to 84.3) |
|  | 1000 | 82.4 (78.4 to 84.9) | 81.9 (78.6 to 84.3) | 81.8 (78.7 to 83.9) | 81.2 (77.3 to 83.7) |
|  | 2000 | 82.5 (80.3 to 84.2) | 82.3 (80.5 to 83.6) | 82.3 (80.5 to 83.5) | 81.8 (80.1 to 83.6) |
| 100 | 200 | 75.9 (65.9 to 87.7) | 84.2 (79.3 to 87.3) | 84.8 (80.4 to 87.5) | 57.3 (50.8 to 67.9) |
|  | 500 | 82.1 (76.9 to 87.9) | 82.8 (78.8 to 84.7) | 82.7 (79.0 to 84.6) | 77.0 (72.7 to 83.1) |
|  | 1000 | 82.1 (77.1 to 85.4) | 81.2 (78.1 to 83.5) | 81.2 (78.2 to 83.3) | 79.7 (75.3 to 82.9) |
|  | 2000 | 82.6 (80.0 to 84.1) | 82.0 (80.1 to 82.7) | 82.0 (80.1 to 82.7) | 81.4 (78.9 to 82.7) |

* This figure gives the median (min/max) result from 16 different simulation scenarios for each centre/sample size combination. Simulated scenarios involve different ICCs, event rates, randomisation methods, and distribution of patients across centres.

**Table 18 - Median (min/max) of convergence rates from 16 simulated scenarios (true OR <1)***

| Centres | Sample size | Fixed-effects | Random-effects | GEE | Mantel-Haenszel |
| --- | --- | --- | --- | --- | --- |
| 5 | 200 | 100 (99.2 to 100) | 99.9 (99.8 to 100) | 100 (99.9 to 100) | 100 (100 to 100) |
|  | 500 | 100 (100 to 100) | 99.9 (99.8 to 100) | 100 (100 to 100) | 100 (100 to 100) |
|  | 1000 | 100 (100 to 100) | 99.9 (99.7 to 100) | 100 (100 to 100) | 100 (100 to 100) |
|  | 2000 | 100 (100 to 100) | 99.9 (99.8 to 100) | 100 (99.8 to 100) | 100 (100 to 100) |
| 50 | 200 | 99.7 (98.7 to 100) | 99.9 (99.8 to 100) | 100 (99.9 to 100) | 100 (100 to 100) |
|  | 500 | 99.9 (97.4 to 100) | 99.9 (99.8 to 100) | 100 (100 to 100) | 100 (100 to 100) |
|  | 1000 | 99.9 (95.6 to 100) | 99.9 (99.7 to 100) | 100 (100 to 100) | 100 (100 to 100) |
|  | 2000 | 100 (94.5 to 100) | 100 (99.8 to 100) | 100 (99.9 to 100) | 100 (100 to 100) |
| 100 | 200 | 97.3 (91.4 to 100) | 99.9 (99.6 to 100) | 100 (99.9 to 100) | 100 (100 to 100) |
|  | 500 | 100 (99.8 to 100) | 100 (99.9 to 100) | 100 (100 to 100) | 100 (100 to 100) |
|  | 1000 | 99.9 (98.0 to 100) | 100 (99.8 to 100) | 100 (100 to 100) | 100 (100 to 100) |
|  | 2000 | 99.9 (97.7 to 100) | 99.9 (99.7 to 100) | 100 (99.8 to 100) | 100 (100 to 100) |

* This figure gives the median (min/max) result from 16 different simulation scenarios for each centre/sample size combination. Simulated scenarios involve different ICCs, event rates, randomisation methods, and distribution of patients across centres.

**3 - Results from simulation study based on varied parameters**

***Results from individual scenarios***

**Table 19 – Mean treatment effect – 5 centres, true OR=1**

| Sample size | Event rate | ICC | Patient distribution | Block size | True OR | Fixed effects | Random effects | GEE | MH |
| --- | --- | --- | --- | --- | --- | --- | --- | --- | --- |
| 200 | 0.2 | 0.025 | Even | 4 | 1 | 1.00 | 1.00 | 1.00 | 1.00 |
| 200 | 0.2 | 0.025 | Even | 20 | 1 | 1.00 | 1.00 | 1.00 | 1.00 |
| 200 | 0.2 | 0.025 | Skewed | 4 | 1 | 0.99 | 0.99 | 0.99 | 0.99 |
| 200 | 0.2 | 0.025 | Skewed | 20 | 1 | 1.01 | 1.01 | 1.01 | 1.01 |
| 200 | 0.2 | 0.075 | Even | 4 | 1 | 1.00 | 1.00 | 1.00 | 1.00 |
| 200 | 0.2 | 0.075 | Even | 20 | 1 | 0.99 | 0.99 | 0.99 | 0.99 |
| 200 | 0.2 | 0.075 | Skewed | 4 | 1 | 1.01 | 1.01 | 1.01 | 1.01 |
| 200 | 0.2 | 0.075 | Skewed | 20 | 1 | 1.00 | 1.00 | 1.00 | 1.00 |
| 200 | 0.5 | 0.025 | Even | 4 | 1 | 1.00 | 1.00 | 1.00 | 1.00 |
| 200 | 0.5 | 0.025 | Even | 20 | 1 | 0.99 | 0.99 | 0.99 | 0.99 |
| 200 | 0.5 | 0.025 | Skewed | 4 | 1 | 1.00 | 1.00 | 1.00 | 1.00 |
| 200 | 0.5 | 0.025 | Skewed | 20 | 1 | 1.00 | 1.00 | 1.00 | 1.00 |
| 200 | 0.5 | 0.075 | Even | 4 | 1 | 0.99 | 0.99 | 0.99 | 0.99 |
| 200 | 0.5 | 0.075 | Even | 20 | 1 | 1.00 | 1.00 | 1.00 | 1.00 |
| 200 | 0.5 | 0.075 | Skewed | 4 | 1 | 1.00 | 1.00 | 1.00 | 1.00 |
| 200 | 0.5 | 0.075 | Skewed | 20 | 1 | 1.00 | 1.00 | 1.00 | 1.00 |
| 500 | 0.2 | 0.025 | Even | 4 | 1 | 1.00 | 1.00 | 1.00 | 1.00 |
| 500 | 0.2 | 0.025 | Even | 20 | 1 | 1.00 | 1.00 | 1.00 | 1.00 |
| 500 | 0.2 | 0.025 | Skewed | 4 | 1 | 1.00 | 1.00 | 1.00 | 1.00 |
| 500 | 0.2 | 0.025 | Skewed | 20 | 1 | 1.00 | 1.00 | 1.00 | 1.00 |
| 500 | 0.2 | 0.075 | Even | 4 | 1 | 1.00 | 1.00 | 1.00 | 1.00 |
| 500 | 0.2 | 0.075 | Even | 20 | 1 | 1.00 | 1.00 | 1.00 | 1.00 |
| 500 | 0.2 | 0.075 | Skewed | 4 | 1 | 1.00 | 1.00 | 1.00 | 1.00 |
| 500 | 0.2 | 0.075 | Skewed | 20 | 1 | 1.00 | 1.00 | 1.00 | 1.00 |
| 500 | 0.5 | 0.025 | Even | 4 | 1 | 1.00 | 1.00 | 1.00 | 1.00 |
| 500 | 0.5 | 0.025 | Even | 20 | 1 | 1.00 | 1.00 | 1.00 | 1.00 |
| 500 | 0.5 | 0.025 | Skewed | 4 | 1 | 1.00 | 1.00 | 1.00 | 1.00 |
| 500 | 0.5 | 0.025 | Skewed | 20 | 1 | 1.00 | 1.00 | 1.00 | 1.00 |
| 500 | 0.5 | 0.075 | Even | 4 | 1 | 1.00 | 1.00 | 1.00 | 1.00 |
| 500 | 0.5 | 0.075 | Even | 20 | 1 | 1.00 | 1.00 | 1.00 | 1.00 |
| 500 | 0.5 | 0.075 | Skewed | 4 | 1 | 1.00 | 1.00 | 1.00 | 1.00 |
| 500 | 0.5 | 0.075 | Skewed | 20 | 1 | 1.00 | 1.00 | 1.00 | 1.00 |
| 1000 | 0.2 | 0.025 | Even | 4 | 1 | 1.00 | 1.00 | 1.00 | 1.00 |
| 1000 | 0.2 | 0.025 | Even | 20 | 1 | 1.00 | 1.00 | 1.00 | 1.00 |
| 1000 | 0.2 | 0.025 | Skewed | 4 | 1 | 1.00 | 1.00 | 1.00 | 1.00 |
| 1000 | 0.2 | 0.025 | Skewed | 20 | 1 | 1.00 | 1.00 | 1.00 | 1.00 |
| 1000 | 0.2 | 0.075 | Even | 4 | 1 | 1.01 | 1.01 | 1.01 | 1.01 |
| 1000 | 0.2 | 0.075 | Even | 20 | 1 | 1.00 | 1.00 | 1.00 | 1.00 |
| 1000 | 0.2 | 0.075 | Skewed | 4 | 1 | 1.00 | 1.00 | 1.00 | 1.00 |
| 1000 | 0.2 | 0.075 | Skewed | 20 | 1 | 1.00 | 1.00 | 1.00 | 1.00 |
| 1000 | 0.5 | 0.025 | Even | 4 | 1 | 1.00 | 1.00 | 1.00 | 1.00 |
| 1000 | 0.5 | 0.025 | Even | 20 | 1 | 1.00 | 1.00 | 1.00 | 1.00 |
| 1000 | 0.5 | 0.025 | Skewed | 4 | 1 | 1.00 | 1.00 | 1.00 | 1.00 |
| 1000 | 0.5 | 0.025 | Skewed | 20 | 1 | 1.00 | 1.00 | 1.00 | 1.00 |
| 1000 | 0.5 | 0.075 | Even | 4 | 1 | 1.00 | 1.00 | 1.00 | 1.00 |
| 1000 | 0.5 | 0.075 | Even | 20 | 1 | 1.00 | 1.00 | 1.00 | 1.00 |
| 1000 | 0.5 | 0.075 | Skewed | 4 | 1 | 1.00 | 1.00 | 1.00 | 1.00 |
| 1000 | 0.5 | 0.075 | Skewed | 20 | 1 | 1.00 | 1.00 | 1.00 | 1.00 |
| 2000 | 0.2 | 0.025 | Even | 4 | 1 | 1.00 | 1.00 | 1.00 | 1.00 |
| 2000 | 0.2 | 0.025 | Even | 20 | 1 | 1.00 | 1.00 | 1.00 | 1.00 |
| 2000 | 0.2 | 0.025 | Skewed | 4 | 1 | 1.00 | 1.00 | 1.00 | 1.00 |
| 2000 | 0.2 | 0.025 | Skewed | 20 | 1 | 1.00 | 1.00 | 1.00 | 1.00 |
| 2000 | 0.2 | 0.075 | Even | 4 | 1 | 1.00 | 1.00 | 1.00 | 1.00 |
| 2000 | 0.2 | 0.075 | Even | 20 | 1 | 1.00 | 1.00 | 1.00 | 1.00 |
| 2000 | 0.2 | 0.075 | Skewed | 4 | 1 | 1.00 | 1.00 | 1.00 | 1.00 |
| 2000 | 0.2 | 0.075 | Skewed | 20 | 1 | 1.00 | 1.00 | 1.00 | 1.00 |
| 2000 | 0.5 | 0.025 | Even | 4 | 1 | 1.00 | 1.00 | 1.00 | 1.00 |
| 2000 | 0.5 | 0.025 | Even | 20 | 1 | 1.00 | 1.00 | 1.00 | 1.00 |
| 2000 | 0.5 | 0.025 | Skewed | 4 | 1 | 1.00 | 1.00 | 1.00 | 1.00 |
| 2000 | 0.5 | 0.025 | Skewed | 20 | 1 | 1.00 | 1.00 | 1.00 | 1.00 |
| 2000 | 0.5 | 0.075 | Even | 4 | 1 | 1.00 | 1.00 | 1.00 | 1.00 |
| 2000 | 0.5 | 0.075 | Even | 20 | 1 | 1.00 | 1.00 | 1.00 | 1.00 |
| 2000 | 0.5 | 0.075 | Skewed | 4 | 1 | 1.00 | 1.00 | 1.00 | 1.00 |
| 2000 | 0.5 | 0.075 | Skewed | 20 | 1 | 1.00 | 1.00 | 1.00 | 1.00 |

**Table 20 – Type I error rates - 5 centres, true OR=1**

| Sample size | Event rate | ICC | Patient distribution | Block size | True OR | Fixed effects | Random effects | GEE | MH |
| --- | --- | --- | --- | --- | --- | --- | --- | --- | --- |
| 200 | 0.2 | 0.025 | Even | 4 | 1 | 5.16 | 4.90 | 4.80 | 4.56 |
| 200 | 0.2 | 0.025 | Even | 20 | 1 | 4.92 | 4.81 | 4.70 | 4.53 |
| 200 | 0.2 | 0.025 | Skewed | 4 | 1 | 4.96 | 4.76 | 4.68 | 4.54 |
| 200 | 0.2 | 0.025 | Skewed | 20 | 1 | 5.32 | 5.02 | 4.94 | 4.78 |
| 200 | 0.2 | 0.075 | Even | 4 | 1 | 5.37 | 5.17 | 4.98 | 5.00 |
| 200 | 0.2 | 0.075 | Even | 20 | 1 | 5.27 | 4.87 | 4.72 | 4.65 |
| 200 | 0.2 | 0.075 | Skewed | 4 | 1 | 5.04 | 4.61 | 4.46 | 4.54 |
| 200 | 0.2 | 0.075 | Skewed | 20 | 1 | 4.27 | 4.07 | 4.02 | 3.80 |
| 200 | 0.5 | 0.025 | Even | 4 | 1 | 5.70 | 5.66 | 5.60 | 5.16 |
| 200 | 0.5 | 0.025 | Even | 20 | 1 | 5.22 | 5.23 | 5.16 | 4.85 |
| 200 | 0.5 | 0.025 | Skewed | 4 | 1 | 5.68 | 5.59 | 5.56 | 4.82 |
| 200 | 0.5 | 0.025 | Skewed | 20 | 1 | 5.06 | 4.89 | 4.80 | 4.44 |
| 200 | 0.5 | 0.075 | Even | 4 | 1 | 4.82 | 4.75 | 4.78 | 4.50 |
| 200 | 0.5 | 0.075 | Even | 20 | 1 | 4.88 | 4.72 | 4.72 | 4.50 |
| 200 | 0.5 | 0.075 | Skewed | 4 | 1 | 4.98 | 4.85 | 4.88 | 4.40 |
| 200 | 0.5 | 0.075 | Skewed | 20 | 1 | 4.56 | 4.61 | 4.56 | 4.12 |
| 500 | 0.2 | 0.025 | Even | 4 | 1 | 4.98 | 4.94 | 4.92 | 4.86 |
| 500 | 0.2 | 0.025 | Even | 20 | 1 | 5.24 | 5.20 | 5.14 | 5.12 |
| 500 | 0.2 | 0.025 | Skewed | 4 | 1 | 4.92 | 4.82 | 4.72 | 4.72 |
| 500 | 0.2 | 0.025 | Skewed | 20 | 1 | 4.64 | 4.56 | 4.52 | 4.46 |
| 500 | 0.2 | 0.075 | Even | 4 | 1 | 4.52 | 4.42 | 4.30 | 4.34 |
| 500 | 0.2 | 0.075 | Even | 20 | 1 | 4.54 | 4.50 | 4.42 | 4.46 |
| 500 | 0.2 | 0.075 | Skewed | 4 | 1 | 4.56 | 4.54 | 4.44 | 4.48 |
| 500 | 0.2 | 0.075 | Skewed | 20 | 1 | 5.02 | 4.94 | 4.70 | 4.86 |
| 500 | 0.5 | 0.025 | Even | 4 | 1 | 4.82 | 4.80 | 4.80 | 4.68 |
| 500 | 0.5 | 0.025 | Even | 20 | 1 | 5.32 | 5.28 | 5.26 | 5.18 |
| 500 | 0.5 | 0.025 | Skewed | 4 | 1 | 5.36 | 5.43 | 5.38 | 5.08 |
| 500 | 0.5 | 0.025 | Skewed | 20 | 1 | 5.36 | 5.33 | 5.32 | 5.14 |
| 500 | 0.5 | 0.075 | Even | 4 | 1 | 5.08 | 5.01 | 5.04 | 4.98 |
| 500 | 0.5 | 0.075 | Even | 20 | 1 | 4.86 | 4.76 | 4.78 | 4.70 |
| 500 | 0.5 | 0.075 | Skewed | 4 | 1 | 5.48 | 5.39 | 5.44 | 5.32 |
| 500 | 0.5 | 0.075 | Skewed | 20 | 1 | 5.82 | 5.61 | 5.68 | 5.56 |
| 1000 | 0.2 | 0.025 | Even | 4 | 1 | 4.96 | 4.96 | 4.92 | 4.92 |
| 1000 | 0.2 | 0.025 | Even | 20 | 1 | 4.66 | 4.62 | 4.56 | 4.58 |
| 1000 | 0.2 | 0.025 | Skewed | 4 | 1 | 5.18 | 5.16 | 5.10 | 5.10 |
| 1000 | 0.2 | 0.025 | Skewed | 20 | 1 | 4.88 | 4.82 | 4.80 | 4.76 |
| 1000 | 0.2 | 0.075 | Even | 4 | 1 | 4.76 | 4.70 | 4.54 | 4.60 |
| 1000 | 0.2 | 0.075 | Even | 20 | 1 | 4.86 | 4.74 | 4.52 | 4.66 |
| 1000 | 0.2 | 0.075 | Skewed | 4 | 1 | 5.38 | 5.35 | 5.18 | 5.28 |
| 1000 | 0.2 | 0.075 | Skewed | 20 | 1 | 4.98 | 4.94 | 4.82 | 4.88 |
| 1000 | 0.5 | 0.025 | Even | 4 | 1 | 5.98 | 5.98 | 5.96 | 5.86 |
| 1000 | 0.5 | 0.025 | Even | 20 | 1 | 4.70 | 4.64 | 4.66 | 4.58 |
| 1000 | 0.5 | 0.025 | Skewed | 4 | 1 | 5.14 | 5.11 | 5.12 | 5.02 |
| 1000 | 0.5 | 0.025 | Skewed | 20 | 1 | 5.24 | 5.17 | 5.18 | 5.02 |
| 1000 | 0.5 | 0.075 | Even | 4 | 1 | 5.48 | 5.47 | 5.46 | 5.42 |
| 1000 | 0.5 | 0.075 | Even | 20 | 1 | 5.50 | 5.47 | 5.46 | 5.44 |
| 1000 | 0.5 | 0.075 | Skewed | 4 | 1 | 5.32 | 5.30 | 5.28 | 5.26 |
| 1000 | 0.5 | 0.075 | Skewed | 20 | 1 | 4.26 | 4.25 | 4.24 | 4.14 |
| 2000 | 0.2 | 0.025 | Even | 4 | 1 | 4.98 | 4.90 | 4.88 | 4.88 |
| 2000 | 0.2 | 0.025 | Even | 20 | 1 | 4.54 | 4.54 | 4.50 | 4.54 |
| 2000 | 0.2 | 0.025 | Skewed | 4 | 1 | 4.80 | 4.76 | 4.76 | 4.74 |
| 2000 | 0.2 | 0.025 | Skewed | 20 | 1 | 4.58 | 4.52 | 4.52 | 4.52 |
| 2000 | 0.2 | 0.075 | Even | 4 | 1 | 4.80 | 4.78 | 4.64 | 4.76 |
| 2000 | 0.2 | 0.075 | Even | 20 | 1 | 4.62 | 4.61 | 4.50 | 4.58 |
| 2000 | 0.2 | 0.075 | Skewed | 4 | 1 | 4.62 | 4.61 | 4.61 | 4.58 |
| 2000 | 0.2 | 0.075 | Skewed | 20 | 1 | 4.64 | 4.60 | 4.60 | 4.58 |
| 2000 | 0.5 | 0.025 | Even | 4 | 1 | 4.52 | 4.51 | 4.50 | 4.50 |
| 2000 | 0.5 | 0.025 | Even | 20 | 1 | 5.02 | 5.03 | 5.02 | 5.02 |
| 2000 | 0.5 | 0.025 | Skewed | 4 | 1 | 5.06 | 5.05 | 5.05 | 5.04 |
| 2000 | 0.5 | 0.025 | Skewed | 20 | 1 | 5.58 | 5.59 | 5.59 | 5.58 |
| 2000 | 0.5 | 0.075 | Even | 4 | 1 | 5.26 | 5.27 | 5.26 | 5.24 |
| 2000 | 0.5 | 0.075 | Even | 20 | 1 | 4.88 | 4.88 | 4.88 | 4.86 |
| 2000 | 0.5 | 0.075 | Skewed | 4 | 1 | 4.56 | 4.53 | 4.53 | 4.52 |
| 2000 | 0.5 | 0.075 | Skewed | 20 | 1 | 4.82 | 4.83 | 4.83 | 4.78 |

**Table 21 – Convergence - 5 centres, true OR=1**

| Sample size | Event rate | ICC | Patient distribution | Block size | True OR | Fixed effects | Random effects | GEE | MH |
| --- | --- | --- | --- | --- | --- | --- | --- | --- | --- |
| 200 | 0.2 | 0.025 | Even | 4 | 1 | 99.96 | 99.96 | 100.00 | 99.92 |
| 200 | 0.2 | 0.025 | Even | 20 | 1 | 99.98 | 99.88 | 100.00 | 99.88 |
| 200 | 0.2 | 0.025 | Skewed | 4 | 1 | 100.00 | 99.94 | 100.00 | 100.00 |
| 200 | 0.2 | 0.025 | Skewed | 20 | 1 | 99.92 | 99.92 | 100.00 | 100.00 |
| 200 | 0.2 | 0.075 | Even | 4 | 1 | 99.86 | 99.78 | 100.00 | 99.92 |
| 200 | 0.2 | 0.075 | Even | 20 | 1 | 99.86 | 99.88 | 100.00 | 99.86 |
| 200 | 0.2 | 0.075 | Skewed | 4 | 1 | 99.96 | 99.86 | 100.00 | 100.00 |
| 200 | 0.2 | 0.075 | Skewed | 20 | 1 | 99.88 | 99.82 | 100.00 | 100.00 |
| 200 | 0.5 | 0.025 | Even | 4 | 1 | 100.00 | 99.94 | 100.00 | 99.94 |
| 200 | 0.5 | 0.025 | Even | 20 | 1 | 100.00 | 99.90 | 100.00 | 99.84 |
| 200 | 0.5 | 0.025 | Skewed | 4 | 1 | 100.00 | 99.88 | 100.00 | 100.00 |
| 200 | 0.5 | 0.025 | Skewed | 20 | 1 | 100.00 | 99.86 | 100.00 | 100.00 |
| 200 | 0.5 | 0.075 | Even | 4 | 1 | 100.00 | 99.86 | 100.00 | 99.96 |
| 200 | 0.5 | 0.075 | Even | 20 | 1 | 100.00 | 99.90 | 100.00 | 99.96 |
| 200 | 0.5 | 0.075 | Skewed | 4 | 1 | 100.00 | 99.86 | 100.00 | 100.00 |
| 200 | 0.5 | 0.075 | Skewed | 20 | 1 | 100.00 | 99.88 | 100.00 | 100.00 |
| 500 | 0.2 | 0.025 | Even | 4 | 1 | 100.00 | 99.96 | 100.00 | 99.98 |
| 500 | 0.2 | 0.025 | Even | 20 | 1 | 100.00 | 99.92 | 100.00 | 99.98 |
| 500 | 0.2 | 0.025 | Skewed | 4 | 1 | 100.00 | 99.98 | 100.00 | 100.00 |
| 500 | 0.2 | 0.025 | Skewed | 20 | 1 | 100.00 | 99.92 | 100.00 | 100.00 |
| 500 | 0.2 | 0.075 | Even | 4 | 1 | 100.00 | 99.96 | 100.00 | 99.98 |
| 500 | 0.2 | 0.075 | Even | 20 | 1 | 100.00 | 99.92 | 100.00 | 99.94 |
| 500 | 0.2 | 0.075 | Skewed | 4 | 1 | 99.98 | 99.94 | 100.00 | 100.00 |
| 500 | 0.2 | 0.075 | Skewed | 20 | 1 | 100.00 | 100.00 | 100.00 | 100.00 |
| 500 | 0.5 | 0.025 | Even | 4 | 1 | 100.00 | 99.94 | 100.00 | 99.98 |
| 500 | 0.5 | 0.025 | Even | 20 | 1 | 100.00 | 99.96 | 100.00 | 99.94 |
| 500 | 0.5 | 0.025 | Skewed | 4 | 1 | 100.00 | 99.86 | 100.00 | 100.00 |
| 500 | 0.5 | 0.025 | Skewed | 20 | 1 | 100.00 | 99.86 | 100.00 | 100.00 |
| 500 | 0.5 | 0.075 | Even | 4 | 1 | 100.00 | 99.88 | 100.00 | 99.96 |
| 500 | 0.5 | 0.075 | Even | 20 | 1 | 100.00 | 99.94 | 100.00 | 99.96 |
| 500 | 0.5 | 0.075 | Skewed | 4 | 1 | 100.00 | 99.82 | 100.00 | 100.00 |
| 500 | 0.5 | 0.075 | Skewed | 20 | 1 | 100.00 | 99.74 | 100.00 | 100.00 |
| 1000 | 0.2 | 0.025 | Even | 4 | 1 | 100.00 | 100.00 | 100.00 | 100.00 |
| 1000 | 0.2 | 0.025 | Even | 20 | 1 | 100.00 | 99.96 | 100.00 | 100.00 |
| 1000 | 0.2 | 0.025 | Skewed | 4 | 1 | 100.00 | 100.00 | 100.00 | 100.00 |
| 1000 | 0.2 | 0.025 | Skewed | 20 | 1 | 100.00 | 100.00 | 100.00 | 100.00 |
| 1000 | 0.2 | 0.075 | Even | 4 | 1 | 100.00 | 99.94 | 100.00 | 99.98 |
| 1000 | 0.2 | 0.075 | Even | 20 | 1 | 100.00 | 99.96 | 100.00 | 100.00 |
| 1000 | 0.2 | 0.075 | Skewed | 4 | 1 | 100.00 | 99.88 | 100.00 | 100.00 |
| 1000 | 0.2 | 0.075 | Skewed | 20 | 1 | 100.00 | 99.94 | 100.00 | 100.00 |
| 1000 | 0.5 | 0.025 | Even | 4 | 1 | 100.00 | 99.96 | 100.00 | 99.98 |
| 1000 | 0.5 | 0.025 | Even | 20 | 1 | 100.00 | 99.98 | 100.00 | 99.98 |
| 1000 | 0.5 | 0.025 | Skewed | 4 | 1 | 100.00 | 99.84 | 100.00 | 100.00 |
| 1000 | 0.5 | 0.025 | Skewed | 20 | 1 | 100.00 | 99.90 | 100.00 | 100.00 |
| 1000 | 0.5 | 0.075 | Even | 4 | 1 | 100.00 | 99.84 | 100.00 | 100.00 |
| 1000 | 0.5 | 0.075 | Even | 20 | 1 | 100.00 | 99.82 | 100.00 | 99.96 |
| 1000 | 0.5 | 0.075 | Skewed | 4 | 1 | 100.00 | 99.62 | 100.00 | 100.00 |
| 1000 | 0.5 | 0.075 | Skewed | 20 | 1 | 100.00 | 99.68 | 100.00 | 100.00 |
| 2000 | 0.2 | 0.025 | Even | 4 | 1 | 100.00 | 100.00 | 100.00 | 100.00 |
| 2000 | 0.2 | 0.025 | Even | 20 | 1 | 100.00 | 99.98 | 100.00 | 100.00 |
| 2000 | 0.2 | 0.025 | Skewed | 4 | 1 | 100.00 | 100.00 | 100.00 | 100.00 |
| 2000 | 0.2 | 0.025 | Skewed | 20 | 1 | 100.00 | 100.00 | 100.00 | 100.00 |
| 2000 | 0.2 | 0.075 | Even | 4 | 1 | 100.00 | 99.92 | 100.00 | 100.00 |
| 2000 | 0.2 | 0.075 | Even | 20 | 1 | 100.00 | 99.86 | 100.00 | 100.00 |
| 2000 | 0.2 | 0.075 | Skewed | 4 | 1 | 100.00 | 99.88 | 99.88 | 100.00 |
| 2000 | 0.2 | 0.075 | Skewed | 20 | 1 | 100.00 | 99.96 | 99.96 | 100.00 |
| 2000 | 0.5 | 0.025 | Even | 4 | 1 | 100.00 | 99.84 | 100.00 | 99.98 |
| 2000 | 0.5 | 0.025 | Even | 20 | 1 | 100.00 | 99.90 | 100.00 | 100.00 |
| 2000 | 0.5 | 0.025 | Skewed | 4 | 1 | 100.00 | 99.74 | 99.78 | 100.00 |
| 2000 | 0.5 | 0.025 | Skewed | 20 | 1 | 100.00 | 99.88 | 99.90 | 100.00 |
| 2000 | 0.5 | 0.075 | Even | 4 | 1 | 100.00 | 99.88 | 100.00 | 100.00 |
| 2000 | 0.5 | 0.075 | Even | 20 | 1 | 100.00 | 99.98 | 100.00 | 99.98 |
| 2000 | 0.5 | 0.075 | Skewed | 4 | 1 | 100.00 | 99.76 | 99.78 | 100.00 |
| 2000 | 0.5 | 0.075 | Skewed | 20 | 1 | 100.00 | 99.78 | 99.80 | 100.00 |

**Table 22 – Mean treatment effect – 5 centres, true OR<1**

| Sample size | Event rate | ICC | Patient distribution | Block size | True OR | Fixed effects | Random effects | GEE | MH |
| --- | --- | --- | --- | --- | --- | --- | --- | --- | --- |
| 200 | 0.2 | 0.025 | Even | 4 | 0.25 | 0.22 | 0.22 | 0.22 | 0.23 |
| 200 | 0.2 | 0.025 | Even | 20 | 0.25 | 0.22 | 0.21 | 0.21 | 0.23 |
| 200 | 0.2 | 0.025 | Skewed | 4 | 0.25 | 0.22 | 0.22 | 0.20 | 0.23 |
| 200 | 0.2 | 0.025 | Skewed | 20 | 0.25 | 0.22 | 0.21 | 0.22 | 0.23 |
| 200 | 0.2 | 0.075 | Even | 4 | 0.25 | 0.23 | 0.22 | 0.22 | 0.23 |
| 200 | 0.2 | 0.075 | Even | 20 | 0.25 | 0.22 | 0.22 | 0.23 | 0.23 |
| 200 | 0.2 | 0.075 | Skewed | 4 | 0.25 | 0.23 | 0.22 | 0.22 | 0.23 |
| 200 | 0.2 | 0.075 | Skewed | 20 | 0.25 | 0.23 | 0.22 | 0.22 | 0.23 |
| 200 | 0.5 | 0.025 | Even | 4 | 0.42 | 0.41 | 0.42 | 0.42 | 0.42 |
| 200 | 0.5 | 0.025 | Even | 20 | 0.42 | 0.41 | 0.41 | 0.42 | 0.41 |
| 200 | 0.5 | 0.025 | Skewed | 4 | 0.42 | 0.41 | 0.41 | 0.42 | 0.41 |
| 200 | 0.5 | 0.025 | Skewed | 20 | 0.42 | 0.40 | 0.41 | 0.42 | 0.41 |
| 200 | 0.5 | 0.075 | Even | 4 | 0.42 | 0.41 | 0.42 | 0.43 | 0.41 |
| 200 | 0.5 | 0.075 | Even | 20 | 0.42 | 0.41 | 0.42 | 0.43 | 0.41 |
| 200 | 0.5 | 0.075 | Skewed | 4 | 0.42 | 0.41 | 0.42 | 0.43 | 0.42 |
| 200 | 0.5 | 0.075 | Skewed | 20 | 0.42 | 0.41 | 0.42 | 0.43 | 0.42 |
| 500 | 0.2 | 0.025 | Even | 4 | 0.47 | 0.47 | 0.47 | 0.47 | 0.47 |
| 500 | 0.2 | 0.025 | Even | 20 | 0.47 | 0.47 | 0.47 | 0.47 | 0.47 |
| 500 | 0.2 | 0.025 | Skewed | 4 | 0.47 | 0.47 | 0.47 | 0.47 | 0.47 |
| 500 | 0.2 | 0.025 | Skewed | 20 | 0.47 | 0.46 | 0.47 | 0.47 | 0.47 |
| 500 | 0.2 | 0.075 | Even | 4 | 0.47 | 0.47 | 0.47 | 0.48 | 0.47 |
| 500 | 0.2 | 0.075 | Even | 20 | 0.47 | 0.47 | 0.47 | 0.48 | 0.47 |
| 500 | 0.2 | 0.075 | Skewed | 4 | 0.47 | 0.47 | 0.47 | 0.48 | 0.47 |
| 500 | 0.2 | 0.075 | Skewed | 20 | 0.47 | 0.47 | 0.47 | 0.48 | 0.47 |
| 500 | 0.5 | 0.025 | Even | 4 | 0.59 | 0.58 | 0.59 | 0.59 | 0.59 |
| 500 | 0.5 | 0.025 | Even | 20 | 0.59 | 0.59 | 0.59 | 0.59 | 0.59 |
| 500 | 0.5 | 0.025 | Skewed | 4 | 0.59 | 0.58 | 0.59 | 0.59 | 0.59 |
| 500 | 0.5 | 0.025 | Skewed | 20 | 0.59 | 0.59 | 0.59 | 0.59 | 0.59 |
| 500 | 0.5 | 0.075 | Even | 4 | 0.59 | 0.58 | 0.59 | 0.60 | 0.59 |
| 500 | 0.5 | 0.075 | Even | 20 | 0.59 | 0.58 | 0.59 | 0.60 | 0.59 |
| 500 | 0.5 | 0.075 | Skewed | 4 | 0.59 | 0.59 | 0.59 | 0.60 | 0.59 |
| 500 | 0.5 | 0.075 | Skewed | 20 | 0.59 | 0.59 | 0.59 | 0.60 | 0.59 |
| 1000 | 0.2 | 0.025 | Even | 4 | 0.61 | 0.61 | 0.61 | 0.61 | 0.61 |
| 1000 | 0.2 | 0.025 | Even | 20 | 0.61 | 0.60 | 0.61 | 0.61 | 0.61 |
| 1000 | 0.2 | 0.025 | Skewed | 4 | 0.61 | 0.61 | 0.61 | 0.61 | 0.61 |
| 1000 | 0.2 | 0.025 | Skewed | 20 | 0.61 | 0.61 | 0.61 | 0.61 | 0.61 |
| 1000 | 0.2 | 0.075 | Even | 4 | 0.61 | 0.61 | 0.61 | 0.62 | 0.61 |
| 1000 | 0.2 | 0.075 | Even | 20 | 0.61 | 0.61 | 0.61 | 0.62 | 0.61 |
| 1000 | 0.2 | 0.075 | Skewed | 4 | 0.61 | 0.60 | 0.61 | 0.61 | 0.61 |
| 1000 | 0.2 | 0.075 | Skewed | 20 | 0.61 | 0.60 | 0.61 | 0.61 | 0.61 |
| 1000 | 0.5 | 0.025 | Even | 4 | 0.70 | 0.69 | 0.70 | 0.70 | 0.69 |
| 1000 | 0.5 | 0.025 | Even | 20 | 0.70 | 0.69 | 0.69 | 0.70 | 0.69 |
| 1000 | 0.5 | 0.025 | Skewed | 4 | 0.70 | 0.69 | 0.70 | 0.70 | 0.70 |
| 1000 | 0.5 | 0.025 | Skewed | 20 | 0.70 | 0.69 | 0.69 | 0.70 | 0.69 |
| 1000 | 0.5 | 0.075 | Even | 4 | 0.70 | 0.69 | 0.70 | 0.71 | 0.70 |
| 1000 | 0.5 | 0.075 | Even | 20 | 0.70 | 0.70 | 0.70 | 0.71 | 0.70 |
| 1000 | 0.5 | 0.075 | Skewed | 4 | 0.70 | 0.69 | 0.69 | 0.71 | 0.69 |
| 1000 | 0.5 | 0.075 | Skewed | 20 | 0.70 | 0.69 | 0.69 | 0.71 | 0.69 |
| 2000 | 0.2 | 0.025 | Even | 4 | 0.71 | 0.71 | 0.71 | 0.71 | 0.71 |
| 2000 | 0.2 | 0.025 | Even | 20 | 0.71 | 0.71 | 0.71 | 0.71 | 0.71 |
| 2000 | 0.2 | 0.025 | Skewed | 4 | 0.71 | 0.71 | 0.71 | 0.71 | 0.71 |
| 2000 | 0.2 | 0.025 | Skewed | 20 | 0.71 | 0.71 | 0.71 | 0.71 | 0.71 |
| 2000 | 0.2 | 0.075 | Even | 4 | 0.71 | 0.71 | 0.71 | 0.72 | 0.71 |
| 2000 | 0.2 | 0.075 | Even | 20 | 0.71 | 0.71 | 0.71 | 0.72 | 0.71 |
| 2000 | 0.2 | 0.075 | Skewed | 4 | 0.71 | 0.71 | 0.71 | 0.71 | 0.71 |
| 2000 | 0.2 | 0.075 | Skewed | 20 | 0.71 | 0.71 | 0.71 | 0.72 | 0.71 |
| 2000 | 0.5 | 0.025 | Even | 4 | 0.77 | 0.77 | 0.77 | 0.77 | 0.77 |
| 2000 | 0.5 | 0.025 | Even | 20 | 0.77 | 0.77 | 0.77 | 0.77 | 0.77 |
| 2000 | 0.5 | 0.025 | Skewed | 4 | 0.77 | 0.77 | 0.77 | 0.77 | 0.77 |
| 2000 | 0.5 | 0.025 | Skewed | 20 | 0.77 | 0.77 | 0.77 | 0.77 | 0.77 |
| 2000 | 0.5 | 0.075 | Even | 4 | 0.77 | 0.77 | 0.77 | 0.78 | 0.77 |
| 2000 | 0.5 | 0.075 | Even | 20 | 0.77 | 0.77 | 0.77 | 0.78 | 0.77 |
| 2000 | 0.5 | 0.075 | Skewed | 4 | 0.77 | 0.77 | 0.77 | 0.77 | 0.77 |
| 2000 | 0.5 | 0.075 | Skewed | 20 | 0.77 | 0.77 | 0.77 | 0.77 | 0.77 |

**Table 23 – Power – 5 centres, true OR<1**

| Sample size | Event rate | ICC | Patient distribution | Block size | True OR | Fixed effects | Random effects | GEE | MH |
| --- | --- | --- | --- | --- | --- | --- | --- | --- | --- |
| 200 | 0.2 | 0.025 | Even | 4 | 0.25 | 86.64 | 85.98 | 85.70 | 85.90 |
| 200 | 0.2 | 0.025 | Even | 20 | 0.25 | 87.40 | 86.84 | 86.62 | 86.88 |
| 200 | 0.2 | 0.025 | Skewed | 4 | 0.25 | 86.26 | 85.72 | 85.74 | 85.32 |
| 200 | 0.2 | 0.025 | Skewed | 20 | 0.25 | 86.34 | 86.06 | 86.18 | 85.76 |
| 200 | 0.2 | 0.075 | Even | 4 | 0.25 | 86.34 | 86.34 | 85.72 | 86.26 |
| 200 | 0.2 | 0.075 | Even | 20 | 0.25 | 86.80 | 86.44 | 85.76 | 86.08 |
| 200 | 0.2 | 0.075 | Skewed | 4 | 0.25 | 85.94 | 85.36 | 84.94 | 85.22 |
| 200 | 0.2 | 0.075 | Skewed | 20 | 0.25 | 85.96 | 85.36 | 85.04 | 85.10 |
| 200 | 0.5 | 0.025 | Even | 4 | 0.42 | 84.16 | 83.92 | 83.90 | 83.32 |
| 200 | 0.5 | 0.025 | Even | 20 | 0.42 | 85.00 | 84.78 | 84.74 | 84.40 |
| 200 | 0.5 | 0.025 | Skewed | 4 | 0.42 | 84.44 | 84.14 | 83.94 | 83.32 |
| 200 | 0.5 | 0.025 | Skewed | 20 | 0.42 | 84.84 | 84.35 | 84.08 | 83.68 |
| 200 | 0.5 | 0.075 | Even | 4 | 0.42 | 81.96 | 81.47 | 81.44 | 80.96 |
| 200 | 0.5 | 0.075 | Even | 20 | 0.42 | 83.08 | 82.55 | 82.52 | 81.98 |
| 200 | 0.5 | 0.075 | Skewed | 4 | 0.42 | 83.36 | 82.99 | 82.86 | 82.14 |
| 200 | 0.5 | 0.075 | Skewed | 20 | 0.42 | 82.18 | 81.72 | 81.72 | 80.96 |
| 500 | 0.2 | 0.025 | Even | 4 | 0.47 | 83.50 | 83.14 | 83.16 | 83.24 |
| 500 | 0.2 | 0.025 | Even | 20 | 0.47 | 84.24 | 84.08 | 84.00 | 83.88 |
| 500 | 0.2 | 0.025 | Skewed | 4 | 0.47 | 83.82 | 83.52 | 83.52 | 83.32 |
| 500 | 0.2 | 0.025 | Skewed | 20 | 0.47 | 83.72 | 83.56 | 83.32 | 83.22 |
| 500 | 0.2 | 0.075 | Even | 4 | 0.47 | 83.90 | 83.78 | 83.24 | 83.60 |
| 500 | 0.2 | 0.075 | Even | 20 | 0.47 | 83.54 | 83.32 | 82.88 | 83.10 |
| 500 | 0.2 | 0.075 | Skewed | 4 | 0.47 | 84.08 | 83.82 | 83.54 | 83.76 |
| 500 | 0.2 | 0.075 | Skewed | 20 | 0.47 | 83.66 | 83.28 | 82.92 | 83.16 |
| 500 | 0.5 | 0.025 | Even | 4 | 0.59 | 83.06 | 82.97 | 82.96 | 82.64 |
| 500 | 0.5 | 0.025 | Even | 20 | 0.59 | 82.52 | 82.44 | 82.42 | 82.36 |
| 500 | 0.5 | 0.025 | Skewed | 4 | 0.59 | 83.30 | 83.26 | 83.26 | 82.94 |
| 500 | 0.5 | 0.025 | Skewed | 20 | 0.59 | 83.02 | 82.74 | 82.74 | 82.50 |
| 500 | 0.5 | 0.075 | Even | 4 | 0.59 | 81.28 | 81.04 | 81.06 | 80.72 |
| 500 | 0.5 | 0.075 | Even | 20 | 0.59 | 81.26 | 81.02 | 81.02 | 80.82 |
| 500 | 0.5 | 0.075 | Skewed | 4 | 0.59 | 80.22 | 80.03 | 80.00 | 79.86 |
| 500 | 0.5 | 0.075 | Skewed | 20 | 0.59 | 80.70 | 80.37 | 80.34 | 80.16 |
| 1000 | 0.2 | 0.025 | Even | 4 | 0.61 | 82.42 | 82.24 | 82.26 | 82.20 |
| 1000 | 0.2 | 0.025 | Even | 20 | 0.61 | 83.36 | 83.22 | 83.16 | 83.12 |
| 1000 | 0.2 | 0.025 | Skewed | 4 | 0.61 | 82.12 | 81.88 | 81.76 | 81.86 |
| 1000 | 0.2 | 0.025 | Skewed | 20 | 0.61 | 83.22 | 83.14 | 82.92 | 82.98 |
| 1000 | 0.2 | 0.075 | Even | 4 | 0.61 | 82.50 | 82.34 | 81.90 | 82.24 |
| 1000 | 0.2 | 0.075 | Even | 20 | 0.61 | 82.30 | 82.22 | 82.00 | 82.20 |
| 1000 | 0.2 | 0.075 | Skewed | 4 | 0.61 | 82.52 | 82.26 | 81.86 | 82.32 |
| 1000 | 0.2 | 0.075 | Skewed | 20 | 0.61 | 82.32 | 82.32 | 81.92 | 82.26 |
| 1000 | 0.5 | 0.025 | Even | 4 | 0.70 | 81.34 | 81.27 | 81.22 | 81.10 |
| 1000 | 0.5 | 0.025 | Even | 20 | 0.70 | 81.82 | 81.79 | 81.78 | 81.74 |
| 1000 | 0.5 | 0.025 | Skewed | 4 | 0.70 | 80.82 | 80.71 | 80.70 | 80.52 |
| 1000 | 0.5 | 0.025 | Skewed | 20 | 0.70 | 80.46 | 80.30 | 80.36 | 80.14 |
| 1000 | 0.5 | 0.075 | Even | 4 | 0.70 | 79.64 | 79.41 | 79.44 | 79.32 |
| 1000 | 0.5 | 0.075 | Even | 20 | 0.70 | 78.34 | 78.21 | 78.22 | 78.16 |
| 1000 | 0.5 | 0.075 | Skewed | 4 | 0.70 | 79.44 | 79.33 | 79.38 | 79.18 |
| 1000 | 0.5 | 0.075 | Skewed | 20 | 0.70 | 79.70 | 79.55 | 79.52 | 79.40 |
| 2000 | 0.2 | 0.025 | Even | 4 | 0.71 | 82.78 | 82.64 | 82.56 | 82.62 |
| 2000 | 0.2 | 0.025 | Even | 20 | 0.71 | 83.14 | 83.14 | 83.10 | 83.10 |
| 2000 | 0.2 | 0.025 | Skewed | 4 | 0.71 | 82.26 | 82.16 | 82.16 | 82.12 |
| 2000 | 0.2 | 0.025 | Skewed | 20 | 0.71 | 81.50 | 81.46 | 81.46 | 81.36 |
| 2000 | 0.2 | 0.075 | Even | 4 | 0.71 | 82.30 | 82.16 | 81.54 | 82.10 |
| 2000 | 0.2 | 0.075 | Even | 20 | 0.71 | 82.80 | 82.72 | 82.36 | 82.70 |
| 2000 | 0.2 | 0.075 | Skewed | 4 | 0.71 | 82.32 | 82.20 | 82.20 | 82.22 |
| 2000 | 0.2 | 0.075 | Skewed | 20 | 0.71 | 82.46 | 82.42 | 82.42 | 82.38 |
| 2000 | 0.5 | 0.025 | Even | 4 | 0.77 | 82.10 | 82.06 | 82.06 | 82.00 |
| 2000 | 0.5 | 0.025 | Even | 20 | 0.77 | 81.84 | 81.84 | 81.82 | 81.82 |
| 2000 | 0.5 | 0.025 | Skewed | 4 | 0.77 | 82.46 | 82.40 | 82.41 | 82.34 |
| 2000 | 0.5 | 0.025 | Skewed | 20 | 0.77 | 82.46 | 82.45 | 82.45 | 82.42 |
| 2000 | 0.5 | 0.075 | Even | 4 | 0.77 | 80.60 | 80.60 | 80.58 | 80.58 |
| 2000 | 0.5 | 0.075 | Even | 20 | 0.77 | 80.84 | 80.76 | 80.76 | 80.74 |
| 2000 | 0.5 | 0.075 | Skewed | 4 | 0.77 | 81.54 | 81.52 | 81.51 | 81.40 |
| 2000 | 0.5 | 0.075 | Skewed | 20 | 0.77 | 80.58 | 80.57 | 80.56 | 80.44 |

**Table 24 – Convergence – 5 centres, true OR<1**

| Sample size | Event rate | ICC | Patient distribution | Block size | True OR | Fixed effects | Random effects | GEE | MH |
| --- | --- | --- | --- | --- | --- | --- | --- | --- | --- |
| 200 | 0.2 | 0.025 | Even | 4 | 0.25 | 99.76 | 99.84 | 100.00 | 100.00 |
| 200 | 0.2 | 0.025 | Even | 20 | 0.25 | 99.64 | 99.88 | 100.00 | 100.00 |
| 200 | 0.2 | 0.025 | Skewed | 4 | 0.25 | 99.92 | 99.90 | 99.98 | 100.00 |
| 200 | 0.2 | 0.025 | Skewed | 20 | 0.25 | 99.88 | 99.90 | 99.96 | 100.00 |
| 200 | 0.2 | 0.075 | Even | 4 | 0.25 | 99.20 | 99.92 | 100.00 | 100.00 |
| 200 | 0.2 | 0.075 | Even | 20 | 0.25 | 99.26 | 99.84 | 100.00 | 100.00 |
| 200 | 0.2 | 0.075 | Skewed | 4 | 0.25 | 99.88 | 99.86 | 99.94 | 100.00 |
| 200 | 0.2 | 0.075 | Skewed | 20 | 0.25 | 99.82 | 99.98 | 99.98 | 100.00 |
| 200 | 0.5 | 0.025 | Even | 4 | 0.42 | 100.00 | 99.88 | 100.00 | 100.00 |
| 200 | 0.5 | 0.025 | Even | 20 | 0.42 | 100.00 | 99.88 | 100.00 | 99.98 |
| 200 | 0.5 | 0.025 | Skewed | 4 | 0.42 | 100.00 | 99.88 | 100.00 | 100.00 |
| 200 | 0.5 | 0.025 | Skewed | 20 | 0.42 | 100.00 | 99.92 | 100.00 | 100.00 |
| 200 | 0.5 | 0.075 | Even | 4 | 0.42 | 100.00 | 99.96 | 100.00 | 100.00 |
| 200 | 0.5 | 0.075 | Even | 20 | 0.42 | 100.00 | 99.96 | 100.00 | 100.00 |
| 200 | 0.5 | 0.075 | Skewed | 4 | 0.42 | 99.98 | 99.96 | 100.00 | 100.00 |
| 200 | 0.5 | 0.075 | Skewed | 20 | 0.42 | 99.98 | 99.90 | 100.00 | 100.00 |
| 500 | 0.2 | 0.025 | Even | 4 | 0.47 | 100.00 | 99.84 | 100.00 | 100.00 |
| 500 | 0.2 | 0.025 | Even | 20 | 0.47 | 100.00 | 99.88 | 100.00 | 100.00 |
| 500 | 0.2 | 0.025 | Skewed | 4 | 0.47 | 100.00 | 99.90 | 100.00 | 100.00 |
| 500 | 0.2 | 0.025 | Skewed | 20 | 0.47 | 100.00 | 99.92 | 100.00 | 100.00 |
| 500 | 0.2 | 0.075 | Even | 4 | 0.47 | 100.00 | 99.96 | 100.00 | 100.00 |
| 500 | 0.2 | 0.075 | Even | 20 | 0.47 | 100.00 | 99.94 | 100.00 | 100.00 |
| 500 | 0.2 | 0.075 | Skewed | 4 | 0.47 | 100.00 | 99.94 | 100.00 | 100.00 |
| 500 | 0.2 | 0.075 | Skewed | 20 | 0.47 | 99.98 | 99.94 | 100.00 | 100.00 |
| 500 | 0.5 | 0.025 | Even | 4 | 0.59 | 100.00 | 99.96 | 100.00 | 100.00 |
| 500 | 0.5 | 0.025 | Even | 20 | 0.59 | 100.00 | 99.98 | 100.00 | 100.00 |
| 500 | 0.5 | 0.025 | Skewed | 4 | 0.59 | 100.00 | 99.88 | 100.00 | 100.00 |
| 500 | 0.5 | 0.025 | Skewed | 20 | 0.59 | 100.00 | 99.90 | 100.00 | 100.00 |
| 500 | 0.5 | 0.075 | Even | 4 | 0.59 | 100.00 | 99.92 | 100.00 | 100.00 |
| 500 | 0.5 | 0.075 | Even | 20 | 0.59 | 100.00 | 99.92 | 100.00 | 100.00 |
| 500 | 0.5 | 0.075 | Skewed | 4 | 0.59 | 100.00 | 99.86 | 100.00 | 100.00 |
| 500 | 0.5 | 0.075 | Skewed | 20 | 0.59 | 100.00 | 99.86 | 100.00 | 100.00 |
| 1000 | 0.2 | 0.025 | Even | 4 | 0.61 | 100.00 | 99.92 | 100.00 | 100.00 |
| 1000 | 0.2 | 0.025 | Even | 20 | 0.61 | 100.00 | 99.96 | 100.00 | 100.00 |
| 1000 | 0.2 | 0.025 | Skewed | 4 | 0.61 | 100.00 | 99.94 | 100.00 | 100.00 |
| 1000 | 0.2 | 0.025 | Skewed | 20 | 0.61 | 100.00 | 100.00 | 100.00 | 100.00 |
| 1000 | 0.2 | 0.075 | Even | 4 | 0.61 | 100.00 | 99.96 | 100.00 | 100.00 |
| 1000 | 0.2 | 0.075 | Even | 20 | 0.61 | 100.00 | 100.00 | 100.00 | 100.00 |
| 1000 | 0.2 | 0.075 | Skewed | 4 | 0.61 | 100.00 | 99.88 | 100.00 | 100.00 |
| 1000 | 0.2 | 0.075 | Skewed | 20 | 0.61 | 100.00 | 99.96 | 100.00 | 100.00 |
| 1000 | 0.5 | 0.025 | Even | 4 | 0.70 | 100.00 | 99.92 | 100.00 | 100.00 |
| 1000 | 0.5 | 0.025 | Even | 20 | 0.70 | 100.00 | 99.96 | 100.00 | 100.00 |
| 1000 | 0.5 | 0.025 | Skewed | 4 | 0.70 | 100.00 | 99.86 | 100.00 | 100.00 |
| 1000 | 0.5 | 0.025 | Skewed | 20 | 0.70 | 100.00 | 99.90 | 100.00 | 100.00 |
| 1000 | 0.5 | 0.075 | Even | 4 | 0.70 | 100.00 | 99.94 | 100.00 | 100.00 |
| 1000 | 0.5 | 0.075 | Even | 20 | 0.70 | 100.00 | 99.88 | 100.00 | 100.00 |
| 1000 | 0.5 | 0.075 | Skewed | 4 | 0.70 | 100.00 | 99.78 | 100.00 | 100.00 |
| 1000 | 0.5 | 0.075 | Skewed | 20 | 0.70 | 100.00 | 99.74 | 100.00 | 100.00 |
| 2000 | 0.2 | 0.025 | Even | 4 | 0.71 | 100.00 | 99.98 | 100.00 | 100.00 |
| 2000 | 0.2 | 0.025 | Even | 20 | 0.71 | 100.00 | 99.94 | 100.00 | 100.00 |
| 2000 | 0.2 | 0.025 | Skewed | 4 | 0.71 | 100.00 | 99.98 | 100.00 | 100.00 |
| 2000 | 0.2 | 0.025 | Skewed | 20 | 0.71 | 100.00 | 99.96 | 100.00 | 100.00 |
| 2000 | 0.2 | 0.075 | Even | 4 | 0.71 | 100.00 | 99.94 | 100.00 | 100.00 |
| 2000 | 0.2 | 0.075 | Even | 20 | 0.71 | 100.00 | 99.98 | 100.00 | 100.00 |
| 2000 | 0.2 | 0.075 | Skewed | 4 | 0.71 | 100.00 | 99.94 | 99.94 | 100.00 |
| 2000 | 0.2 | 0.075 | Skewed | 20 | 0.71 | 100.00 | 99.90 | 99.94 | 100.00 |
| 2000 | 0.5 | 0.025 | Even | 4 | 0.77 | 100.00 | 99.98 | 100.00 | 100.00 |
| 2000 | 0.5 | 0.025 | Even | 20 | 0.77 | 100.00 | 99.90 | 100.00 | 100.00 |
| 2000 | 0.5 | 0.025 | Skewed | 4 | 0.77 | 100.00 | 99.78 | 99.82 | 100.00 |
| 2000 | 0.5 | 0.025 | Skewed | 20 | 0.77 | 100.00 | 99.92 | 99.96 | 100.00 |
| 2000 | 0.5 | 0.075 | Even | 4 | 0.77 | 100.00 | 99.78 | 100.00 | 100.00 |
| 2000 | 0.5 | 0.075 | Even | 20 | 0.77 | 100.00 | 99.90 | 100.00 | 100.00 |
| 2000 | 0.5 | 0.075 | Skewed | 4 | 0.77 | 100.00 | 99.80 | 99.82 | 100.00 |
| 2000 | 0.5 | 0.075 | Skewed | 20 | 0.77 | 100.00 | 99.86 | 99.88 | 100.00 |

**Table 25 – Mean treatment effect – 50 centres, true OR=1**

| Sample size | Event rate | ICC | Patient distribution | Block size | True OR | Fixed effects | Random effects | GEE | MH |
| --- | --- | --- | --- | --- | --- | --- | --- | --- | --- |
| 200 | 0.2 | 0.025 | Even | 4 | 1.00 | 1.00 | 1.00 | 1.00 | 1.00 |
| 200 | 0.2 | 0.025 | Even | 20 | 1.00 | 1.00 | 1.00 | 1.00 | 1.00 |
| 200 | 0.2 | 0.025 | Skewed | 4 | 1.00 | 1.00 | 1.00 | 1.00 | 1.00 |
| 200 | 0.2 | 0.025 | Skewed | 20 | 1.00 | 1.00 | 1.00 | 1.00 | 1.00 |
| 200 | 0.2 | 0.075 | Even | 4 | 1.00 | 1.01 | 1.00 | 1.00 | 1.00 |
| 200 | 0.2 | 0.075 | Even | 20 | 1.00 | 1.01 | 1.00 | 1.00 | 1.00 |
| 200 | 0.2 | 0.075 | Skewed | 4 | 1.00 | 1.00 | 1.00 | 1.00 | 1.00 |
| 200 | 0.2 | 0.075 | Skewed | 20 | 1.00 | 0.99 | 0.99 | 0.99 | 0.99 |
| 200 | 0.5 | 0.025 | Even | 4 | 1.00 | 1.00 | 1.00 | 1.00 | 1.00 |
| 200 | 0.5 | 0.025 | Even | 20 | 1.00 | 0.99 | 0.99 | 0.99 | 0.99 |
| 200 | 0.5 | 0.025 | Skewed | 4 | 1.00 | 1.00 | 1.00 | 1.00 | 1.00 |
| 200 | 0.5 | 0.025 | Skewed | 20 | 1.00 | 1.00 | 1.00 | 1.00 | 1.00 |
| 200 | 0.5 | 0.075 | Even | 4 | 1.00 | 1.00 | 1.00 | 1.00 | 1.00 |
| 200 | 0.5 | 0.075 | Even | 20 | 1.00 | 1.01 | 1.01 | 1.01 | 1.01 |
| 200 | 0.5 | 0.075 | Skewed | 4 | 1.00 | 1.00 | 1.00 | 1.00 | 1.00 |
| 200 | 0.5 | 0.075 | Skewed | 20 | 1.00 | 1.00 | 1.00 | 1.00 | 1.00 |
| 500 | 0.2 | 0.025 | Even | 4 | 1.00 | 1.01 | 1.00 | 1.00 | 1.00 |
| 500 | 0.2 | 0.025 | Even | 20 | 1.00 | 1.00 | 1.00 | 1.00 | 1.00 |
| 500 | 0.2 | 0.025 | Skewed | 4 | 1.00 | 1.00 | 1.00 | 1.00 | 1.00 |
| 500 | 0.2 | 0.025 | Skewed | 20 | 1.00 | 1.00 | 1.00 | 1.00 | 1.00 |
| 500 | 0.2 | 0.075 | Even | 4 | 1.00 | 1.00 | 1.00 | 1.00 | 1.00 |
| 500 | 0.2 | 0.075 | Even | 20 | 1.00 | 1.00 | 1.00 | 1.00 | 1.00 |
| 500 | 0.2 | 0.075 | Skewed | 4 | 1.00 | 1.00 | 1.00 | 1.00 | 1.00 |
| 500 | 0.2 | 0.075 | Skewed | 20 | 1.00 | 1.00 | 1.00 | 1.00 | 1.00 |
| 500 | 0.5 | 0.025 | Even | 4 | 1.00 | 1.00 | 1.00 | 1.00 | 1.00 |
| 500 | 0.5 | 0.025 | Even | 20 | 1.00 | 1.01 | 1.01 | 1.01 | 1.01 |
| 500 | 0.5 | 0.025 | Skewed | 4 | 1.00 | 1.00 | 1.00 | 1.00 | 1.00 |
| 500 | 0.5 | 0.025 | Skewed | 20 | 1.00 | 1.00 | 1.00 | 1.00 | 1.00 |
| 500 | 0.5 | 0.075 | Even | 4 | 1.00 | 1.00 | 1.00 | 1.00 | 1.00 |
| 500 | 0.5 | 0.075 | Even | 20 | 1.00 | 1.01 | 1.00 | 1.00 | 1.00 |
| 500 | 0.5 | 0.075 | Skewed | 4 | 1.00 | 1.00 | 1.00 | 1.00 | 1.00 |
| 500 | 0.5 | 0.075 | Skewed | 20 | 1.00 | 1.00 | 1.00 | 1.00 | 1.00 |
| 1000 | 0.2 | 0.025 | Even | 4 | 1.00 | 1.00 | 1.00 | 1.00 | 1.00 |
| 1000 | 0.2 | 0.025 | Even | 20 | 1.00 | 0.99 | 0.99 | 0.99 | 0.99 |
| 1000 | 0.2 | 0.025 | Skewed | 4 | 1.00 | 1.00 | 1.00 | 1.00 | 1.00 |
| 1000 | 0.2 | 0.025 | Skewed | 20 | 1.00 | 1.00 | 1.00 | 1.00 | 1.00 |
| 1000 | 0.2 | 0.075 | Even | 4 | 1.00 | 1.00 | 1.00 | 1.00 | 1.00 |
| 1000 | 0.2 | 0.075 | Even | 20 | 1.00 | 1.00 | 1.00 | 1.00 | 1.00 |
| 1000 | 0.2 | 0.075 | Skewed | 4 | 1.00 | 1.00 | 1.00 | 1.00 | 1.00 |
| 1000 | 0.2 | 0.075 | Skewed | 20 | 1.00 | 1.00 | 1.00 | 1.00 | 1.00 |
| 1000 | 0.5 | 0.025 | Even | 4 | 1.00 | 1.00 | 1.00 | 1.00 | 1.00 |
| 1000 | 0.5 | 0.025 | Even | 20 | 1.00 | 1.00 | 1.00 | 1.00 | 1.00 |
| 1000 | 0.5 | 0.025 | Skewed | 4 | 1.00 | 1.00 | 1.00 | 1.00 | 1.00 |
| 1000 | 0.5 | 0.025 | Skewed | 20 | 1.00 | 1.00 | 1.00 | 1.00 | 1.00 |
| 1000 | 0.5 | 0.075 | Even | 4 | 1.00 | 1.00 | 1.00 | 1.00 | 1.00 |
| 1000 | 0.5 | 0.075 | Even | 20 | 1.00 | 1.00 | 1.00 | 1.00 | 1.00 |
| 1000 | 0.5 | 0.075 | Skewed | 4 | 1.00 | 1.00 | 1.00 | 1.00 | 1.00 |
| 1000 | 0.5 | 0.075 | Skewed | 20 | 1.00 | 1.00 | 1.00 | 1.00 | 1.00 |
| 2000 | 0.2 | 0.025 | Even | 4 | 1.00 | 1.00 | 1.00 | 1.00 | 1.00 |
| 2000 | 0.2 | 0.025 | Even | 20 | 1.00 | 1.00 | 1.00 | 1.00 | 1.00 |
| 2000 | 0.2 | 0.025 | Skewed | 4 | 1.00 | 1.00 | 1.00 | 1.00 | 1.00 |
| 2000 | 0.2 | 0.025 | Skewed | 20 | 1.00 | 1.00 | 1.00 | 1.03 | 1.00 |
| 2000 | 0.2 | 0.075 | Even | 4 | 1.00 | 1.00 | 1.00 | 1.00 | 1.00 |
| 2000 | 0.2 | 0.075 | Even | 20 | 1.00 | 1.00 | 1.00 | 1.00 | 1.00 |
| 2000 | 0.2 | 0.075 | Skewed | 4 | 1.00 | 1.00 | 1.00 | 1.02 | 1.00 |
| 2000 | 0.2 | 0.075 | Skewed | 20 | 1.00 | 1.00 | 1.00 | 1.00 | 1.00 |
| 2000 | 0.5 | 0.025 | Even | 4 | 1.00 | 1.00 | 1.00 | 1.00 | 1.00 |
| 2000 | 0.5 | 0.025 | Even | 20 | 1.00 | 1.00 | 1.00 | 1.00 | 1.00 |
| 2000 | 0.5 | 0.025 | Skewed | 4 | 1.00 | 1.00 | 1.00 | 1.00 | 1.00 |
| 2000 | 0.5 | 0.025 | Skewed | 20 | 1.00 | 1.00 | 1.00 | 1.00 | 1.00 |
| 2000 | 0.5 | 0.075 | Even | 4 | 1.00 | 1.00 | 1.00 | 1.00 | 1.00 |
| 2000 | 0.5 | 0.075 | Even | 20 | 1.00 | 1.00 | 1.00 | 1.00 | 1.00 |
| 2000 | 0.5 | 0.075 | Skewed | 4 | 1.00 | 1.00 | 1.00 | 0.99 | 1.00 |
| 2000 | 0.5 | 0.075 | Skewed | 20 | 1.00 | 1.00 | 1.00 | 1.01 | 1.00 |

**Table 26 – Type I error rates – 50 centres, true OR=1**

| Sample size | Event rate | ICC | Patient distribution | Block size | True OR | Fixed effects | Random effects | GEE | MH |
| --- | --- | --- | --- | --- | --- | --- | --- | --- | --- |
| 200 | 0.2 | 0.025 | Even | 4 | 1.00 | 8.04 | 4.80 | 4.72 | 4.43 |
| 200 | 0.2 | 0.025 | Even | 20 | 1.00 | 8.00 | 4.62 | 4.82 | 4.22 |
| 200 | 0.2 | 0.025 | Skewed | 4 | 1.00 | 7.08 | 5.08 | 4.98 | 4.44 |
| 200 | 0.2 | 0.025 | Skewed | 20 | 1.00 | 7.04 | 5.26 | 5.52 | 4.66 |
| 200 | 0.2 | 0.075 | Even | 4 | 1.00 | 9.48 | 5.19 | 5.18 | 4.84 |
| 200 | 0.2 | 0.075 | Even | 20 | 1.00 | 8.76 | 4.60 | 4.86 | 4.70 |
| 200 | 0.2 | 0.075 | Skewed | 4 | 1.00 | 7.18 | 4.58 | 4.60 | 4.08 |
| 200 | 0.2 | 0.075 | Skewed | 20 | 1.00 | 7.12 | 4.96 | 5.16 | 4.44 |
| 200 | 0.5 | 0.025 | Even | 4 | 1.00 | 9.62 | 5.96 | 5.58 | 5.21 |
| 200 | 0.5 | 0.025 | Even | 20 | 1.00 | 8.34 | 4.54 | 4.70 | 4.12 |
| 200 | 0.5 | 0.025 | Skewed | 4 | 1.00 | 6.82 | 4.92 | 4.86 | 4.38 |
| 200 | 0.5 | 0.025 | Skewed | 20 | 1.00 | 6.90 | 4.90 | 4.90 | 4.32 |
| 200 | 0.5 | 0.075 | Even | 4 | 1.00 | 8.56 | 5.27 | 5.08 | 4.78 |
| 200 | 0.5 | 0.075 | Even | 20 | 1.00 | 8.10 | 4.59 | 4.92 | 3.96 |
| 200 | 0.5 | 0.075 | Skewed | 4 | 1.00 | 7.20 | 4.82 | 4.84 | 4.30 |
| 200 | 0.5 | 0.075 | Skewed | 20 | 1.00 | 6.90 | 4.50 | 4.70 | 4.50 |
| 500 | 0.2 | 0.025 | Even | 4 | 1.00 | 6.70 | 5.42 | 5.40 | 5.46 |
| 500 | 0.2 | 0.025 | Even | 20 | 1.00 | 5.98 | 4.94 | 4.90 | 4.80 |
| 500 | 0.2 | 0.025 | Skewed | 4 | 1.00 | 5.77 | 4.85 | 4.82 | 4.26 |
| 500 | 0.2 | 0.025 | Skewed | 20 | 1.00 | 5.31 | 4.29 | 4.26 | 4.34 |
| 500 | 0.2 | 0.075 | Even | 4 | 1.00 | 5.86 | 4.68 | 4.66 | 4.32 |
| 500 | 0.2 | 0.075 | Even | 20 | 1.00 | 6.06 | 4.88 | 4.92 | 4.76 |
| 500 | 0.2 | 0.075 | Skewed | 4 | 1.00 | 5.60 | 4.62 | 4.60 | 4.58 |
| 500 | 0.2 | 0.075 | Skewed | 20 | 1.00 | 6.42 | 5.64 | 5.68 | 5.32 |
| 500 | 0.5 | 0.025 | Even | 4 | 1.00 | 7.02 | 5.52 | 5.52 | 5.30 |
| 500 | 0.5 | 0.025 | Even | 20 | 1.00 | 5.68 | 4.44 | 4.48 | 4.24 |
| 500 | 0.5 | 0.025 | Skewed | 4 | 1.00 | 5.84 | 5.17 | 5.06 | 4.88 |
| 500 | 0.5 | 0.025 | Skewed | 20 | 1.00 | 5.52 | 4.83 | 4.82 | 4.68 |
| 500 | 0.5 | 0.075 | Even | 4 | 1.00 | 6.06 | 4.76 | 4.76 | 4.74 |
| 500 | 0.5 | 0.075 | Even | 20 | 1.00 | 6.00 | 4.70 | 4.72 | 4.60 |
| 500 | 0.5 | 0.075 | Skewed | 4 | 1.00 | 6.43 | 5.44 | 5.40 | 5.42 |
| 500 | 0.5 | 0.075 | Skewed | 20 | 1.00 | 5.63 | 4.64 | 4.66 | 4.68 |
| 1000 | 0.2 | 0.025 | Even | 4 | 1.00 | 5.30 | 4.67 | 4.64 | 4.58 |
| 1000 | 0.2 | 0.025 | Even | 20 | 1.00 | 5.64 | 4.88 | 4.86 | 4.80 |
| 1000 | 0.2 | 0.025 | Skewed | 4 | 1.00 | 4.98 | 4.55 | 4.54 | 4.36 |
| 1000 | 0.2 | 0.025 | Skewed | 20 | 1.00 | 5.04 | 4.75 | 4.78 | 4.54 |
| 1000 | 0.2 | 0.075 | Even | 4 | 1.00 | 5.62 | 5.06 | 5.04 | 4.98 |
| 1000 | 0.2 | 0.075 | Even | 20 | 1.00 | 5.88 | 5.16 | 5.18 | 5.10 |
| 1000 | 0.2 | 0.075 | Skewed | 4 | 1.00 | 5.45 | 4.80 | 4.74 | 4.86 |
| 1000 | 0.2 | 0.075 | Skewed | 20 | 1.00 | 5.41 | 4.90 | 4.80 | 4.86 |
| 1000 | 0.5 | 0.025 | Even | 4 | 1.00 | 6.02 | 5.20 | 5.16 | 5.12 |
| 1000 | 0.5 | 0.025 | Even | 20 | 1.00 | 5.48 | 5.06 | 5.06 | 5.04 |
| 1000 | 0.5 | 0.025 | Skewed | 4 | 1.00 | 5.26 | 4.89 | 4.96 | 4.70 |
| 1000 | 0.5 | 0.025 | Skewed | 20 | 1.00 | 5.60 | 5.23 | 5.28 | 5.26 |
| 1000 | 0.5 | 0.075 | Even | 4 | 1.00 | 5.32 | 4.60 | 4.60 | 4.58 |
| 1000 | 0.5 | 0.075 | Even | 20 | 1.00 | 5.26 | 4.76 | 4.76 | 4.70 |
| 1000 | 0.5 | 0.075 | Skewed | 4 | 1.00 | 5.47 | 5.29 | 5.26 | 5.04 |
| 1000 | 0.5 | 0.075 | Skewed | 20 | 1.00 | 5.35 | 5.12 | 5.16 | 4.78 |
| 2000 | 0.2 | 0.025 | Even | 4 | 1.00 | 5.20 | 4.88 | 4.86 | 4.84 |
| 2000 | 0.2 | 0.025 | Even | 20 | 1.00 | 4.96 | 4.72 | 4.70 | 4.68 |
| 2000 | 0.2 | 0.025 | Skewed | 4 | 1.00 | 4.96 | 4.71 | 4.72 | 4.82 |
| 2000 | 0.2 | 0.025 | Skewed | 20 | 1.00 | 5.02 | 4.75 | 4.78 | 4.76 |
| 2000 | 0.2 | 0.075 | Even | 4 | 1.00 | 5.30 | 5.11 | 5.10 | 5.10 |
| 2000 | 0.2 | 0.075 | Even | 20 | 1.00 | 5.80 | 5.51 | 5.52 | 5.52 |
| 2000 | 0.2 | 0.075 | Skewed | 4 | 1.00 | 5.39 | 5.22 | 5.24 | 5.18 |
| 2000 | 0.2 | 0.075 | Skewed | 20 | 1.00 | 5.11 | 4.80 | 4.82 | 4.80 |
| 2000 | 0.5 | 0.025 | Even | 4 | 1.00 | 5.02 | 4.60 | 4.60 | 4.60 |
| 2000 | 0.5 | 0.025 | Even | 20 | 1.00 | 5.34 | 4.76 | 4.78 | 4.74 |
| 2000 | 0.5 | 0.025 | Skewed | 4 | 1.00 | 4.64 | 4.68 | 4.68 | 4.44 |
| 2000 | 0.5 | 0.025 | Skewed | 20 | 1.00 | 5.52 | 5.52 | 5.54 | 5.26 |
| 2000 | 0.5 | 0.075 | Even | 4 | 1.00 | 5.18 | 4.86 | 4.86 | 4.86 |
| 2000 | 0.5 | 0.075 | Even | 20 | 1.00 | 5.06 | 4.86 | 4.86 | 4.86 |
| 2000 | 0.5 | 0.075 | Skewed | 4 | 1.00 | 4.47 | 4.25 | 4.28 | 4.34 |
| 2000 | 0.5 | 0.075 | Skewed | 20 | 1.00 | 5.44 | 5.15 | 5.19 | 5.24 |

**Table 27 – Convergence – 50 centres, true OR=1**

| Sample size | Event rate | ICC | Patient distribution | Block size | True OR | Fixed effects | Random effects | GEE | MH |
| --- | --- | --- | --- | --- | --- | --- | --- | --- | --- |
| 200 | 0.2 | 0.025 | Even | 4 | 1.00 | 100.00 | 99.94 | 100.00 | 99.76 |
| 200 | 0.2 | 0.025 | Even | 20 | 1.00 | 100.00 | 99.92 | 100.00 | 100.00 |
| 200 | 0.2 | 0.025 | Skewed | 4 | 1.00 | 99.96 | 100.00 | 100.00 | 100.00 |
| 200 | 0.2 | 0.025 | Skewed | 20 | 1.00 | 99.98 | 100.00 | 100.00 | 100.00 |
| 200 | 0.2 | 0.075 | Even | 4 | 1.00 | 100.00 | 99.88 | 100.00 | 99.92 |
| 200 | 0.2 | 0.075 | Even | 20 | 1.00 | 100.00 | 99.98 | 100.00 | 100.00 |
| 200 | 0.2 | 0.075 | Skewed | 4 | 1.00 | 100.00 | 99.94 | 100.00 | 100.00 |
| 200 | 0.2 | 0.075 | Skewed | 20 | 1.00 | 99.96 | 100.00 | 100.00 | 100.00 |
| 200 | 0.5 | 0.025 | Even | 4 | 1.00 | 100.00 | 99.74 | 100.00 | 99.88 |
| 200 | 0.5 | 0.025 | Even | 20 | 1.00 | 99.98 | 99.90 | 100.00 | 99.98 |
| 200 | 0.5 | 0.025 | Skewed | 4 | 1.00 | 100.00 | 99.96 | 100.00 | 100.00 |
| 200 | 0.5 | 0.025 | Skewed | 20 | 1.00 | 100.00 | 99.94 | 100.00 | 100.00 |
| 200 | 0.5 | 0.075 | Even | 4 | 1.00 | 99.96 | 99.84 | 100.00 | 99.92 |
| 200 | 0.5 | 0.075 | Even | 20 | 1.00 | 99.96 | 99.86 | 100.00 | 100.00 |
| 200 | 0.5 | 0.075 | Skewed | 4 | 1.00 | 100.00 | 99.98 | 100.00 | 100.00 |
| 200 | 0.5 | 0.075 | Skewed | 20 | 1.00 | 100.00 | 100.00 | 100.00 | 100.00 |
| 500 | 0.2 | 0.025 | Even | 4 | 1.00 | 100.00 | 100.00 | 100.00 | 100.00 |
| 500 | 0.2 | 0.025 | Even | 20 | 1.00 | 100.00 | 99.98 | 100.00 | 100.00 |
| 500 | 0.2 | 0.025 | Skewed | 4 | 1.00 | 99.48 | 99.78 | 100.00 | 100.00 |
| 500 | 0.2 | 0.025 | Skewed | 20 | 1.00 | 99.48 | 99.88 | 100.00 | 100.00 |
| 500 | 0.2 | 0.075 | Even | 4 | 1.00 | 99.98 | 99.98 | 100.00 | 100.00 |
| 500 | 0.2 | 0.075 | Even | 20 | 1.00 | 99.98 | 100.00 | 100.00 | 100.00 |
| 500 | 0.2 | 0.075 | Skewed | 4 | 1.00 | 99.36 | 99.94 | 100.00 | 100.00 |
| 500 | 0.2 | 0.075 | Skewed | 20 | 1.00 | 99.34 | 99.98 | 100.00 | 100.00 |
| 500 | 0.5 | 0.025 | Even | 4 | 1.00 | 100.00 | 99.98 | 100.00 | 100.00 |
| 500 | 0.5 | 0.025 | Even | 20 | 1.00 | 100.00 | 99.94 | 100.00 | 100.00 |
| 500 | 0.5 | 0.025 | Skewed | 4 | 1.00 | 99.98 | 99.88 | 100.00 | 100.00 |
| 500 | 0.5 | 0.025 | Skewed | 20 | 1.00 | 99.98 | 99.78 | 100.00 | 100.00 |
| 500 | 0.5 | 0.075 | Even | 4 | 1.00 | 100.00 | 99.98 | 100.00 | 100.00 |
| 500 | 0.5 | 0.075 | Even | 20 | 1.00 | 100.00 | 100.00 | 100.00 | 100.00 |
| 500 | 0.5 | 0.075 | Skewed | 4 | 1.00 | 99.82 | 99.98 | 100.00 | 100.00 |
| 500 | 0.5 | 0.075 | Skewed | 20 | 1.00 | 99.86 | 99.98 | 100.00 | 100.00 |
| 1000 | 0.2 | 0.025 | Even | 4 | 1.00 | 100.00 | 99.42 | 100.00 | 99.98 |
| 1000 | 0.2 | 0.025 | Even | 20 | 1.00 | 100.00 | 99.56 | 100.00 | 100.00 |
| 1000 | 0.2 | 0.025 | Skewed | 4 | 1.00 | 99.22 | 99.84 | 100.00 | 100.00 |
| 1000 | 0.2 | 0.025 | Skewed | 20 | 1.00 | 99.24 | 99.88 | 100.00 | 100.00 |
| 1000 | 0.2 | 0.075 | Even | 4 | 1.00 | 100.00 | 99.94 | 100.00 | 100.00 |
| 1000 | 0.2 | 0.075 | Even | 20 | 1.00 | 100.00 | 99.98 | 100.00 | 100.00 |
| 1000 | 0.2 | 0.075 | Skewed | 4 | 1.00 | 99.00 | 99.96 | 100.00 | 100.00 |
| 1000 | 0.2 | 0.075 | Skewed | 20 | 1.00 | 99.04 | 99.96 | 100.00 | 100.00 |
| 1000 | 0.5 | 0.025 | Even | 4 | 1.00 | 100.00 | 99.96 | 100.00 | 99.92 |
| 1000 | 0.5 | 0.025 | Even | 20 | 1.00 | 100.00 | 99.92 | 100.00 | 99.98 |
| 1000 | 0.5 | 0.025 | Skewed | 4 | 1.00 | 100.00 | 99.72 | 100.00 | 100.00 |
| 1000 | 0.5 | 0.025 | Skewed | 20 | 1.00 | 100.00 | 99.76 | 100.00 | 100.00 |
| 1000 | 0.5 | 0.075 | Even | 4 | 1.00 | 100.00 | 100.00 | 100.00 | 100.00 |
| 1000 | 0.5 | 0.075 | Even | 20 | 1.00 | 100.00 | 99.98 | 100.00 | 100.00 |
| 1000 | 0.5 | 0.075 | Skewed | 4 | 1.00 | 98.40 | 99.84 | 100.00 | 100.00 |
| 1000 | 0.5 | 0.075 | Skewed | 20 | 1.00 | 98.40 | 99.92 | 100.00 | 100.00 |
| 2000 | 0.2 | 0.025 | Even | 4 | 1.00 | 100.00 | 99.96 | 100.00 | 100.00 |
| 2000 | 0.2 | 0.025 | Even | 20 | 1.00 | 100.00 | 99.94 | 100.00 | 99.98 |
| 2000 | 0.2 | 0.025 | Skewed | 4 | 1.00 | 98.78 | 99.88 | 99.98 | 100.00 |
| 2000 | 0.2 | 0.025 | Skewed | 20 | 1.00 | 98.82 | 99.86 | 99.98 | 100.00 |
| 2000 | 0.2 | 0.075 | Even | 4 | 1.00 | 100.00 | 99.78 | 100.00 | 100.00 |
| 2000 | 0.2 | 0.075 | Even | 20 | 1.00 | 100.00 | 99.88 | 100.00 | 100.00 |
| 2000 | 0.2 | 0.075 | Skewed | 4 | 1.00 | 98.70 | 99.98 | 100.00 | 100.00 |
| 2000 | 0.2 | 0.075 | Skewed | 20 | 1.00 | 98.66 | 99.90 | 99.92 | 100.00 |
| 2000 | 0.5 | 0.025 | Even | 4 | 1.00 | 100.00 | 100.00 | 100.00 | 100.00 |
| 2000 | 0.5 | 0.025 | Even | 20 | 1.00 | 100.00 | 100.00 | 100.00 | 100.00 |
| 2000 | 0.5 | 0.025 | Skewed | 4 | 1.00 | 99.94 | 99.96 | 100.00 | 100.00 |
| 2000 | 0.5 | 0.025 | Skewed | 20 | 1.00 | 99.94 | 99.94 | 100.00 | 100.00 |
| 2000 | 0.5 | 0.075 | Even | 4 | 1.00 | 100.00 | 99.92 | 100.00 | 100.00 |
| 2000 | 0.5 | 0.075 | Even | 20 | 1.00 | 100.00 | 99.96 | 100.00 | 100.00 |
| 2000 | 0.5 | 0.075 | Skewed | 4 | 1.00 | 94.94 | 99.88 | 99.94 | 100.00 |
| 2000 | 0.5 | 0.075 | Skewed | 20 | 1.00 | 94.80 | 99.86 | 99.90 | 100.00 |

**Table 28 – Mean treatment effect – 50 centres, true OR<1**

| Sample size | Event rate | ICC | Patient distribution | Block size | True OR | Fixed effects | Random effects | GEE | MH |
| --- | --- | --- | --- | --- | --- | --- | --- | --- | --- |
| 200 | 0.2 | 0.025 | Even | 4 | 0.25 | 0.14 | 0.22 | 0.22 | 0.23 |
| 200 | 0.2 | 0.025 | Even | 20 | 0.25 | 0.13 | 0.22 | 0.23 | 0.22 |
| 200 | 0.2 | 0.025 | Skewed | 4 | 0.25 | 0.16 | 0.22 | 0.23 | 0.23 |
| 200 | 0.2 | 0.025 | Skewed | 20 | 0.25 | 0.17 | 0.23 | 0.23 | 0.23 |
| 200 | 0.2 | 0.075 | Even | 4 | 0.25 | 0.14 | 0.22 | 0.23 | 0.23 |
| 200 | 0.2 | 0.075 | Even | 20 | 0.25 | 0.13 | 0.22 | 0.24 | 0.22 |
| 200 | 0.2 | 0.075 | Skewed | 4 | 0.25 | 0.16 | 0.23 | 0.24 | 0.22 |
| 200 | 0.2 | 0.075 | Skewed | 20 | 0.25 | 0.16 | 0.23 | 0.24 | 0.22 |
| 200 | 0.5 | 0.025 | Even | 4 | 0.42 | 0.31 | 0.41 | 0.42 | 0.41 |
| 200 | 0.5 | 0.025 | Even | 20 | 0.42 | 0.30 | 0.41 | 0.42 | 0.41 |
| 200 | 0.5 | 0.025 | Skewed | 4 | 0.42 | 0.34 | 0.41 | 0.42 | 0.41 |
| 200 | 0.5 | 0.025 | Skewed | 20 | 0.42 | 0.35 | 0.41 | 0.42 | 0.41 |
| 200 | 0.5 | 0.075 | Even | 4 | 0.42 | 0.31 | 0.41 | 0.44 | 0.41 |
| 200 | 0.5 | 0.075 | Even | 20 | 0.42 | 0.31 | 0.42 | 0.44 | 0.41 |
| 200 | 0.5 | 0.075 | Skewed | 4 | 0.42 | 0.34 | 0.42 | 0.44 | 0.41 |
| 200 | 0.5 | 0.075 | Skewed | 20 | 0.42 | 0.34 | 0.41 | 0.43 | 0.41 |
| 500 | 0.2 | 0.025 | Even | 4 | 0.47 | 0.43 | 0.47 | 0.47 | 0.47 |
| 500 | 0.2 | 0.025 | Even | 20 | 0.47 | 0.43 | 0.47 | 0.48 | 0.47 |
| 500 | 0.2 | 0.025 | Skewed | 4 | 0.47 | 0.45 | 0.47 | 0.48 | 0.47 |
| 500 | 0.2 | 0.025 | Skewed | 20 | 0.47 | 0.45 | 0.47 | 0.48 | 0.47 |
| 500 | 0.2 | 0.075 | Even | 4 | 0.47 | 0.43 | 0.47 | 0.48 | 0.47 |
| 500 | 0.2 | 0.075 | Even | 20 | 0.47 | 0.43 | 0.47 | 0.48 | 0.47 |
| 500 | 0.2 | 0.075 | Skewed | 4 | 0.47 | 0.44 | 0.47 | 0.48 | 0.47 |
| 500 | 0.2 | 0.075 | Skewed | 20 | 0.47 | 0.44 | 0.47 | 0.48 | 0.47 |
| 500 | 0.5 | 0.025 | Even | 4 | 0.59 | 0.56 | 0.59 | 0.60 | 0.59 |
| 500 | 0.5 | 0.025 | Even | 20 | 0.59 | 0.56 | 0.59 | 0.60 | 0.59 |
| 500 | 0.5 | 0.025 | Skewed | 4 | 0.59 | 0.57 | 0.59 | 0.60 | 0.59 |
| 500 | 0.5 | 0.025 | Skewed | 20 | 0.59 | 0.57 | 0.59 | 0.59 | 0.59 |
| 500 | 0.5 | 0.075 | Even | 4 | 0.59 | 0.56 | 0.59 | 0.61 | 0.59 |
| 500 | 0.5 | 0.075 | Even | 20 | 0.59 | 0.56 | 0.59 | 0.61 | 0.59 |
| 500 | 0.5 | 0.075 | Skewed | 4 | 0.59 | 0.56 | 0.59 | 0.61 | 0.59 |
| 500 | 0.5 | 0.075 | Skewed | 20 | 0.59 | 0.57 | 0.59 | 0.61 | 0.59 |
| 1000 | 0.2 | 0.025 | Even | 4 | 0.61 | 0.59 | 0.61 | 0.61 | 0.61 |
| 1000 | 0.2 | 0.025 | Even | 20 | 0.61 | 0.59 | 0.61 | 0.61 | 0.61 |
| 1000 | 0.2 | 0.025 | Skewed | 4 | 0.61 | 0.59 | 0.61 | 0.61 | 0.61 |
| 1000 | 0.2 | 0.025 | Skewed | 20 | 0.61 | 0.59 | 0.61 | 0.61 | 0.61 |
| 1000 | 0.2 | 0.075 | Even | 4 | 0.61 | 0.59 | 0.61 | 0.62 | 0.61 |
| 1000 | 0.2 | 0.075 | Even | 20 | 0.61 | 0.59 | 0.60 | 0.61 | 0.60 |
| 1000 | 0.2 | 0.075 | Skewed | 4 | 0.61 | 0.59 | 0.60 | 0.62 | 0.60 |
| 1000 | 0.2 | 0.075 | Skewed | 20 | 0.61 | 0.60 | 0.61 | 0.62 | 0.61 |
| 1000 | 0.5 | 0.025 | Even | 4 | 0.70 | 0.68 | 0.69 | 0.70 | 0.69 |
| 1000 | 0.5 | 0.025 | Even | 20 | 0.70 | 0.68 | 0.70 | 0.70 | 0.70 |
| 1000 | 0.5 | 0.025 | Skewed | 4 | 0.70 | 0.69 | 0.70 | 0.70 | 0.69 |
| 1000 | 0.5 | 0.025 | Skewed | 20 | 0.70 | 0.68 | 0.69 | 0.70 | 0.69 |
| 1000 | 0.5 | 0.075 | Even | 4 | 0.70 | 0.68 | 0.69 | 0.71 | 0.69 |
| 1000 | 0.5 | 0.075 | Even | 20 | 0.70 | 0.68 | 0.69 | 0.71 | 0.69 |
| 1000 | 0.5 | 0.075 | Skewed | 4 | 0.70 | 0.68 | 0.69 | 0.71 | 0.69 |
| 1000 | 0.5 | 0.075 | Skewed | 20 | 0.70 | 0.69 | 0.70 | 0.71 | 0.69 |
| 2000 | 0.2 | 0.025 | Even | 4 | 0.71 | 0.71 | 0.71 | 0.71 | 0.71 |
| 2000 | 0.2 | 0.025 | Even | 20 | 0.71 | 0.70 | 0.71 | 0.71 | 0.71 |
| 2000 | 0.2 | 0.025 | Skewed | 4 | 0.71 | 0.70 | 0.71 | 0.75 | 0.71 |
| 2000 | 0.2 | 0.025 | Skewed | 20 | 0.71 | 0.71 | 0.71 | 0.71 | 0.71 |
| 2000 | 0.2 | 0.075 | Even | 4 | 0.71 | 0.71 | 0.71 | 0.72 | 0.71 |
| 2000 | 0.2 | 0.075 | Even | 20 | 0.71 | 0.70 | 0.71 | 0.72 | 0.71 |
| 2000 | 0.2 | 0.075 | Skewed | 4 | 0.71 | 0.70 | 0.71 | 0.71 | 0.71 |
| 2000 | 0.2 | 0.075 | Skewed | 20 | 0.71 | 0.70 | 0.71 | 0.73 | 0.71 |
| 2000 | 0.5 | 0.025 | Even | 4 | 0.77 | 0.76 | 0.77 | 0.77 | 0.77 |
| 2000 | 0.5 | 0.025 | Even | 20 | 0.77 | 0.77 | 0.77 | 0.78 | 0.77 |
| 2000 | 0.5 | 0.025 | Skewed | 4 | 0.77 | 0.77 | 0.77 | 0.77 | 0.77 |
| 2000 | 0.5 | 0.025 | Skewed | 20 | 0.77 | 0.77 | 0.77 | 0.77 | 0.77 |
| 2000 | 0.5 | 0.075 | Even | 4 | 0.77 | 0.76 | 0.77 | 0.78 | 0.77 |
| 2000 | 0.5 | 0.075 | Even | 20 | 0.77 | 0.76 | 0.77 | 0.78 | 0.77 |
| 2000 | 0.5 | 0.075 | Skewed | 4 | 0.77 | 0.77 | 0.77 | 0.77 | 0.77 |
| 2000 | 0.5 | 0.075 | Skewed | 20 | 0.77 | 0.77 | 0.77 | 0.77 | 0.77 |

**Table 29 – Power – 50 centres, true OR<1**

| Sample size | Event rate | ICC | Patient distribution | Block size | True OR | Fixed effects | Random effects | GEE | MH |
| --- | --- | --- | --- | --- | --- | --- | --- | --- | --- |
| 200 | 0.2 | 0.025 | Even | 4 | 0.25 | 92.19 | 86.79 | 86.40 | 86.00 |
| 200 | 0.2 | 0.025 | Even | 20 | 0.25 | 84.50 | 86.13 | 86.37 | 74.37 |
| 200 | 0.2 | 0.025 | Skewed | 4 | 0.25 | 85.79 | 86.31 | 86.19 | 80.30 |
| 200 | 0.2 | 0.025 | Skewed | 20 | 0.25 | 81.58 | 85.59 | 85.73 | 74.60 |
| 200 | 0.2 | 0.075 | Even | 4 | 0.25 | 92.56 | 87.68 | 87.44 | 86.86 |
| 200 | 0.2 | 0.075 | Even | 20 | 0.25 | 86.07 | 86.18 | 86.54 | 75.98 |
| 200 | 0.2 | 0.075 | Skewed | 4 | 0.25 | 87.75 | 87.86 | 87.64 | 81.70 |
| 200 | 0.2 | 0.075 | Skewed | 20 | 0.25 | 83.53 | 87.10 | 86.97 | 77.24 |
| 200 | 0.5 | 0.025 | Even | 4 | 0.42 | 89.15 | 84.08 | 83.72 | 83.08 |
| 200 | 0.5 | 0.025 | Even | 20 | 0.42 | 82.18 | 83.17 | 83.66 | 73.68 |
| 200 | 0.5 | 0.025 | Skewed | 4 | 0.42 | 83.65 | 82.89 | 83.02 | 77.14 |
| 200 | 0.5 | 0.025 | Skewed | 20 | 0.42 | 79.79 | 83.72 | 83.70 | 73.58 |
| 200 | 0.5 | 0.075 | Even | 4 | 0.42 | 88.46 | 82.79 | 82.80 | 82.12 |
| 200 | 0.5 | 0.075 | Even | 20 | 0.42 | 79.69 | 80.08 | 80.48 | 70.74 |
| 200 | 0.5 | 0.075 | Skewed | 4 | 0.42 | 82.01 | 81.12 | 81.34 | 75.64 |
| 200 | 0.5 | 0.075 | Skewed | 20 | 0.42 | 79.07 | 82.06 | 82.38 | 72.38 |
| 500 | 0.2 | 0.025 | Even | 4 | 0.47 | 85.24 | 83.56 | 83.46 | 82.80 |
| 500 | 0.2 | 0.025 | Even | 20 | 0.47 | 83.62 | 83.43 | 83.36 | 80.64 |
| 500 | 0.2 | 0.025 | Skewed | 4 | 0.47 | 83.79 | 83.68 | 83.46 | 81.40 |
| 500 | 0.2 | 0.025 | Skewed | 20 | 0.47 | 82.78 | 82.49 | 82.38 | 80.00 |
| 500 | 0.2 | 0.075 | Even | 4 | 0.47 | 86.88 | 85.19 | 85.16 | 84.32 |
| 500 | 0.2 | 0.075 | Even | 20 | 0.47 | 85.06 | 84.07 | 84.12 | 82.52 |
| 500 | 0.2 | 0.075 | Skewed | 4 | 0.47 | 84.09 | 83.69 | 83.66 | 82.02 |
| 500 | 0.2 | 0.075 | Skewed | 20 | 0.47 | 83.97 | 83.81 | 83.76 | 81.58 |
| 500 | 0.5 | 0.025 | Even | 4 | 0.59 | 83.34 | 81.34 | 81.30 | 80.36 |
| 500 | 0.5 | 0.025 | Even | 20 | 0.59 | 81.76 | 80.33 | 80.46 | 78.60 |
| 500 | 0.5 | 0.025 | Skewed | 4 | 0.59 | 82.19 | 81.56 | 81.62 | 79.88 |
| 500 | 0.5 | 0.025 | Skewed | 20 | 0.59 | 81.42 | 82.58 | 82.56 | 79.30 |
| 500 | 0.5 | 0.075 | Even | 4 | 0.59 | 82.86 | 80.52 | 80.52 | 79.64 |
| 500 | 0.5 | 0.075 | Even | 20 | 0.59 | 80.80 | 79.68 | 79.88 | 77.92 |
| 500 | 0.5 | 0.075 | Skewed | 4 | 0.59 | 80.43 | 79.90 | 79.82 | 77.74 |
| 500 | 0.5 | 0.075 | Skewed | 20 | 0.59 | 80.27 | 80.20 | 80.44 | 77.84 |
| 1000 | 0.2 | 0.025 | Even | 4 | 0.61 | 84.04 | 82.66 | 82.62 | 82.52 |
| 1000 | 0.2 | 0.025 | Even | 20 | 0.61 | 84.22 | 82.95 | 82.90 | 82.82 |
| 1000 | 0.2 | 0.025 | Skewed | 4 | 0.61 | 83.74 | 83.33 | 83.36 | 82.24 |
| 1000 | 0.2 | 0.025 | Skewed | 20 | 0.61 | 83.20 | 83.92 | 83.82 | 82.40 |
| 1000 | 0.2 | 0.075 | Even | 4 | 0.61 | 84.40 | 83.06 | 83.04 | 83.04 |
| 1000 | 0.2 | 0.075 | Even | 20 | 0.61 | 84.90 | 83.74 | 83.72 | 83.68 |
| 1000 | 0.2 | 0.075 | Skewed | 4 | 0.61 | 84.49 | 84.26 | 83.86 | 83.40 |
| 1000 | 0.2 | 0.075 | Skewed | 20 | 0.61 | 82.06 | 82.55 | 82.42 | 81.26 |
| 1000 | 0.5 | 0.025 | Even | 4 | 0.70 | 82.52 | 81.14 | 81.12 | 81.06 |
| 1000 | 0.5 | 0.025 | Even | 20 | 0.70 | 82.20 | 80.95 | 80.90 | 80.86 |
| 1000 | 0.5 | 0.025 | Skewed | 4 | 0.70 | 80.29 | 79.90 | 79.98 | 79.10 |
| 1000 | 0.5 | 0.025 | Skewed | 20 | 0.70 | 80.80 | 81.21 | 81.22 | 79.98 |
| 1000 | 0.5 | 0.075 | Even | 4 | 0.70 | 80.82 | 79.55 | 79.56 | 79.42 |
| 1000 | 0.5 | 0.075 | Even | 20 | 0.70 | 80.20 | 78.64 | 78.70 | 78.58 |
| 1000 | 0.5 | 0.075 | Skewed | 4 | 0.70 | 79.36 | 78.85 | 78.86 | 78.12 |
| 1000 | 0.5 | 0.075 | Skewed | 20 | 0.70 | 78.36 | 78.60 | 78.70 | 77.32 |
| 2000 | 0.2 | 0.025 | Even | 4 | 0.71 | 83.72 | 83.08 | 83.04 | 82.98 |
| 2000 | 0.2 | 0.025 | Even | 20 | 0.71 | 83.52 | 82.80 | 82.80 | 82.78 |
| 2000 | 0.2 | 0.025 | Skewed | 4 | 0.71 | 83.18 | 82.90 | 82.91 | 82.40 |
| 2000 | 0.2 | 0.025 | Skewed | 20 | 0.71 | 82.46 | 82.70 | 82.70 | 81.92 |
| 2000 | 0.2 | 0.075 | Even | 4 | 0.71 | 83.24 | 82.55 | 82.52 | 82.54 |
| 2000 | 0.2 | 0.075 | Even | 20 | 0.71 | 84.22 | 83.63 | 83.54 | 83.56 |
| 2000 | 0.2 | 0.075 | Skewed | 4 | 0.71 | 82.49 | 82.52 | 82.52 | 81.90 |
| 2000 | 0.2 | 0.075 | Skewed | 20 | 0.71 | 82.39 | 82.15 | 82.13 | 81.72 |
| 2000 | 0.5 | 0.025 | Even | 4 | 0.77 | 82.58 | 81.49 | 81.48 | 81.44 |
| 2000 | 0.5 | 0.025 | Even | 20 | 0.77 | 82.78 | 81.55 | 81.56 | 81.52 |
| 2000 | 0.5 | 0.025 | Skewed | 4 | 0.77 | 82.53 | 82.46 | 82.46 | 82.18 |
| 2000 | 0.5 | 0.025 | Skewed | 20 | 0.77 | 81.41 | 81.55 | 81.54 | 81.06 |
| 2000 | 0.5 | 0.075 | Even | 4 | 0.77 | 81.36 | 80.79 | 80.80 | 80.72 |
| 2000 | 0.5 | 0.075 | Even | 20 | 0.77 | 82.02 | 81.44 | 81.44 | 81.40 |
| 2000 | 0.5 | 0.075 | Skewed | 4 | 0.77 | 80.26 | 80.65 | 80.64 | 80.16 |
| 2000 | 0.5 | 0.075 | Skewed | 20 | 0.77 | 80.61 | 80.54 | 80.54 | 80.14 |

**Table 30 – Convergence – 50 centres, true OR<1**

| Sample size | Event rate | ICC | Patient distribution | Block size | True OR | Fixed effects | Random effects | GEE | MH |
| --- | --- | --- | --- | --- | --- | --- | --- | --- | --- |
| 200 | 0.2 | 0.025 | Even | 4 | 0.25 | 99.60 | 99.80 | 100.00 | 100.00 |
| 200 | 0.2 | 0.025 | Even | 20 | 0.25 | 99.10 | 99.76 | 99.94 | 99.98 |
| 200 | 0.2 | 0.025 | Skewed | 4 | 0.25 | 99.08 | 99.90 | 99.96 | 100.00 |
| 200 | 0.2 | 0.025 | Skewed | 20 | 0.25 | 98.70 | 99.76 | 99.92 | 100.00 |
| 200 | 0.2 | 0.075 | Even | 4 | 0.25 | 99.48 | 99.82 | 100.00 | 100.00 |
| 200 | 0.2 | 0.075 | Even | 20 | 0.25 | 99.52 | 99.82 | 100.00 | 100.00 |
| 200 | 0.2 | 0.075 | Skewed | 4 | 0.25 | 99.14 | 99.84 | 99.96 | 100.00 |
| 200 | 0.2 | 0.075 | Skewed | 20 | 0.25 | 98.72 | 99.84 | 99.96 | 100.00 |
| 200 | 0.5 | 0.025 | Even | 4 | 0.42 | 99.92 | 99.88 | 100.00 | 100.00 |
| 200 | 0.5 | 0.025 | Even | 20 | 0.42 | 99.88 | 99.96 | 100.00 | 100.00 |
| 200 | 0.5 | 0.025 | Skewed | 4 | 0.42 | 99.96 | 99.96 | 100.00 | 100.00 |
| 200 | 0.5 | 0.025 | Skewed | 20 | 0.42 | 99.96 | 99.98 | 100.00 | 100.00 |
| 200 | 0.5 | 0.075 | Even | 4 | 0.42 | 100.00 | 99.92 | 100.00 | 100.00 |
| 200 | 0.5 | 0.075 | Even | 20 | 0.42 | 99.94 | 99.98 | 100.00 | 100.00 |
| 200 | 0.5 | 0.075 | Skewed | 4 | 0.42 | 99.92 | 99.98 | 100.00 | 100.00 |
| 200 | 0.5 | 0.075 | Skewed | 20 | 0.42 | 99.94 | 100.00 | 100.00 | 100.00 |
| 500 | 0.2 | 0.025 | Even | 4 | 0.47 | 100.00 | 99.98 | 100.00 | 100.00 |
| 500 | 0.2 | 0.025 | Even | 20 | 0.47 | 100.00 | 99.94 | 100.00 | 100.00 |
| 500 | 0.2 | 0.025 | Skewed | 4 | 0.47 | 97.70 | 99.90 | 100.00 | 100.00 |
| 500 | 0.2 | 0.025 | Skewed | 20 | 0.47 | 97.44 | 99.94 | 100.00 | 100.00 |
| 500 | 0.2 | 0.075 | Even | 4 | 0.47 | 100.00 | 99.96 | 100.00 | 100.00 |
| 500 | 0.2 | 0.075 | Even | 20 | 0.47 | 100.00 | 99.94 | 100.00 | 100.00 |
| 500 | 0.2 | 0.075 | Skewed | 4 | 0.47 | 98.56 | 99.96 | 100.00 | 100.00 |
| 500 | 0.2 | 0.075 | Skewed | 20 | 0.47 | 98.18 | 99.92 | 100.00 | 100.00 |
| 500 | 0.5 | 0.025 | Even | 4 | 0.59 | 100.00 | 99.88 | 100.00 | 100.00 |
| 500 | 0.5 | 0.025 | Even | 20 | 0.59 | 100.00 | 99.96 | 100.00 | 100.00 |
| 500 | 0.5 | 0.025 | Skewed | 4 | 0.59 | 99.82 | 99.78 | 100.00 | 100.00 |
| 500 | 0.5 | 0.025 | Skewed | 20 | 0.59 | 99.78 | 99.86 | 100.00 | 100.00 |
| 500 | 0.5 | 0.075 | Even | 4 | 0.59 | 100.00 | 100.00 | 100.00 | 100.00 |
| 500 | 0.5 | 0.075 | Even | 20 | 0.59 | 100.00 | 100.00 | 100.00 | 100.00 |
| 500 | 0.5 | 0.075 | Skewed | 4 | 0.59 | 99.52 | 100.00 | 100.00 | 100.00 |
| 500 | 0.5 | 0.075 | Skewed | 20 | 0.59 | 99.62 | 99.92 | 100.00 | 100.00 |
| 1000 | 0.2 | 0.025 | Even | 4 | 0.61 | 100.00 | 99.66 | 100.00 | 100.00 |
| 1000 | 0.2 | 0.025 | Even | 20 | 0.61 | 100.00 | 99.70 | 100.00 | 100.00 |
| 1000 | 0.2 | 0.025 | Skewed | 4 | 0.61 | 95.72 | 99.82 | 100.00 | 100.00 |
| 1000 | 0.2 | 0.025 | Skewed | 20 | 0.61 | 95.58 | 99.74 | 100.00 | 100.00 |
| 1000 | 0.2 | 0.075 | Even | 4 | 0.61 | 99.98 | 100.00 | 100.00 | 100.00 |
| 1000 | 0.2 | 0.075 | Even | 20 | 0.61 | 100.00 | 99.98 | 100.00 | 100.00 |
| 1000 | 0.2 | 0.075 | Skewed | 4 | 0.61 | 97.60 | 99.98 | 100.00 | 100.00 |
| 1000 | 0.2 | 0.075 | Skewed | 20 | 0.61 | 97.42 | 99.92 | 100.00 | 100.00 |
| 1000 | 0.5 | 0.025 | Even | 4 | 0.70 | 100.00 | 99.88 | 100.00 | 100.00 |
| 1000 | 0.5 | 0.025 | Even | 20 | 0.70 | 100.00 | 99.86 | 100.00 | 100.00 |
| 1000 | 0.5 | 0.025 | Skewed | 4 | 0.70 | 99.76 | 99.92 | 100.00 | 100.00 |
| 1000 | 0.5 | 0.025 | Skewed | 20 | 0.70 | 99.80 | 99.76 | 100.00 | 100.00 |
| 1000 | 0.5 | 0.075 | Even | 4 | 0.70 | 100.00 | 99.96 | 100.00 | 100.00 |
| 1000 | 0.5 | 0.075 | Even | 20 | 0.70 | 100.00 | 99.98 | 100.00 | 100.00 |
| 1000 | 0.5 | 0.075 | Skewed | 4 | 0.70 | 97.30 | 99.94 | 100.00 | 100.00 |
| 1000 | 0.5 | 0.075 | Skewed | 20 | 0.70 | 97.32 | 99.90 | 100.00 | 100.00 |
| 2000 | 0.2 | 0.025 | Even | 4 | 0.71 | 100.00 | 99.90 | 100.00 | 100.00 |
| 2000 | 0.2 | 0.025 | Even | 20 | 0.71 | 100.00 | 99.90 | 100.00 | 100.00 |
| 2000 | 0.2 | 0.025 | Skewed | 4 | 0.71 | 96.18 | 99.90 | 99.92 | 100.00 |
| 2000 | 0.2 | 0.025 | Skewed | 20 | 0.71 | 95.78 | 99.98 | 99.98 | 100.00 |
| 2000 | 0.2 | 0.075 | Even | 4 | 0.71 | 100.00 | 99.82 | 100.00 | 100.00 |
| 2000 | 0.2 | 0.075 | Even | 20 | 0.71 | 100.00 | 99.84 | 100.00 | 100.00 |
| 2000 | 0.2 | 0.075 | Skewed | 4 | 0.71 | 96.98 | 99.98 | 99.98 | 100.00 |
| 2000 | 0.2 | 0.075 | Skewed | 20 | 0.71 | 97.32 | 99.92 | 99.94 | 100.00 |
| 2000 | 0.5 | 0.025 | Even | 4 | 0.77 | 100.00 | 99.96 | 100.00 | 100.00 |
| 2000 | 0.5 | 0.025 | Even | 20 | 0.77 | 100.00 | 99.96 | 100.00 | 100.00 |
| 2000 | 0.5 | 0.025 | Skewed | 4 | 0.77 | 99.92 | 99.98 | 100.00 | 100.00 |
| 2000 | 0.5 | 0.025 | Skewed | 20 | 0.77 | 99.82 | 99.96 | 100.00 | 100.00 |
| 2000 | 0.5 | 0.075 | Even | 4 | 0.77 | 100.00 | 99.96 | 100.00 | 100.00 |
| 2000 | 0.5 | 0.075 | Even | 20 | 0.77 | 100.00 | 100.00 | 100.00 | 100.00 |
| 2000 | 0.5 | 0.075 | Skewed | 4 | 0.77 | 94.62 | 99.84 | 99.88 | 100.00 |
| 2000 | 0.5 | 0.075 | Skewed | 20 | 0.77 | 94.46 | 99.98 | 99.98 | 100.00 |

**Table 31 – Mean treatment effect – 100 centres, true OR=1**

| Sample size | Event rate | ICC | Patient distribution | Block size | True OR | Fixed effects | Random effects | GEE | MH |
| --- | --- | --- | --- | --- | --- | --- | --- | --- | --- |
| 200 | 0.2 | 0.025 | Even | 4 | 1 | 0.99 | 1.00 | 1.00 | 1.00 |
| 200 | 0.2 | 0.025 | Even | 20 | 1 | 1.02 | 1.01 | 1.01 | 1.01 |
| 200 | 0.2 | 0.025 | Skewed | 4 | 1 | 0.99 | 1.00 | 1.00 | 1.00 |
| 200 | 0.2 | 0.025 | Skewed | 20 | 1 | 0.98 | 0.99 | 0.99 | 0.99 |
| 200 | 0.2 | 0.075 | Even | 4 | 1 | 1.00 | 1.00 | 1.00 | 1.00 |
| 200 | 0.2 | 0.075 | Even | 20 | 1 | 1.01 | 1.00 | 1.00 | 1.01 |
| 200 | 0.2 | 0.075 | Skewed | 4 | 1 | 1.01 | 1.00 | 1.00 | 1.01 |
| 200 | 0.2 | 0.075 | Skewed | 20 | 1 | 0.99 | 1.00 | 1.00 | 0.99 |
| 200 | 0.5 | 0.025 | Even | 4 | 1 | 1.01 | 1.00 | 1.00 | 1.01 |
| 200 | 0.5 | 0.025 | Even | 20 | 1 | 1.00 | 1.01 | 1.01 | 1.00 |
| 200 | 0.5 | 0.025 | Skewed | 4 | 1 | 1.01 | 1.00 | 1.00 | 1.01 |
| 200 | 0.5 | 0.025 | Skewed | 20 | 1 | 1.00 | 1.00 | 1.00 | 1.00 |
| 200 | 0.5 | 0.075 | Even | 4 | 1 | 1.01 | 1.00 | 1.00 | 1.00 |
| 200 | 0.5 | 0.075 | Even | 20 | 1 | 1.01 | 1.00 | 1.00 | 1.00 |
| 200 | 0.5 | 0.075 | Skewed | 4 | 1 | 1.00 | 1.00 | 1.00 | 1.00 |
| 200 | 0.5 | 0.075 | Skewed | 20 | 1 | 1.00 | 1.00 | 1.00 | 1.00 |
| 500 | 0.2 | 0.025 | Even | 4 | 1 | 0.99 | 1.00 | 1.00 | 1.00 |
| 500 | 0.2 | 0.025 | Even | 20 | 1 | 0.99 | 0.99 | 0.99 | 0.99 |
| 500 | 0.2 | 0.025 | Skewed | 4 | 1 | 1.00 | 1.00 | 1.00 | 1.00 |
| 500 | 0.2 | 0.025 | Skewed | 20 | 1 | 1.00 | 1.00 | 1.00 | 1.00 |
| 500 | 0.2 | 0.075 | Even | 4 | 1 | 0.99 | 0.99 | 0.99 | 0.99 |
| 500 | 0.2 | 0.075 | Even | 20 | 1 | 0.99 | 0.99 | 1.00 | 0.99 |
| 500 | 0.2 | 0.075 | Skewed | 4 | 1 | 1.00 | 1.00 | 1.00 | 1.00 |
| 500 | 0.2 | 0.075 | Skewed | 20 | 1 | 1.00 | 1.00 | 1.00 | 1.00 |
| 500 | 0.5 | 0.025 | Even | 4 | 1 | 1.00 | 1.00 | 1.00 | 1.00 |
| 500 | 0.5 | 0.025 | Even | 20 | 1 | 1.00 | 1.00 | 1.00 | 1.00 |
| 500 | 0.5 | 0.025 | Skewed | 4 | 1 | 1.00 | 1.00 | 1.00 | 1.00 |
| 500 | 0.5 | 0.025 | Skewed | 20 | 1 | 1.00 | 1.00 | 1.00 | 1.00 |
| 500 | 0.5 | 0.075 | Even | 4 | 1 | 0.99 | 0.99 | 0.99 | 0.99 |
| 500 | 0.5 | 0.075 | Even | 20 | 1 | 1.00 | 1.00 | 1.00 | 1.00 |
| 500 | 0.5 | 0.075 | Skewed | 4 | 1 | 1.00 | 1.00 | 1.00 | 1.00 |
| 500 | 0.5 | 0.075 | Skewed | 20 | 1 | 1.00 | 1.00 | 1.00 | 1.00 |
| 1000 | 0.2 | 0.025 | Even | 4 | 1 | 1.00 | 1.00 | 1.00 | 1.00 |
| 1000 | 0.2 | 0.025 | Even | 20 | 1 | 1.00 | 1.00 | 1.00 | 1.00 |
| 1000 | 0.2 | 0.025 | Skewed | 4 | 1 | 1.00 | 1.00 | 1.00 | 1.00 |
| 1000 | 0.2 | 0.025 | Skewed | 20 | 1 | 1.00 | 1.00 | 1.00 | 1.00 |
| 1000 | 0.2 | 0.075 | Even | 4 | 1 | 1.00 | 1.00 | 1.00 | 1.00 |
| 1000 | 0.2 | 0.075 | Even | 20 | 1 | 1.00 | 1.00 | 1.00 | 1.00 |
| 1000 | 0.2 | 0.075 | Skewed | 4 | 1 | 1.00 | 1.00 | 1.00 | 1.00 |
| 1000 | 0.2 | 0.075 | Skewed | 20 | 1 | 1.00 | 1.00 | 1.00 | 1.00 |
| 1000 | 0.5 | 0.025 | Even | 4 | 1 | 1.00 | 1.00 | 1.00 | 1.00 |
| 1000 | 0.5 | 0.025 | Even | 20 | 1 | 1.00 | 1.00 | 1.00 | 1.00 |
| 1000 | 0.5 | 0.025 | Skewed | 4 | 1 | 1.00 | 1.00 | 1.00 | 1.00 |
| 1000 | 0.5 | 0.025 | Skewed | 20 | 1 | 1.00 | 1.00 | 1.00 | 1.00 |
| 1000 | 0.5 | 0.075 | Even | 4 | 1 | 1.00 | 1.00 | 1.00 | 1.00 |
| 1000 | 0.5 | 0.075 | Even | 20 | 1 | 1.00 | 1.00 | 1.00 | 1.00 |
| 1000 | 0.5 | 0.075 | Skewed | 4 | 1 | 1.00 | 1.00 | 1.00 | 1.00 |
| 1000 | 0.5 | 0.075 | Skewed | 20 | 1 | 1.00 | 1.00 | 1.00 | 1.00 |
| 2000 | 0.2 | 0.025 | Even | 4 | 1 | 1.00 | 1.00 | 1.00 | 1.00 |
| 2000 | 0.2 | 0.025 | Even | 20 | 1 | 1.00 | 1.00 | 1.00 | 1.00 |
| 2000 | 0.2 | 0.025 | Skewed | 4 | 1 | 1.00 | 1.00 | 1.01 | 1.00 |
| 2000 | 0.2 | 0.025 | Skewed | 20 | 1 | 1.00 | 1.00 | 1.01 | 1.00 |
| 2000 | 0.2 | 0.075 | Even | 4 | 1 | 1.00 | 1.00 | 1.00 | 1.00 |
| 2000 | 0.2 | 0.075 | Even | 20 | 1 | 1.00 | 1.00 | 1.00 | 1.00 |
| 2000 | 0.2 | 0.075 | Skewed | 4 | 1 | 1.00 | 1.00 | 1.00 | 1.00 |
| 2000 | 0.2 | 0.075 | Skewed | 20 | 1 | 1.00 | 1.00 | 1.00 | 1.00 |
| 2000 | 0.5 | 0.025 | Even | 4 | 1 | 1.00 | 1.00 | 1.00 | 1.00 |
| 2000 | 0.5 | 0.025 | Even | 20 | 1 | 1.00 | 1.00 | 1.00 | 1.00 |
| 2000 | 0.5 | 0.025 | Skewed | 4 | 1 | 1.00 | 1.00 | 1.13 | 1.00 |
| 2000 | 0.5 | 0.025 | Skewed | 20 | 1 | 1.00 | 1.00 | 1.00 | 1.00 |
| 2000 | 0.5 | 0.075 | Even | 4 | 1 | 1.00 | 1.00 | 1.00 | 1.00 |
| 2000 | 0.5 | 0.075 | Even | 20 | 1 | 1.00 | 1.00 | 1.00 | 1.00 |
| 2000 | 0.5 | 0.075 | Skewed | 4 | 1 | 1.00 | 1.00 | 1.00 | 1.00 |
| 2000 | 0.5 | 0.075 | Skewed | 20 | 1 | 1.00 | 1.00 | 1.01 | 1.00 |

**Table 32 – Type I error rates – 100 centres, true OR=1**

| Sample size | Event rate | ICC | Patient distribution | Block size | True OR | Fixed effects | Random effects | GEE | MH |
| --- | --- | --- | --- | --- | --- | --- | --- | --- | --- |
| 200 | 0.2 | 0.025 | Even | 4 | 1 | 15.12 | 4.30 | 4.74 | 3.58 |
| 200 | 0.2 | 0.025 | Even | 20 | 1 | 16.00 | 4.18 | 4.76 | 3.56 |
| 200 | 0.2 | 0.025 | Skewed | 4 | 1 | 9.62 | 4.66 | 4.86 | 4.30 |
| 200 | 0.2 | 0.025 | Skewed | 20 | 1 | 8.76 | 4.84 | 5.04 | 3.96 |
| 200 | 0.2 | 0.075 | Even | 4 | 1 | 16.58 | 5.31 | 5.88 | 4.60 |
| 200 | 0.2 | 0.075 | Even | 20 | 1 | 15.92 | 4.28 | 4.80 | 4.00 |
| 200 | 0.2 | 0.075 | Skewed | 4 | 1 | 9.84 | 4.77 | 4.96 | 4.54 |
| 200 | 0.2 | 0.075 | Skewed | 20 | 1 | 8.79 | 4.82 | 5.16 | 3.98 |
| 200 | 0.5 | 0.025 | Even | 4 | 1 | 16.28 | 4.65 | 4.92 | 4.56 |
| 200 | 0.5 | 0.025 | Even | 20 | 1 | 15.76 | 4.51 | 4.94 | 4.30 |
| 200 | 0.5 | 0.025 | Skewed | 4 | 1 | 9.39 | 5.20 | 5.24 | 4.40 |
| 200 | 0.5 | 0.025 | Skewed | 20 | 1 | 9.32 | 5.03 | 5.20 | 4.18 |
| 200 | 0.5 | 0.075 | Even | 4 | 1 | 15.26 | 4.47 | 4.90 | 4.44 |
| 200 | 0.5 | 0.075 | Even | 20 | 1 | 15.85 | 4.74 | 5.60 | 5.00 |
| 200 | 0.5 | 0.075 | Skewed | 4 | 1 | 9.54 | 4.76 | 4.98 | 4.88 |
| 200 | 0.5 | 0.075 | Skewed | 20 | 1 | 8.86 | 4.82 | 5.18 | 4.18 |
| 500 | 0.2 | 0.025 | Even | 4 | 1 | 7.48 | 4.75 | 4.76 | 4.56 |
| 500 | 0.2 | 0.025 | Even | 20 | 1 | 7.86 | 4.77 | 4.82 | 4.96 |
| 500 | 0.2 | 0.025 | Skewed | 4 | 1 | 6.71 | 5.00 | 5.08 | 4.98 |
| 500 | 0.2 | 0.025 | Skewed | 20 | 1 | 6.81 | 5.06 | 5.22 | 5.14 |
| 500 | 0.2 | 0.075 | Even | 4 | 1 | 7.10 | 4.66 | 4.68 | 4.28 |
| 500 | 0.2 | 0.075 | Even | 20 | 1 | 7.82 | 4.68 | 4.88 | 4.74 |
| 500 | 0.2 | 0.075 | Skewed | 4 | 1 | 6.91 | 4.72 | 4.70 | 5.08 |
| 500 | 0.2 | 0.075 | Skewed | 20 | 1 | 6.55 | 5.44 | 5.56 | 5.06 |
| 500 | 0.5 | 0.025 | Even | 4 | 1 | 7.92 | 4.90 | 4.80 | 4.88 |
| 500 | 0.5 | 0.025 | Even | 20 | 1 | 7.52 | 4.92 | 5.10 | 4.24 |
| 500 | 0.5 | 0.025 | Skewed | 4 | 1 | 6.02 | 4.98 | 5.00 | 4.28 |
| 500 | 0.5 | 0.025 | Skewed | 20 | 1 | 6.72 | 5.30 | 5.46 | 4.92 |
| 500 | 0.5 | 0.075 | Even | 4 | 1 | 7.70 | 4.98 | 5.06 | 4.82 |
| 500 | 0.5 | 0.075 | Even | 20 | 1 | 7.94 | 4.72 | 4.78 | 5.12 |
| 500 | 0.5 | 0.075 | Skewed | 4 | 1 | 6.90 | 4.94 | 4.96 | 4.98 |
| 500 | 0.5 | 0.075 | Skewed | 20 | 1 | 6.08 | 5.32 | 5.28 | 4.70 |
| 1000 | 0.2 | 0.025 | Even | 4 | 1 | 6.26 | 5.24 | 5.26 | 5.08 |
| 1000 | 0.2 | 0.025 | Even | 20 | 1 | 6.70 | 5.08 | 5.10 | 5.08 |
| 1000 | 0.2 | 0.025 | Skewed | 4 | 1 | 5.18 | 4.58 | 4.58 | 4.22 |
| 1000 | 0.2 | 0.025 | Skewed | 20 | 1 | 5.38 | 5.12 | 5.20 | 4.70 |
| 1000 | 0.2 | 0.075 | Even | 4 | 1 | 6.30 | 5.00 | 4.98 | 5.04 |
| 1000 | 0.2 | 0.075 | Even | 20 | 1 | 6.64 | 4.70 | 4.72 | 5.02 |
| 1000 | 0.2 | 0.075 | Skewed | 4 | 1 | 5.76 | 4.90 | 4.96 | 5.04 |
| 1000 | 0.2 | 0.075 | Skewed | 20 | 1 | 5.64 | 4.86 | 4.88 | 4.84 |
| 1000 | 0.5 | 0.025 | Even | 4 | 1 | 5.66 | 4.70 | 4.72 | 4.70 |
| 1000 | 0.5 | 0.025 | Even | 20 | 1 | 6.04 | 4.48 | 4.52 | 4.46 |
| 1000 | 0.5 | 0.025 | Skewed | 4 | 1 | 6.54 | 5.82 | 5.78 | 5.56 |
| 1000 | 0.5 | 0.025 | Skewed | 20 | 1 | 5.82 | 5.17 | 5.16 | 5.06 |
| 1000 | 0.5 | 0.075 | Even | 4 | 1 | 6.76 | 5.56 | 5.58 | 5.42 |
| 1000 | 0.5 | 0.075 | Even | 20 | 1 | 6.06 | 4.64 | 4.66 | 4.70 |
| 1000 | 0.5 | 0.075 | Skewed | 4 | 1 | 5.64 | 4.90 | 4.96 | 4.86 |
| 1000 | 0.5 | 0.075 | Skewed | 20 | 1 | 5.52 | 4.90 | 4.96 | 4.74 |
| 2000 | 0.2 | 0.025 | Even | 4 | 1 | 5.46 | 4.77 | 4.76 | 4.74 |
| 2000 | 0.2 | 0.025 | Even | 20 | 1 | 5.32 | 4.62 | 4.64 | 4.64 |
| 2000 | 0.2 | 0.025 | Skewed | 4 | 1 | 5.58 | 4.97 | 4.96 | 5.18 |
| 2000 | 0.2 | 0.025 | Skewed | 20 | 1 | 5.30 | 4.87 | 4.86 | 4.92 |
| 2000 | 0.2 | 0.075 | Even | 4 | 1 | 5.50 | 4.82 | 4.80 | 4.80 |
| 2000 | 0.2 | 0.075 | Even | 20 | 1 | 5.34 | 4.78 | 4.76 | 4.76 |
| 2000 | 0.2 | 0.075 | Skewed | 4 | 1 | 5.40 | 5.04 | 5.04 | 5.10 |
| 2000 | 0.2 | 0.075 | Skewed | 20 | 1 | 5.72 | 5.38 | 5.38 | 5.24 |
| 2000 | 0.5 | 0.025 | Even | 4 | 1 | 5.38 | 4.82 | 4.84 | 4.84 |
| 2000 | 0.5 | 0.025 | Even | 20 | 1 | 5.52 | 4.80 | 4.82 | 4.82 |
| 2000 | 0.5 | 0.025 | Skewed | 4 | 1 | 5.30 | 4.85 | 4.93 | 4.86 |
| 2000 | 0.5 | 0.025 | Skewed | 20 | 1 | 5.94 | 5.61 | 5.69 | 5.60 |
| 2000 | 0.5 | 0.075 | Even | 4 | 1 | 5.62 | 5.16 | 5.16 | 5.16 |
| 2000 | 0.5 | 0.075 | Even | 20 | 1 | 5.50 | 5.00 | 5.00 | 4.96 |
| 2000 | 0.5 | 0.075 | Skewed | 4 | 1 | 5.83 | 5.42 | 5.42 | 5.40 |
| 2000 | 0.5 | 0.075 | Skewed | 20 | 1 | 5.55 | 4.92 | 4.94 | 5.08 |

**Table 33 – Convergence – 100 centres, true OR=1**

| Sample size | Event rate | ICC | Patient distribution | Block size | True OR | Fixed effects | Random effects | GEE | MH |
| --- | --- | --- | --- | --- | --- | --- | --- | --- | --- |
| 200 | 0.2 | 0.025 | Even | 4 | 1 | 99.44 | 99.94 | 100.00 | 100.00 |
| 200 | 0.2 | 0.025 | Even | 20 | 1 | 99.14 | 99.98 | 100.00 | 100.00 |
| 200 | 0.2 | 0.025 | Skewed | 4 | 1 | 100.00 | 100.00 | 100.00 | 100.00 |
| 200 | 0.2 | 0.025 | Skewed | 20 | 1 | 99.96 | 99.96 | 100.00 | 100.00 |
| 200 | 0.2 | 0.075 | Even | 4 | 1 | 99.28 | 99.90 | 100.00 | 99.98 |
| 200 | 0.2 | 0.075 | Even | 20 | 1 | 99.24 | 99.96 | 100.00 | 100.00 |
| 200 | 0.2 | 0.075 | Skewed | 4 | 1 | 99.96 | 99.88 | 100.00 | 100.00 |
| 200 | 0.2 | 0.075 | Skewed | 20 | 1 | 99.86 | 100.00 | 100.00 | 100.00 |
| 200 | 0.5 | 0.025 | Even | 4 | 1 | 99.90 | 99.80 | 100.00 | 99.98 |
| 200 | 0.5 | 0.025 | Even | 20 | 1 | 99.72 | 99.88 | 100.00 | 99.96 |
| 200 | 0.5 | 0.025 | Skewed | 4 | 1 | 99.94 | 99.94 | 100.00 | 99.94 |
| 200 | 0.5 | 0.025 | Skewed | 20 | 1 | 100.00 | 99.88 | 100.00 | 99.96 |
| 200 | 0.5 | 0.075 | Even | 4 | 1 | 99.84 | 99.84 | 100.00 | 99.92 |
| 200 | 0.5 | 0.075 | Even | 20 | 1 | 99.80 | 99.92 | 100.00 | 99.98 |
| 200 | 0.5 | 0.075 | Skewed | 4 | 1 | 99.98 | 99.98 | 100.00 | 100.00 |
| 200 | 0.5 | 0.075 | Skewed | 20 | 1 | 100.00 | 99.98 | 100.00 | 99.98 |
| 500 | 0.2 | 0.025 | Even | 4 | 1 | 100.00 | 99.88 | 100.00 | 100.00 |
| 500 | 0.2 | 0.025 | Even | 20 | 1 | 100.00 | 99.80 | 100.00 | 100.00 |
| 500 | 0.2 | 0.025 | Skewed | 4 | 1 | 99.90 | 99.98 | 100.00 | 100.00 |
| 500 | 0.2 | 0.025 | Skewed | 20 | 1 | 99.88 | 99.94 | 100.00 | 100.00 |
| 500 | 0.2 | 0.075 | Even | 4 | 1 | 100.00 | 99.96 | 100.00 | 100.00 |
| 500 | 0.2 | 0.075 | Even | 20 | 1 | 100.00 | 99.92 | 100.00 | 100.00 |
| 500 | 0.2 | 0.075 | Skewed | 4 | 1 | 99.84 | 99.98 | 100.00 | 100.00 |
| 500 | 0.2 | 0.075 | Skewed | 20 | 1 | 99.86 | 100.00 | 100.00 | 100.00 |
| 500 | 0.5 | 0.025 | Even | 4 | 1 | 100.00 | 100.00 | 100.00 | 100.00 |
| 500 | 0.5 | 0.025 | Even | 20 | 1 | 100.00 | 99.96 | 100.00 | 100.00 |
| 500 | 0.5 | 0.025 | Skewed | 4 | 1 | 100.00 | 99.94 | 100.00 | 100.00 |
| 500 | 0.5 | 0.025 | Skewed | 20 | 1 | 100.00 | 99.96 | 100.00 | 100.00 |
| 500 | 0.5 | 0.075 | Even | 4 | 1 | 100.00 | 100.00 | 100.00 | 100.00 |
| 500 | 0.5 | 0.075 | Even | 20 | 1 | 100.00 | 100.00 | 100.00 | 100.00 |
| 500 | 0.5 | 0.075 | Skewed | 4 | 1 | 99.98 | 100.00 | 100.00 | 100.00 |
| 500 | 0.5 | 0.075 | Skewed | 20 | 1 | 100.00 | 100.00 | 100.00 | 100.00 |
| 1000 | 0.2 | 0.025 | Even | 4 | 1 | 100.00 | 99.96 | 100.00 | 100.00 |
| 1000 | 0.2 | 0.025 | Even | 20 | 1 | 100.00 | 99.96 | 100.00 | 100.00 |
| 1000 | 0.2 | 0.025 | Skewed | 4 | 1 | 99.98 | 99.98 | 100.00 | 100.00 |
| 1000 | 0.2 | 0.025 | Skewed | 20 | 1 | 99.98 | 99.92 | 100.00 | 100.00 |
| 1000 | 0.2 | 0.075 | Even | 4 | 1 | 100.00 | 100.00 | 100.00 | 100.00 |
| 1000 | 0.2 | 0.075 | Even | 20 | 1 | 100.00 | 100.00 | 100.00 | 100.00 |
| 1000 | 0.2 | 0.075 | Skewed | 4 | 1 | 99.92 | 99.98 | 100.00 | 100.00 |
| 1000 | 0.2 | 0.075 | Skewed | 20 | 1 | 99.92 | 100.00 | 100.00 | 100.00 |
| 1000 | 0.5 | 0.025 | Even | 4 | 1 | 100.00 | 99.98 | 100.00 | 100.00 |
| 1000 | 0.5 | 0.025 | Even | 20 | 1 | 100.00 | 99.90 | 100.00 | 100.00 |
| 1000 | 0.5 | 0.025 | Skewed | 4 | 1 | 99.98 | 100.00 | 100.00 | 100.00 |
| 1000 | 0.5 | 0.025 | Skewed | 20 | 1 | 99.98 | 99.84 | 100.00 | 100.00 |
| 1000 | 0.5 | 0.075 | Even | 4 | 1 | 100.00 | 100.00 | 100.00 | 100.00 |
| 1000 | 0.5 | 0.075 | Even | 20 | 1 | 100.00 | 100.00 | 100.00 | 100.00 |
| 1000 | 0.5 | 0.075 | Skewed | 4 | 1 | 98.94 | 100.00 | 100.00 | 100.00 |
| 1000 | 0.5 | 0.075 | Skewed | 20 | 1 | 98.92 | 100.00 | 100.00 | 100.00 |
| 2000 | 0.2 | 0.025 | Even | 4 | 1 | 100.00 | 99.44 | 100.00 | 100.00 |
| 2000 | 0.2 | 0.025 | Even | 20 | 1 | 100.00 | 99.64 | 100.00 | 100.00 |
| 2000 | 0.2 | 0.025 | Skewed | 4 | 1 | 100.00 | 99.88 | 99.92 | 100.00 |
| 2000 | 0.2 | 0.025 | Skewed | 20 | 1 | 100.00 | 99.86 | 99.90 | 100.00 |
| 2000 | 0.2 | 0.075 | Even | 4 | 1 | 100.00 | 100.00 | 100.00 | 100.00 |
| 2000 | 0.2 | 0.075 | Even | 20 | 1 | 100.00 | 100.00 | 100.00 | 100.00 |
| 2000 | 0.2 | 0.075 | Skewed | 4 | 1 | 99.92 | 100.00 | 100.00 | 100.00 |
| 2000 | 0.2 | 0.075 | Skewed | 20 | 1 | 99.94 | 100.00 | 100.00 | 100.00 |
| 2000 | 0.5 | 0.025 | Even | 4 | 1 | 100.00 | 99.96 | 100.00 | 99.98 |
| 2000 | 0.5 | 0.025 | Even | 20 | 1 | 100.00 | 99.94 | 100.00 | 100.00 |
| 2000 | 0.5 | 0.025 | Skewed | 4 | 1 | 99.96 | 99.78 | 99.86 | 100.00 |
| 2000 | 0.5 | 0.025 | Skewed | 20 | 1 | 99.96 | 99.76 | 99.86 | 100.00 |
| 2000 | 0.5 | 0.075 | Even | 4 | 1 | 100.00 | 100.00 | 100.00 | 100.00 |
| 2000 | 0.5 | 0.075 | Even | 20 | 1 | 100.00 | 100.00 | 100.00 | 100.00 |
| 2000 | 0.5 | 0.075 | Skewed | 4 | 1 | 97.84 | 100.00 | 100.00 | 100.00 |
| 2000 | 0.5 | 0.075 | Skewed | 20 | 1 | 97.74 | 99.98 | 100.00 | 100.00 |

**Table 34 – Mean treatment effect – 100 centres, true OR<1**

| Sample size | Event rate | ICC | Patient distribution | Block size | True OR | Fixed effects | Random effects | GEE | MH |
| --- | --- | --- | --- | --- | --- | --- | --- | --- | --- |
| 200 | 0.2 | 0.025 | Even | 4 | 0.246 | 0.05 | 0.21 | 0.23 | 0.23 |
| 200 | 0.2 | 0.025 | Even | 20 | 0.246 | 0.06 | 0.22 | 0.23 | 0.25 |
| 200 | 0.2 | 0.025 | Skewed | 4 | 0.246 | 0.12 | 0.22 | 0.23 | 0.22 |
| 200 | 0.2 | 0.025 | Skewed | 20 | 0.246 | 0.13 | 0.22 | 0.24 | 0.23 |
| 200 | 0.2 | 0.075 | Even | 4 | 0.246 | 0.05 | 0.21 | 0.24 | 0.23 |
| 200 | 0.2 | 0.075 | Even | 20 | 0.246 | 0.06 | 0.22 | 0.24 | 0.24 |
| 200 | 0.2 | 0.075 | Skewed | 4 | 0.246 | 0.12 | 0.22 | 0.24 | 0.23 |
| 200 | 0.2 | 0.075 | Skewed | 20 | 0.246 | 0.13 | 0.22 | 0.24 | 0.23 |
| 200 | 0.5 | 0.025 | Even | 4 | 0.42 | 0.17 | 0.40 | 0.42 | 0.41 |
| 200 | 0.5 | 0.025 | Even | 20 | 0.42 | 0.16 | 0.40 | 0.42 | 0.40 |
| 200 | 0.5 | 0.025 | Skewed | 4 | 0.42 | 0.29 | 0.41 | 0.42 | 0.41 |
| 200 | 0.5 | 0.025 | Skewed | 20 | 0.42 | 0.29 | 0.41 | 0.42 | 0.40 |
| 200 | 0.5 | 0.075 | Even | 4 | 0.42 | 0.16 | 0.40 | 0.44 | 0.40 |
| 200 | 0.5 | 0.075 | Even | 20 | 0.42 | 0.16 | 0.41 | 0.44 | 0.40 |
| 200 | 0.5 | 0.075 | Skewed | 4 | 0.42 | 0.28 | 0.41 | 0.44 | 0.41 |
| 200 | 0.5 | 0.075 | Skewed | 20 | 0.42 | 0.29 | 0.41 | 0.44 | 0.41 |
| 500 | 0.2 | 0.025 | Even | 4 | 0.474 | 0.39 | 0.47 | 0.48 | 0.47 |
| 500 | 0.2 | 0.025 | Even | 20 | 0.474 | 0.39 | 0.47 | 0.47 | 0.47 |
| 500 | 0.2 | 0.025 | Skewed | 4 | 0.474 | 0.42 | 0.47 | 0.47 | 0.47 |
| 500 | 0.2 | 0.025 | Skewed | 20 | 0.474 | 0.43 | 0.47 | 0.48 | 0.47 |
| 500 | 0.2 | 0.075 | Even | 4 | 0.474 | 0.39 | 0.47 | 0.48 | 0.47 |
| 500 | 0.2 | 0.075 | Even | 20 | 0.474 | 0.39 | 0.47 | 0.49 | 0.47 |
| 500 | 0.2 | 0.075 | Skewed | 4 | 0.474 | 0.42 | 0.47 | 0.48 | 0.47 |
| 500 | 0.2 | 0.075 | Skewed | 20 | 0.474 | 0.43 | 0.47 | 0.48 | 0.47 |
| 500 | 0.5 | 0.025 | Even | 4 | 0.59 | 0.52 | 0.59 | 0.60 | 0.59 |
| 500 | 0.5 | 0.025 | Even | 20 | 0.59 | 0.51 | 0.59 | 0.59 | 0.59 |
| 500 | 0.5 | 0.025 | Skewed | 4 | 0.59 | 0.54 | 0.59 | 0.59 | 0.59 |
| 500 | 0.5 | 0.025 | Skewed | 20 | 0.59 | 0.55 | 0.59 | 0.60 | 0.59 |
| 500 | 0.5 | 0.075 | Even | 4 | 0.59 | 0.52 | 0.59 | 0.61 | 0.59 |
| 500 | 0.5 | 0.075 | Even | 20 | 0.59 | 0.52 | 0.59 | 0.61 | 0.59 |
| 500 | 0.5 | 0.075 | Skewed | 4 | 0.59 | 0.55 | 0.59 | 0.61 | 0.59 |
| 500 | 0.5 | 0.075 | Skewed | 20 | 0.59 | 0.55 | 0.59 | 0.61 | 0.59 |
| 1000 | 0.2 | 0.025 | Even | 4 | 0.608 | 0.57 | 0.61 | 0.61 | 0.61 |
| 1000 | 0.2 | 0.025 | Even | 20 | 0.608 | 0.57 | 0.61 | 0.61 | 0.61 |
| 1000 | 0.2 | 0.025 | Skewed | 4 | 0.608 | 0.58 | 0.61 | 0.61 | 0.61 |
| 1000 | 0.2 | 0.025 | Skewed | 20 | 0.608 | 0.59 | 0.61 | 0.61 | 0.61 |
| 1000 | 0.2 | 0.075 | Even | 4 | 0.608 | 0.57 | 0.61 | 0.62 | 0.61 |
| 1000 | 0.2 | 0.075 | Even | 20 | 0.608 | 0.57 | 0.60 | 0.61 | 0.60 |
| 1000 | 0.2 | 0.075 | Skewed | 4 | 0.608 | 0.58 | 0.60 | 0.61 | 0.60 |
| 1000 | 0.2 | 0.075 | Skewed | 20 | 0.608 | 0.58 | 0.60 | 0.61 | 0.60 |
| 1000 | 0.5 | 0.025 | Even | 4 | 0.695 | 0.67 | 0.70 | 0.70 | 0.70 |
| 1000 | 0.5 | 0.025 | Even | 20 | 0.695 | 0.67 | 0.70 | 0.70 | 0.70 |
| 1000 | 0.5 | 0.025 | Skewed | 4 | 0.695 | 0.68 | 0.69 | 0.70 | 0.69 |
| 1000 | 0.5 | 0.025 | Skewed | 20 | 0.695 | 0.68 | 0.69 | 0.70 | 0.69 |
| 1000 | 0.5 | 0.075 | Even | 4 | 0.695 | 0.66 | 0.69 | 0.71 | 0.69 |
| 1000 | 0.5 | 0.075 | Even | 20 | 0.695 | 0.67 | 0.69 | 0.71 | 0.69 |
| 1000 | 0.5 | 0.075 | Skewed | 4 | 0.695 | 0.68 | 0.70 | 0.71 | 0.70 |
| 1000 | 0.5 | 0.075 | Skewed | 20 | 0.695 | 0.68 | 0.70 | 0.71 | 0.69 |
| 2000 | 0.2 | 0.025 | Even | 4 | 0.711 | 0.70 | 0.71 | 0.72 | 0.71 |
| 2000 | 0.2 | 0.025 | Even | 20 | 0.711 | 0.70 | 0.71 | 0.71 | 0.71 |
| 2000 | 0.2 | 0.025 | Skewed | 4 | 0.711 | 0.70 | 0.71 | 0.74 | 0.71 |
| 2000 | 0.2 | 0.025 | Skewed | 20 | 0.711 | 0.70 | 0.71 | 0.84 | 0.71 |
| 2000 | 0.2 | 0.075 | Even | 4 | 0.711 | 0.70 | 0.71 | 0.72 | 0.71 |
| 2000 | 0.2 | 0.075 | Even | 20 | 0.711 | 0.70 | 0.71 | 0.72 | 0.71 |
| 2000 | 0.2 | 0.075 | Skewed | 4 | 0.711 | 0.70 | 0.71 | 0.71 | 0.71 |
| 2000 | 0.2 | 0.075 | Skewed | 20 | 0.711 | 0.70 | 0.71 | 0.76 | 0.71 |
| 2000 | 0.5 | 0.025 | Even | 4 | 0.77 | 0.76 | 0.77 | 0.77 | 0.77 |
| 2000 | 0.5 | 0.025 | Even | 20 | 0.77 | 0.76 | 0.77 | 0.77 | 0.77 |
| 2000 | 0.5 | 0.025 | Skewed | 4 | 0.77 | 0.76 | 0.77 | 0.77 | 0.77 |
| 2000 | 0.5 | 0.025 | Skewed | 20 | 0.77 | 0.77 | 0.77 | 0.77 | 0.77 |
| 2000 | 0.5 | 0.075 | Even | 4 | 0.77 | 0.76 | 0.77 | 0.78 | 0.77 |
| 2000 | 0.5 | 0.075 | Even | 20 | 0.77 | 0.76 | 0.77 | 0.78 | 0.77 |
| 2000 | 0.5 | 0.075 | Skewed | 4 | 0.77 | 0.76 | 0.77 | 0.77 | 0.77 |
| 2000 | 0.5 | 0.075 | Skewed | 20 | 0.77 | 0.76 | 0.77 | 0.77 | 0.77 |

**Table 35 – Power – 100 centres, true OR<1**

| Sample size | Event rate | ICC | Patient distribution | Block size | True OR | Fixed effects | Random effects | GEE | MH |
| --- | --- | --- | --- | --- | --- | --- | --- | --- | --- |
| 200 | 0.2 | 0.025 | Even | 4 | 0.246 | 87.13 | 85.74 | 86.10 | 65.58 |
| 200 | 0.2 | 0.025 | Even | 20 | 0.246 | 77.18 | 85.88 | 86.64 | 50.78 |
| 200 | 0.2 | 0.025 | Skewed | 4 | 0.246 | 76.80 | 87.32 | 87.47 | 62.96 |
| 200 | 0.2 | 0.025 | Skewed | 20 | 0.246 | 66.32 | 85.30 | 85.88 | 51.36 |
| 200 | 0.2 | 0.075 | Even | 4 | 0.246 | 87.72 | 86.04 | 86.98 | 67.94 |
| 200 | 0.2 | 0.075 | Even | 20 | 0.246 | 77.76 | 85.81 | 86.76 | 52.90 |
| 200 | 0.2 | 0.075 | Skewed | 4 | 0.246 | 77.50 | 86.73 | 87.07 | 63.50 |
| 200 | 0.2 | 0.075 | Skewed | 20 | 0.246 | 68.98 | 86.83 | 87.27 | 52.68 |
| 200 | 0.5 | 0.025 | Even | 4 | 0.42 | 83.31 | 83.09 | 83.70 | 65.00 |
| 200 | 0.5 | 0.025 | Even | 20 | 0.42 | 75.02 | 82.37 | 83.16 | 53.38 |
| 200 | 0.5 | 0.025 | Skewed | 4 | 0.42 | 74.37 | 83.03 | 83.40 | 60.84 |
| 200 | 0.5 | 0.025 | Skewed | 20 | 0.42 | 66.95 | 83.11 | 83.56 | 54.20 |
| 200 | 0.5 | 0.075 | Even | 4 | 0.42 | 83.50 | 80.85 | 81.76 | 63.92 |
| 200 | 0.5 | 0.075 | Even | 20 | 0.42 | 74.24 | 79.29 | 80.40 | 52.52 |
| 200 | 0.5 | 0.075 | Skewed | 4 | 0.42 | 72.36 | 80.34 | 80.88 | 60.32 |
| 200 | 0.5 | 0.075 | Skewed | 20 | 0.42 | 65.85 | 80.42 | 81.12 | 52.30 |
| 500 | 0.2 | 0.025 | Even | 4 | 0.474 | 86.40 | 82.86 | 82.64 | 81.46 |
| 500 | 0.2 | 0.025 | Even | 20 | 0.474 | 83.52 | 84.67 | 84.56 | 77.24 |
| 500 | 0.2 | 0.025 | Skewed | 4 | 0.474 | 82.76 | 83.96 | 83.90 | 78.66 |
| 500 | 0.2 | 0.025 | Skewed | 20 | 0.474 | 80.13 | 83.41 | 83.22 | 76.22 |
| 500 | 0.2 | 0.075 | Even | 4 | 0.474 | 87.94 | 84.32 | 84.36 | 83.06 |
| 500 | 0.2 | 0.075 | Even | 20 | 0.474 | 82.50 | 82.77 | 82.94 | 76.56 |
| 500 | 0.2 | 0.075 | Skewed | 4 | 0.474 | 83.38 | 84.25 | 84.16 | 80.06 |
| 500 | 0.2 | 0.075 | Skewed | 20 | 0.474 | 80.58 | 83.21 | 83.34 | 76.74 |
| 500 | 0.5 | 0.025 | Even | 4 | 0.59 | 85.66 | 82.17 | 82.14 | 80.00 |
| 500 | 0.5 | 0.025 | Even | 20 | 0.59 | 81.66 | 82.27 | 82.50 | 74.98 |
| 500 | 0.5 | 0.025 | Skewed | 4 | 0.59 | 81.51 | 82.86 | 82.84 | 77.92 |
| 500 | 0.5 | 0.025 | Skewed | 20 | 0.59 | 78.09 | 81.42 | 81.54 | 74.70 |
| 500 | 0.5 | 0.075 | Even | 4 | 0.59 | 84.98 | 80.58 | 80.70 | 79.20 |
| 500 | 0.5 | 0.075 | Even | 20 | 0.59 | 79.12 | 78.81 | 79.00 | 72.66 |
| 500 | 0.5 | 0.075 | Skewed | 4 | 0.59 | 78.93 | 79.86 | 79.92 | 75.08 |
| 500 | 0.5 | 0.075 | Skewed | 20 | 0.59 | 76.90 | 79.48 | 79.52 | 72.90 |
| 1000 | 0.2 | 0.025 | Even | 4 | 0.608 | 85.00 | 83.29 | 83.22 | 82.86 |
| 1000 | 0.2 | 0.025 | Even | 20 | 0.608 | 83.58 | 82.69 | 82.62 | 80.70 |
| 1000 | 0.2 | 0.025 | Skewed | 4 | 0.608 | 82.62 | 82.01 | 81.98 | 80.54 |
| 1000 | 0.2 | 0.025 | Skewed | 20 | 0.608 | 81.89 | 82.51 | 82.48 | 80.16 |
| 1000 | 0.2 | 0.075 | Even | 4 | 0.608 | 85.38 | 82.78 | 82.86 | 82.60 |
| 1000 | 0.2 | 0.075 | Even | 20 | 0.608 | 84.08 | 83.02 | 82.96 | 81.36 |
| 1000 | 0.2 | 0.075 | Skewed | 4 | 0.608 | 83.77 | 83.20 | 83.14 | 81.76 |
| 1000 | 0.2 | 0.075 | Skewed | 20 | 0.608 | 82.12 | 83.46 | 83.30 | 80.22 |
| 1000 | 0.5 | 0.025 | Even | 4 | 0.695 | 82.12 | 79.73 | 79.74 | 79.10 |
| 1000 | 0.5 | 0.025 | Even | 20 | 0.695 | 81.10 | 79.76 | 79.72 | 78.16 |
| 1000 | 0.5 | 0.025 | Skewed | 4 | 0.695 | 80.10 | 80.00 | 80.04 | 78.24 |
| 1000 | 0.5 | 0.025 | Skewed | 20 | 0.695 | 79.57 | 80.36 | 80.42 | 77.64 |
| 1000 | 0.5 | 0.075 | Even | 4 | 0.695 | 82.48 | 79.76 | 79.80 | 79.22 |
| 1000 | 0.5 | 0.075 | Even | 20 | 0.695 | 80.24 | 79.02 | 79.08 | 77.10 |
| 1000 | 0.5 | 0.075 | Skewed | 4 | 0.695 | 78.98 | 78.64 | 78.80 | 76.68 |
| 1000 | 0.5 | 0.075 | Skewed | 20 | 0.695 | 77.11 | 78.11 | 78.20 | 75.28 |
| 2000 | 0.2 | 0.025 | Even | 4 | 0.711 | 83.08 | 81.68 | 81.70 | 81.58 |
| 2000 | 0.2 | 0.025 | Even | 20 | 0.711 | 84.08 | 82.72 | 82.70 | 82.64 |
| 2000 | 0.2 | 0.025 | Skewed | 4 | 0.711 | 82.55 | 82.17 | 82.17 | 81.48 |
| 2000 | 0.2 | 0.025 | Skewed | 20 | 0.711 | 82.34 | 82.40 | 82.39 | 81.30 |
| 2000 | 0.2 | 0.075 | Even | 4 | 0.711 | 84.08 | 82.70 | 82.70 | 82.70 |
| 2000 | 0.2 | 0.075 | Even | 20 | 0.711 | 83.56 | 82.24 | 82.24 | 82.20 |
| 2000 | 0.2 | 0.075 | Skewed | 4 | 0.711 | 82.59 | 81.92 | 81.92 | 81.28 |
| 2000 | 0.2 | 0.075 | Skewed | 20 | 0.711 | 82.12 | 82.40 | 82.40 | 81.18 |
| 2000 | 0.5 | 0.025 | Even | 4 | 0.77 | 83.62 | 82.51 | 82.54 | 82.52 |
| 2000 | 0.5 | 0.025 | Even | 20 | 0.77 | 82.82 | 81.57 | 81.60 | 81.56 |
| 2000 | 0.5 | 0.025 | Skewed | 4 | 0.77 | 82.42 | 82.14 | 82.15 | 81.38 |
| 2000 | 0.5 | 0.025 | Skewed | 20 | 0.77 | 80.84 | 81.04 | 81.04 | 79.78 |
| 2000 | 0.5 | 0.075 | Even | 4 | 0.77 | 82.54 | 81.02 | 81.00 | 80.96 |
| 2000 | 0.5 | 0.075 | Even | 20 | 0.77 | 83.06 | 81.75 | 81.78 | 81.70 |
| 2000 | 0.5 | 0.075 | Skewed | 4 | 0.77 | 80.88 | 80.45 | 80.46 | 79.76 |
| 2000 | 0.5 | 0.075 | Skewed | 20 | 0.77 | 80.02 | 80.06 | 80.06 | 78.94 |

**Table 36 – Convergence – 100 centres, true OR<1**

| Sample size | Event rate | ICC | Patient distribution | Block size | True OR | Fixed effects | Random effects | GEE | MH |
| --- | --- | --- | --- | --- | --- | --- | --- | --- | --- |
| 200 | 0.2 | 0.025 | Even | 4 | 0.246 | 94.30 | 99.74 | 100.00 | 100.00 |
| 200 | 0.2 | 0.025 | Even | 20 | 0.246 | 91.84 | 99.74 | 100.00 | 100.00 |
| 200 | 0.2 | 0.025 | Skewed | 4 | 0.246 | 96.56 | 99.70 | 99.92 | 100.00 |
| 200 | 0.2 | 0.025 | Skewed | 20 | 0.246 | 94.96 | 99.56 | 99.88 | 100.00 |
| 200 | 0.2 | 0.075 | Even | 4 | 0.246 | 94.00 | 99.86 | 99.98 | 100.00 |
| 200 | 0.2 | 0.075 | Even | 20 | 0.246 | 91.44 | 99.76 | 100.00 | 100.00 |
| 200 | 0.2 | 0.075 | Skewed | 4 | 0.246 | 97.06 | 99.76 | 99.96 | 100.00 |
| 200 | 0.2 | 0.075 | Skewed | 20 | 0.246 | 95.30 | 99.74 | 99.96 | 100.00 |
| 200 | 0.5 | 0.025 | Even | 4 | 0.42 | 97.80 | 99.94 | 100.00 | 100.00 |
| 200 | 0.5 | 0.025 | Even | 20 | 0.42 | 97.58 | 99.92 | 100.00 | 100.00 |
| 200 | 0.5 | 0.025 | Skewed | 4 | 0.42 | 99.90 | 99.96 | 100.00 | 100.00 |
| 200 | 0.5 | 0.025 | Skewed | 20 | 0.42 | 99.96 | 99.96 | 100.00 | 100.00 |
| 200 | 0.5 | 0.075 | Even | 4 | 0.42 | 97.94 | 99.94 | 100.00 | 100.00 |
| 200 | 0.5 | 0.075 | Even | 20 | 0.42 | 97.60 | 99.96 | 100.00 | 100.00 |
| 200 | 0.5 | 0.075 | Skewed | 4 | 0.42 | 99.94 | 99.98 | 100.00 | 100.00 |
| 200 | 0.5 | 0.075 | Skewed | 20 | 0.42 | 99.92 | 100.00 | 100.00 | 100.00 |
| 500 | 0.2 | 0.025 | Even | 4 | 0.474 | 100.00 | 99.98 | 100.00 | 100.00 |
| 500 | 0.2 | 0.025 | Even | 20 | 0.474 | 99.98 | 99.96 | 100.00 | 100.00 |
| 500 | 0.2 | 0.025 | Skewed | 4 | 0.474 | 99.78 | 99.90 | 100.00 | 100.00 |
| 500 | 0.2 | 0.025 | Skewed | 20 | 0.474 | 99.84 | 99.94 | 100.00 | 100.00 |
| 500 | 0.2 | 0.075 | Even | 4 | 0.474 | 99.96 | 99.90 | 100.00 | 100.00 |
| 500 | 0.2 | 0.075 | Even | 20 | 0.474 | 100.00 | 99.94 | 100.00 | 100.00 |
| 500 | 0.2 | 0.075 | Skewed | 4 | 0.474 | 99.86 | 99.96 | 100.00 | 100.00 |
| 500 | 0.2 | 0.075 | Skewed | 20 | 0.474 | 99.92 | 99.96 | 100.00 | 100.00 |
| 500 | 0.5 | 0.025 | Even | 4 | 0.59 | 100.00 | 99.92 | 100.00 | 100.00 |
| 500 | 0.5 | 0.025 | Even | 20 | 0.59 | 100.00 | 99.94 | 100.00 | 100.00 |
| 500 | 0.5 | 0.025 | Skewed | 4 | 0.59 | 99.96 | 99.86 | 100.00 | 100.00 |
| 500 | 0.5 | 0.025 | Skewed | 20 | 0.59 | 99.96 | 99.90 | 100.00 | 100.00 |
| 500 | 0.5 | 0.075 | Even | 4 | 0.59 | 100.00 | 99.98 | 100.00 | 100.00 |
| 500 | 0.5 | 0.075 | Even | 20 | 0.59 | 100.00 | 99.96 | 100.00 | 100.00 |
| 500 | 0.5 | 0.075 | Skewed | 4 | 0.59 | 99.94 | 100.00 | 100.00 | 100.00 |
| 500 | 0.5 | 0.075 | Skewed | 20 | 0.59 | 100.00 | 99.98 | 100.00 | 100.00 |
| 1000 | 0.2 | 0.025 | Even | 4 | 0.608 | 100.00 | 99.80 | 100.00 | 100.00 |
| 1000 | 0.2 | 0.025 | Even | 20 | 0.608 | 100.00 | 99.82 | 100.00 | 100.00 |
| 1000 | 0.2 | 0.025 | Skewed | 4 | 0.608 | 99.88 | 99.84 | 100.00 | 100.00 |
| 1000 | 0.2 | 0.025 | Skewed | 20 | 0.608 | 99.86 | 99.92 | 100.00 | 100.00 |
| 1000 | 0.2 | 0.075 | Even | 4 | 0.608 | 100.00 | 99.98 | 100.00 | 100.00 |
| 1000 | 0.2 | 0.075 | Even | 20 | 0.608 | 100.00 | 99.98 | 100.00 | 100.00 |
| 1000 | 0.2 | 0.075 | Skewed | 4 | 0.608 | 99.70 | 100.00 | 100.00 | 100.00 |
| 1000 | 0.2 | 0.075 | Skewed | 20 | 0.608 | 99.88 | 99.98 | 100.00 | 100.00 |
| 1000 | 0.5 | 0.025 | Even | 4 | 0.695 | 100.00 | 99.96 | 100.00 | 100.00 |
| 1000 | 0.5 | 0.025 | Even | 20 | 0.695 | 100.00 | 99.98 | 100.00 | 100.00 |
| 1000 | 0.5 | 0.025 | Skewed | 4 | 0.695 | 99.60 | 99.80 | 100.00 | 100.00 |
| 1000 | 0.5 | 0.025 | Skewed | 20 | 0.695 | 99.58 | 99.80 | 100.00 | 100.00 |
| 1000 | 0.5 | 0.075 | Even | 4 | 0.695 | 100.00 | 100.00 | 100.00 | 100.00 |
| 1000 | 0.5 | 0.075 | Even | 20 | 0.695 | 100.00 | 100.00 | 100.00 | 100.00 |
| 1000 | 0.5 | 0.075 | Skewed | 4 | 0.695 | 97.98 | 100.00 | 100.00 | 100.00 |
| 1000 | 0.5 | 0.075 | Skewed | 20 | 0.695 | 98.14 | 99.96 | 100.00 | 100.00 |
| 2000 | 0.2 | 0.025 | Even | 4 | 0.711 | 100.00 | 99.66 | 100.00 | 100.00 |
| 2000 | 0.2 | 0.025 | Even | 20 | 0.711 | 100.00 | 99.68 | 100.00 | 100.00 |
| 2000 | 0.2 | 0.025 | Skewed | 4 | 0.711 | 99.72 | 99.84 | 99.96 | 100.00 |
| 2000 | 0.2 | 0.025 | Skewed | 20 | 0.711 | 99.76 | 99.86 | 99.92 | 100.00 |
| 2000 | 0.2 | 0.075 | Even | 4 | 0.711 | 100.00 | 100.00 | 100.00 | 100.00 |
| 2000 | 0.2 | 0.075 | Even | 20 | 0.711 | 100.00 | 100.00 | 100.00 | 100.00 |
| 2000 | 0.2 | 0.075 | Skewed | 4 | 0.711 | 99.72 | 100.00 | 100.00 | 100.00 |
| 2000 | 0.2 | 0.075 | Skewed | 20 | 0.711 | 99.66 | 99.98 | 100.00 | 100.00 |
| 2000 | 0.5 | 0.025 | Even | 4 | 0.77 | 100.00 | 99.92 | 100.00 | 100.00 |
| 2000 | 0.5 | 0.025 | Even | 20 | 0.77 | 100.00 | 99.82 | 100.00 | 100.00 |
| 2000 | 0.5 | 0.025 | Skewed | 4 | 0.77 | 99.80 | 99.76 | 99.82 | 100.00 |
| 2000 | 0.5 | 0.025 | Skewed | 20 | 0.77 | 99.70 | 99.88 | 99.90 | 100.00 |
| 2000 | 0.5 | 0.075 | Even | 4 | 0.77 | 100.00 | 99.98 | 100.00 | 100.00 |
| 2000 | 0.5 | 0.075 | Even | 20 | 0.77 | 100.00 | 99.92 | 100.00 | 100.00 |
| 2000 | 0.5 | 0.075 | Skewed | 4 | 0.77 | 97.92 | 99.96 | 99.98 | 100.00 |
| 2000 | 0.5 | 0.075 | Skewed | 20 | 0.77 | 97.68 | 99.98 | 99.98 | 100.00 |

**4 - Results from sensitivity analysis – large ICC**

***Results from individual scenarios***

**Table 37 – Mean treatment effect – true OR=1**

| Number of centres | Sample size | Block size | True OR | Fixed effects | Random effects | GEE | MH |
| --- | --- | --- | --- | --- | --- | --- | --- |
| 5 | 200 | 4 | 1 | 1.00 | 1.00 | 1.00 | 1.00 |
| 5 | 200 | 20 | 1 | 0.99 | 0.99 | 0.99 | 0.99 |
| 5 | 500 | 4 | 1 | 1.00 | 1.00 | 1.00 | 1.00 |
| 5 | 500 | 20 | 1 | 1.00 | 1.00 | 1.00 | 1.00 |
| 5 | 1000 | 4 | 1 | 1.00 | 1.00 | 1.00 | 1.00 |
| 5 | 1000 | 20 | 1 | 1.00 | 1.00 | 1.00 | 1.00 |
| 5 | 2000 | 4 | 1 | 1.00 | 1.00 | 1.00 | 1.00 |
| 5 | 2000 | 20 | 1 | 1.00 | 1.00 | 1.00 | 1.00 |
| 50 | 200 | 4 | 1 | 1.00 | 1.00 | 1.00 | 1.00 |
| 50 | 200 | 20 | 1 | 1.00 | 1.00 | 1.00 | 1.00 |
| 50 | 500 | 4 | 1 | 1.00 | 1.00 | 1.00 | 1.00 |
| 50 | 500 | 20 | 1 | 1.00 | 1.00 | 1.00 | 1.00 |
| 50 | 1000 | 4 | 1 | 1.00 | 1.00 | 1.00 | 1.00 |
| 50 | 1000 | 20 | 1 | 1.00 | 1.00 | 1.00 | 1.00 |
| 50 | 2000 | 4 | 1 | 1.00 | 1.00 | 1.00 | 1.00 |
| 50 | 2000 | 20 | 1 | 1.00 | 1.00 | 1.00 | 1.00 |
| 100 | 200 | 4 | 1 | 1.01 | 1.00 | 1.00 | 1.00 |
| 100 | 200 | 20 | 1 | 1.00 | 1.00 | 1.00 | 1.00 |
| 100 | 500 | 4 | 1 | 1.00 | 1.00 | 1.00 | 1.00 |
| 100 | 500 | 20 | 1 | 1.00 | 1.00 | 1.00 | 1.00 |
| 100 | 1000 | 4 | 1 | 1.00 | 1.00 | 1.00 | 1.00 |
| 100 | 1000 | 20 | 1 | 1.00 | 1.00 | 1.00 | 1.00 |
| 100 | 2000 | 4 | 1 | 1.00 | 1.00 | 1.00 | 1.00 |
| 100 | 2000 | 20 | 1 | 1.00 | 1.00 | 1.00 | 1.00 |

**Table 38 – Type I error rate – true OR=1**

| Number of centres | Sample size | Block size | True OR | Fixed effects | Random effects | GEE | MH |
| --- | --- | --- | --- | --- | --- | --- | --- |
| 5 | 200 | 4 | 1 | 5.58 | 5.20 | 5.24 | 4.94 |
| 5 | 200 | 20 | 1 | 4.98 | 4.72 | 4.72 | 4.37 |
| 5 | 500 | 4 | 1 | 5.24 | 5.06 | 5.06 | 5.00 |
| 5 | 500 | 20 | 1 | 4.86 | 4.81 | 4.76 | 4.64 |
| 5 | 1000 | 4 | 1 | 4.70 | 4.64 | 4.62 | 4.58 |
| 5 | 1000 | 20 | 1 | 4.96 | 4.86 | 4.78 | 4.80 |
| 5 | 2000 | 4 | 1 | 5.08 | 5.06 | 4.96 | 5.04 |
| 5 | 2000 | 20 | 1 | 4.96 | 4.90 | 4.88 | 4.88 |
| 50 | 200 | 4 | 1 | 8.43 | 4.78 | 4.98 | 4.59 |
| 50 | 200 | 20 | 1 | 8.87 | 4.60 | 4.94 | 4.66 |
| 50 | 500 | 4 | 1 | 5.34 | 3.84 | 3.92 | 3.82 |
| 50 | 500 | 20 | 1 | 6.04 | 4.62 | 4.68 | 4.58 |
| 50 | 1000 | 4 | 1 | 5.38 | 4.66 | 4.68 | 4.60 |
| 50 | 1000 | 20 | 1 | 5.08 | 4.68 | 4.70 | 4.64 |
| 50 | 2000 | 4 | 1 | 5.24 | 4.86 | 4.88 | 4.82 |
| 50 | 2000 | 20 | 1 | 4.88 | 4.64 | 4.64 | 4.64 |
| 100 | 200 | 4 | 1 | 15.27 | 4.86 | 5.54 | 5.02 |
| 100 | 200 | 20 | 1 | 15.63 | 4.10 | 4.56 | 4.18 |
| 100 | 500 | 4 | 1 | 7.54 | 4.92 | 5.00 | 4.82 |
| 100 | 500 | 20 | 1 | 7.92 | 4.72 | 4.84 | 4.84 |
| 100 | 1000 | 4 | 1 | 5.88 | 4.74 | 4.80 | 4.54 |
| 100 | 1000 | 20 | 1 | 6.20 | 4.92 | 4.92 | 5.02 |
| 100 | 2000 | 4 | 1 | 5.62 | 5.12 | 5.12 | 5.06 |
| 100 | 2000 | 20 | 1 | 5.66 | 5.14 | 5.14 | 5.12 |

**Table 39 – Convergence – true OR=1**

| Number of centres | Sample size | Block size | True OR | Fixed effects | Random effects | GEE | MH |
| --- | --- | --- | --- | --- | --- | --- | --- |
| 5 | 200 | 4 | 1 | 99.98 | 99.94 | 100.00 | 99.94 |
| 5 | 200 | 20 | 1 | 99.96 | 99.96 | 100.00 | 99.86 |
| 5 | 500 | 4 | 1 | 100.00 | 99.94 | 100.00 | 99.98 |
| 5 | 500 | 20 | 1 | 100.00 | 99.88 | 100.00 | 100.00 |
| 5 | 1000 | 4 | 1 | 99.98 | 99.98 | 100.00 | 100.00 |
| 5 | 1000 | 20 | 1 | 99.98 | 99.96 | 100.00 | 100.00 |
| 5 | 2000 | 4 | 1 | 100.00 | 99.98 | 100.00 | 100.00 |
| 5 | 2000 | 20 | 1 | 100.00 | 99.92 | 100.00 | 100.00 |
| 50 | 200 | 4 | 1 | 99.94 | 100.00 | 100.00 | 99.88 |
| 50 | 200 | 20 | 1 | 99.94 | 100.00 | 100.00 | 100.00 |
| 50 | 500 | 4 | 1 | 100.00 | 100.00 | 100.00 | 100.00 |
| 50 | 500 | 20 | 1 | 100.00 | 100.00 | 100.00 | 100.00 |
| 50 | 1000 | 4 | 1 | 100.00 | 100.00 | 100.00 | 99.96 |
| 50 | 1000 | 20 | 1 | 100.00 | 100.00 | 100.00 | 100.00 |
| 50 | 2000 | 4 | 1 | 100.00 | 100.00 | 100.00 | 100.00 |
| 50 | 2000 | 20 | 1 | 100.00 | 100.00 | 100.00 | 100.00 |
| 100 | 200 | 4 | 1 | 99.70 | 100.00 | 100.00 | 99.98 |
| 100 | 200 | 20 | 1 | 99.58 | 100.00 | 100.00 | 100.00 |
| 100 | 500 | 4 | 1 | 100.00 | 100.00 | 100.00 | 100.00 |
| 100 | 500 | 20 | 1 | 100.00 | 100.00 | 100.00 | 100.00 |
| 100 | 1000 | 4 | 1 | 100.00 | 100.00 | 100.00 | 100.00 |
| 100 | 1000 | 20 | 1 | 100.00 | 100.00 | 100.00 | 100.00 |
| 100 | 2000 | 4 | 1 | 100.00 | 100.00 | 100.00 | 100.00 |
| 100 | 2000 | 20 | 1 | 100.00 | 100.00 | 100.00 | 100.00 |

**Table 40 – Mean treatment effect – true OR<1**

| Number of centres | Sample size | Block size | True OR | Fixed effects | Random effects | GEE | MH |
| --- | --- | --- | --- | --- | --- | --- | --- |
| 5 | 200 | 4 | 0.42 | 0.41 | 0.42 | 0.47 | 0.41 |
| 5 | 200 | 20 | 0.42 | 0.40 | 0.41 | 0.47 | 0.41 |
| 5 | 500 | 4 | 0.59 | 0.59 | 0.59 | 0.64 | 0.59 |
| 5 | 500 | 20 | 0.59 | 0.59 | 0.59 | 0.64 | 0.59 |
| 5 | 1000 | 4 | 0.695 | 0.69 | 0.70 | 0.74 | 0.70 |
| 5 | 1000 | 20 | 0.695 | 0.69 | 0.69 | 0.73 | 0.69 |
| 5 | 2000 | 4 | 0.77 | 0.77 | 0.77 | 0.80 | 0.77 |
| 5 | 2000 | 20 | 0.77 | 0.77 | 0.77 | 0.80 | 0.77 |
| 50 | 200 | 4 | 0.42 | 0.31 | 0.42 | 0.49 | 0.41 |
| 50 | 200 | 20 | 0.42 | 0.30 | 0.41 | 0.49 | 0.41 |
| 50 | 500 | 4 | 0.59 | 0.56 | 0.59 | 0.65 | 0.59 |
| 50 | 500 | 20 | 0.59 | 0.55 | 0.59 | 0.65 | 0.59 |
| 50 | 1000 | 4 | 0.695 | 0.68 | 0.70 | 0.74 | 0.70 |
| 50 | 1000 | 20 | 0.695 | 0.68 | 0.70 | 0.74 | 0.70 |
| 50 | 2000 | 4 | 0.77 | 0.76 | 0.77 | 0.81 | 0.77 |
| 50 | 2000 | 20 | 0.77 | 0.76 | 0.77 | 0.81 | 0.77 |
| 100 | 200 | 4 | 0.42 | 0.16 | 0.41 | 0.49 | 0.40 |
| 100 | 200 | 20 | 0.42 | 0.16 | 0.41 | 0.49 | 0.40 |
| 100 | 500 | 4 | 0.59 | 0.51 | 0.59 | 0.65 | 0.59 |
| 100 | 500 | 20 | 0.59 | 0.51 | 0.59 | 0.65 | 0.59 |
| 100 | 1000 | 4 | 0.695 | 0.67 | 0.69 | 0.74 | 0.69 |
| 100 | 1000 | 20 | 0.695 | 0.67 | 0.69 | 0.74 | 0.69 |
| 100 | 2000 | 4 | 0.77 | 0.76 | 0.77 | 0.81 | 0.77 |
| 100 | 2000 | 20 | 0.77 | 0.76 | 0.77 | 0.81 | 0.77 |

**Table 41 – Power – true OR<1**

| Number of centres | Sample size | Block size | True OR | Fixed effects | Random effects | GEE | MH |
| --- | --- | --- | --- | --- | --- | --- | --- |
| 5 | 200 | 4 | 0.42 | 76.89 | 76.10 | 76.36 | 75.64 |
| 5 | 200 | 20 | 0.42 | 77.86 | 77.32 | 77.26 | 76.88 |
| 5 | 500 | 4 | 0.59 | 75.16 | 74.70 | 74.64 | 74.52 |
| 5 | 500 | 20 | 0.59 | 74.24 | 73.90 | 73.82 | 73.66 |
| 5 | 1000 | 4 | 0.695 | 73.06 | 72.81 | 72.68 | 72.68 |
| 5 | 1000 | 20 | 0.695 | 74.48 | 74.36 | 74.26 | 74.32 |
| 5 | 2000 | 4 | 0.77 | 75.28 | 75.15 | 75.10 | 75.14 |
| 5 | 2000 | 20 | 0.77 | 75.34 | 75.20 | 75.06 | 75.14 |
| 50 | 200 | 4 | 0.42 | 84.16 | 76.30 | 76.76 | 75.72 |
| 50 | 200 | 20 | 0.42 | 76.08 | 72.20 | 73.18 | 65.60 |
| 50 | 500 | 4 | 0.59 | 77.71 | 74.88 | 75.02 | 74.12 |
| 50 | 500 | 20 | 0.59 | 76.24 | 74.16 | 74.36 | 72.80 |
| 50 | 1000 | 4 | 0.695 | 74.75 | 73.16 | 73.22 | 72.92 |
| 50 | 1000 | 20 | 0.695 | 74.62 | 72.94 | 73.08 | 72.82 |
| 50 | 2000 | 4 | 0.77 | 76.04 | 75.14 | 75.18 | 75.10 |
| 50 | 2000 | 20 | 0.77 | 75.62 | 74.84 | 74.84 | 74.74 |
| 100 | 200 | 4 | 0.42 | 78.70 | 71.42 | 73.50 | 57.40 |
| 100 | 200 | 20 | 0.42 | 70.58 | 68.40 | 70.68 | 47.96 |
| 100 | 500 | 4 | 0.59 | 80.10 | 74.34 | 74.84 | 73.62 |
| 100 | 500 | 20 | 0.59 | 75.24 | 70.90 | 71.28 | 67.70 |
| 100 | 1000 | 4 | 0.695 | 76.80 | 73.98 | 74.06 | 73.62 |
| 100 | 1000 | 20 | 0.695 | 74.94 | 72.06 | 72.24 | 71.44 |
| 100 | 2000 | 4 | 0.77 | 77.06 | 75.58 | 75.64 | 75.52 |
| 100 | 2000 | 20 | 0.77 | 77.56 | 75.98 | 76.02 | 75.94 |

**Table 42 – Convergence – true OR<1**

| Number of centres | Sample size | Block size | True OR | Fixed effects | Random effects | GEE | MH |
| --- | --- | --- | --- | --- | --- | --- | --- |
| 5 | 200 | 4 | 0.42 | 99.94 | 100.00 | 100.00 | 100.00 |
| 5 | 200 | 20 | 0.42 | 99.92 | 99.96 | 100.00 | 100.00 |
| 5 | 500 | 4 | 0.59 | 99.98 | 100.00 | 100.00 | 100.00 |
| 5 | 500 | 20 | 0.59 | 99.98 | 99.92 | 100.00 | 100.00 |
| 5 | 1000 | 4 | 0.695 | 100.00 | 99.98 | 100.00 | 100.00 |
| 5 | 1000 | 20 | 0.695 | 100.00 | 99.92 | 100.00 | 100.00 |
| 5 | 2000 | 4 | 0.77 | 100.00 | 99.96 | 100.00 | 100.00 |
| 5 | 2000 | 20 | 0.77 | 100.00 | 99.96 | 100.00 | 100.00 |
| 50 | 200 | 4 | 0.42 | 99.88 | 99.98 | 100.00 | 100.00 |
| 50 | 200 | 20 | 0.42 | 99.92 | 99.96 | 100.00 | 100.00 |
| 50 | 500 | 4 | 0.59 | 99.94 | 100.00 | 100.00 | 100.00 |
| 50 | 500 | 20 | 0.59 | 99.98 | 100.00 | 100.00 | 100.00 |
| 50 | 1000 | 4 | 0.695 | 99.98 | 100.00 | 100.00 | 100.00 |
| 50 | 1000 | 20 | 0.695 | 100.00 | 100.00 | 100.00 | 100.00 |
| 50 | 2000 | 4 | 0.77 | 100.00 | 100.00 | 100.00 | 100.00 |
| 50 | 2000 | 20 | 0.77 | 100.00 | 100.00 | 100.00 | 100.00 |
| 100 | 200 | 4 | 0.42 | 97.64 | 100.00 | 100.00 | 100.00 |
| 100 | 200 | 20 | 0.42 | 97.48 | 99.96 | 100.00 | 100.00 |
| 100 | 500 | 4 | 0.59 | 100.00 | 100.00 | 100.00 | 100.00 |
| 100 | 500 | 20 | 0.59 | 100.00 | 100.00 | 100.00 | 100.00 |
| 100 | 1000 | 4 | 0.695 | 100.00 | 100.00 | 100.00 | 100.00 |
| 100 | 1000 | 20 | 0.695 | 100.00 | 100.00 | 100.00 | 100.00 |
| 100 | 2000 | 4 | 0.77 | 100.00 | 100.00 | 100.00 | 100.00 |
| 100 | 2000 | 20 | 0.77 | 100.00 | 100.00 | 100.00 | 100.00 |
